# Eradication of MRSA biofilm-associated implant infections by low-immunogenic sustained-release lysostaphin fused to thermosensitive polypeptides

**DOI:** 10.1038/s41467-026-76664-4

**Published:** 2026-08-14

**Authors:** Guoqing Fan, Hao Wang, Yuxin Gong, Si Liu, Yuanzi Sun, Jie Tian, Hongbin Wang, Litao Zhang, Fan Zhang, Like Gong, Lin Wang, Fengmin Lu, Yongping Cao, Wanliang Lu, Mingming Xu, Shengtao Zhu, Weiping Gao

**Affiliations:** 1 Beijing Advanced Center of Cellular Homeostasis and Aging-Related Diseases, Department of Biomedical Engineering, Institute of Advanced Clinical Medicine, Peking University, Beijing, China; 2 State Key Laboratory of Natural and Biomimetic Drugs, Beijing Key Laboratory of Advanced Pharmaceutical Preparation, School of Pharmaceutical Sciences, Peking University, Beijing, China; 3 Peking University-Yunnan Baiyao International Medical Research Center, Peking University International Cancer Institute, Beijing, China; 4 Department of Orthopedics, Peking University First Hospital, Beijing, China; 5 Department of Gastroenterology, Beijing Friendship Hospital, Capital Medical University, State Key Laboratory of Digestive Health, National Clinical Research Center for Digestive Disease, Beijing, China; 6 Department of Dermatology and Venereology, Peking University First Hospital, Beijing, China; 7 Department of Microbiology & Infectious Disease Center, School of Basic Medical Sciences, Peking University, Beijing, China; 8 Department of Geriatric Dentistry, Peking University School and Hospital of Stomatology, & National Center for Stomatology & National Clinical Research Center for Oral Diseases & National Engineering Research Center of Oral Biomaterials and Digital Medical Devices, Beijing, China

**Keywords:** Antimicrobial resistance, Protein delivery, Recombinant protein therapy

## Abstract

Staphylococcal bacteria, particularly methicillin-resistant *Staphylococcus aureus* (MRSA), are responsible for intractable infections—especially those associated with implants—through biofilm formation, presenting a worldwide menace. Antibacterial enzymes like lysostaphin (Lst) are promising for combating staphylococcal infections but limited by poor stability, high immunogenicity, and suboptimal pharmacokinetics, which hamper their clinical translation as antibiotic alternatives. Herein, we report a strategy of fusing thermosensitive elastin-like polypeptides (ELPs) to Lst to overcome these inherent drawbacks and eradicate MRSA biofilm-induced implant infections. Guided by AlphaFold2, we rationally engineered a chimeric Lst-ELP fusion protein that preserves potent lytic activity, demonstrates markedly enhanced stability, and exhibits substantially reduced immunogenicity relative to free Lst. The thermosensitivity of Lst-ELP enables in situ formation of a sustained-release depot upon subcutaneous injection, translating to an increased maximum tolerated dose, improved pharmacokinetics, and optimized biodistribution. Consequently, a single injection of Lst-ELP not only fully eradicated MRSA biofilms on implants and MRSA from the bloodstream and wound tissues, but also eliminated local and systemic inflammatory responses. This approach achieved complete clearance of MRSA biofilm-associated implant infections without adverse effects, highlighting its potential for clinical translation.

## Abstract (web summary)

Authors present an antimicrobial characterisation of their elastin-like polypeptide-fused lysostaphin, which offers enhanced stability, low immunogenicity and sustained release via injection. It safely and completely eradicates in vivo MRSA biofilm infections, outperforming lysostaphin and vancomycin.

## Introduction

Antimicrobial resistance (AMR) stands as a critical hurdle in managing staphylococcal infections, with methicillin-resistant *Staphylococcus aureus* (MRSA) serving as a quintessential example of multidrug-resistant pathogens^1^. Beyond resistance to methicillin—conferred by the mecA gene that encodes an altered penicillin-binding protein—staphylococci have evolved defenses against a broad spectrum of antimicrobials. These include vancomycin (Van) resistance driven by vanA/vanB gene expression, macrolide resistance via efflux pumps or target site alterations, and tetracycline resistance mediated by ribosomal protection proteins^2–4^. Due to their substantial morbidity and mortality burdens, MRSA infections have emerged as one of the most prevalent infectious diseases globally, posing a grave threat to public health and prompting widespread concern within the international medical community.

Each year, hundreds of millions of medical implants—encompassing biomaterials and medical devices—are deployed worldwide, revolutionizing modern healthcare^5^. Yet, staphylococcal colonization of implant surfaces often triggers biofilm formation, which in turn precipitates implant-associated infections^6^. Among these pathogens, coagulase-negative staphylococci (CoNS), such as *Staphylococcus epidermidis*, are the most commonly isolated strains in clinical settings overall. However, MRSA is one of the most clinically significant microorganisms associated with such infections, especially in instances characterized by high rates of morbidity and mortality. Within biofilms, MRSA exhibits marked tolerance to both innate and adaptive immune responses, as well as conventional antibiotics^7^. The biofilm matrix acts as a physical barrier that limits antibiotic penetration, resulting in sub-inhibitory drug concentrations at the biofilm interior. These sub-inhibitory concentrations select for bacterial variants with enhanced resistance mechanisms (e.g., efflux pump upregulation, target site modification), which in turn drives the evolution of antibiotic resistance and results in persistent infections that are unresponsive to standard therapeutic approaches^8^. Regrettably, current management strategies for MRSA biofilm-related implant infections are limited to aggressive surgical debridement, prolonged systemic antibiotic regimens, and device removal—interventions that inflict significant morbidity and mortality on patients^9–11^.

Bacteriolysins, a class of bacteriolytic enzymes, have emerged as promising substitutes for antibiotics in the treatment of bacterial infections associated with biofilms^12^. As a prototypical bacteriolysin, lysostaphin (Lst)—naturally produced by the Gram-positive bacterium *Staphylococcus simulans*—specifically hydrolyzes the pentaglycine cross-bridges within the peptidoglycan layer of staphylococcal cell walls, triggering bacterial lysis and biofilm degradation^13,14^. Furthermore, Lst also demonstrates high specificity in eradicating multidrug-resistant staphylococci, including MRSA and methicillin-resistant *Staphylococcus epidermidis* (MRSE) as well as their biofilms. Mutations in femA/femB genes encoding pentaglycine cross-bridge synthesis can reduce Lst’s efficacy, though such mutations are rare in clinical isolates^15^. Nevertheless, these mutants are completely deprived of their resistance to methicillin^16,17^ and become highly susceptible to a variety of unrelated antibiotics, including Van^18^. Additionally, they are less fit and virulent, and become easily treatable with antibiotics in vitro and in vivo^15^. Therefore, Van resistance and Lst resistance have distinct mechanisms with no known overlap, making Lst-based therapies a promising option for Van-resistant staphylococcal infections. While preclinical studies and clinical trials have yielded promising outcomes^19,20^, Lst has not been approved as a parenteral antibiotic alternative, primarily owing to unaddressed immunogenicity concerns. Indeed, Lst administration in both animal models and human subjects induces potent anti-Lst antibody responses, which impair therapeutic efficacy by neutralizing Lst’s catalytic activity via binding to its active site or allosteric regions and accelerating its clearance via antibody-mediated opsonization and phagocytosis by immune cells. These antibodies also pose risks to patient safety, including hypersensitivity reactions (e.g., urticaria, anaphylaxis) in sensitized individuals, and reduced efficacy upon repeated dosing—critical limitations for treating chronic or recurrent infections^21,22^. Moreover, Lst is plagued by inadequate stability and subpar pharmacokinetic properties, which have been thoroughly investigated in recent research^23^. In particular, Lst is highly vulnerable to proteolytic breakdown by serum proteases, exhibiting a half-life of merely a few hours in mouse serum; this short half-life may require frequent drug administration, thereby triggering the production of neutralizing antibodies.

Recent efforts to overcome these limitations have involved engineering deimmunized Lst variants via T cell epitope deletion, which permits safe, repeated dosing for treating refractory MRSA infections in animal models^24–27^. However, these variants still display inadequate stability and subpar pharmacokinetic profiles. Alternative strategies have entailed adsorbing or encapsulating Lst within implants^28–31^, entrapping it in hydrogels^32,33^, or loaded into polymeric micro/nanoparticels^34,35^ or exosomes^36^. All these strategies are designed to maintain sustained local concentrations at infection sites and reduce systemic toxicity in comparison with systemic administration. Nevertheless, achieving controlled release of Lst from these platforms to yield optimal therapeutic outcomes remains elusive, and none of these approaches address Lst’s intrinsic immunogenicity. Additionally, polyethylene glycol conjugation (PEGylation) has been employed to prolong Lst’s half-life^37^; however, this PEGylation strategy substantially diminishes its bioactivity. In summary, current Lst formulations fail to fully mitigate its core limitations, thus precluding the attainment of optimal therapeutic efficacy coupled with favorable safety profiles.

**Fig. 1:**
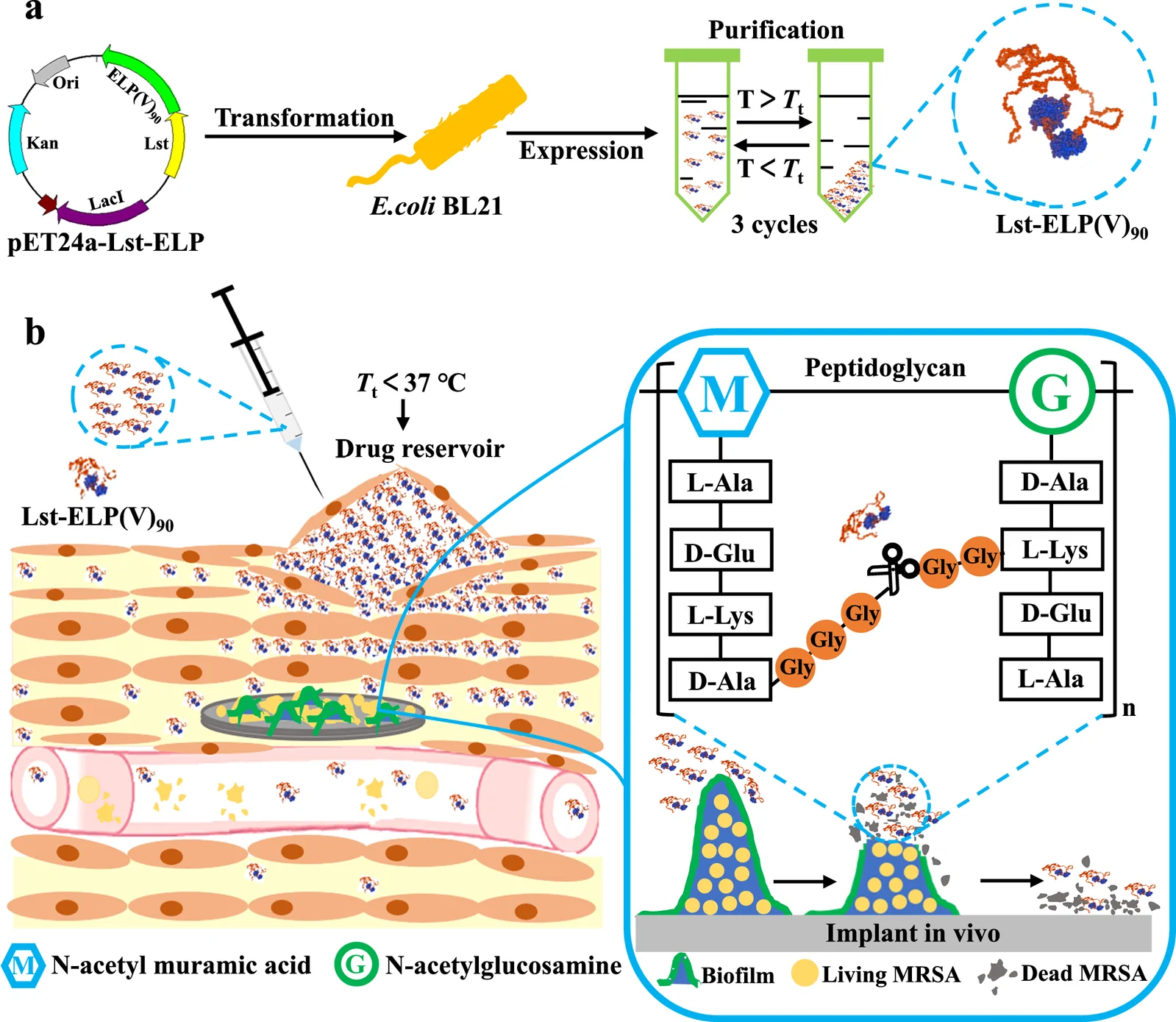
Preparation of a low-immunogenic sustained-release chimeric protein of Lst-ELP(V)_90_ for complete eradication of MRSA biofilm-associated implant infections. **a** Biosynthetic route of Lst-ELP(V)_90_ exhibiting a reversible thermosensitive phase transition behavior. **b** Complete eradication of MRSA biofilm-related implant infections using Lst-ELP(V)_90_. Upon subcutaneous injection, Lst-ELP(V)_90_ forms an in situ sustained-release depot adjacent to implants infected with MRSA biofilms, owing to its reverse thermosensitive property. The soluble, released Lst-ELP(V)_90_ diffuses to the infected site, where it efficiently lyses MRSA via specific cleavage of the pentaglycine cross-bridges within the bacterial cell wall peptidoglycan layer while also degrading extracellular polymeric substances (EPS) and thereby disrupting the MRSA biofilm. Source data are provided as a Source Data file.

Herein, we present a strategy—fusing elastin-like polypeptides (ELPs) to Lst—to address its inherent limitations and enhance its potential as an antibiotic alternative. We employed AlphaFold2 to predict the three-dimensional (3D) structure of ELP-fused Lst chimeric proteins (Lst-ELP), which facilitates characterization of their in vitro and in vivo properties and thereby accelerates the rational engineering of Lst-ELP for MRSA biofilm infection treatment. ELPs are thermosensitive polypeptides with a reversible phase transition temperature (*T*_t_), featuring non-cytotoxicity and low immunogenicity^38–41^. We hypothesized that the reverse thermos-responsiveness of an optimized ELP, ELP(V)_90_, would endow Lst-ELP(V)_90_ with the ability to form a sustained-release depot upon subcutaneous injection adjacent to MRSA-infected implants—exhibiting enhanced stability and prolonged bioactivity at infection sites compared to free Lst (Fig. 1). Furthermore, fusion with the low-immunogenic ELP(V)_90_ is expected to reduce Lst’s immunogenicity. Consequently, compared to free Lst, Lst-ELP(V)_90_ exhibits improved pharmacokinetics and optimized biodistribution, enabling complete eradication of both local and systemic MRSA infections in a murine implant infection model—an outcome not achievable with free Lst or vancomycin (Van), the first-line antibiotic for MRSA.

## Results

### AlphaFold2-assisted design of Lst-ELP(V)_90_

ELPs are synthetic biopolymers consisting of repeated Val-Pro-Gly-Xaa-Gly (VPGXG) motifs^38–41^. Based on our accumulated understanding of how the guest amino acid Xaa and the number of repeat units influence the transition temperature (*T*_t_) of ELPs^42–53^, we selected valine (V) as Xaa to design ELP(V)_30_, ELP(V)_60_, and ELP(V)_90_, which include 30, 60, and 90 VPGVG repeats, respectively. Given that the active site of Lst is distal to both the N- and C-termini^54^, we resolved to genetically fuse these ELP(V) variants to either terminus of Lst, generating Lst-ELP(V) (C-terminal fusion) or ELP(V)-Lst (N-terminal fusion). However, the yield of ELP(V)-Lst expressed in *E. coli* was remarkably low as compared to Lst-ELP(V); thus, we selected Lst-ELP(V) for subsequent in vitro and in vivo studies.

**Fig. 2:**
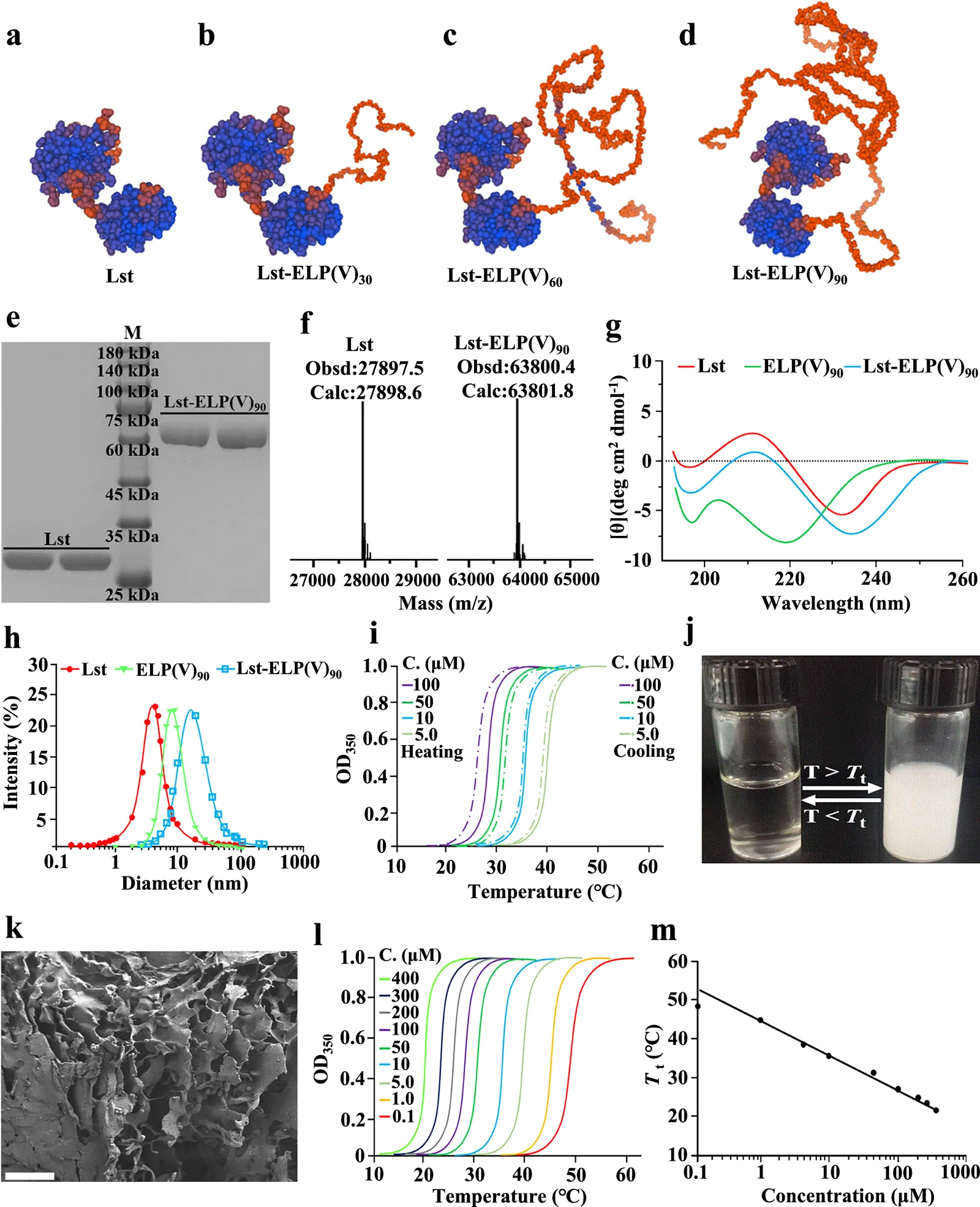
Construction and physicochemical characterization of Lst-ELP(V)_90_. **a–d** Alphafold2 prediction of 3D structures for Lst, Lst-ELP(V)_30_, Lst-ELP(V)_60_ and Lst-ELP(V)_90_. **e** Analysis of Lst and Lst-ELP(V)_90_ by Gel Electrophoresis. SDS-PAGE analysis of Lst and Lst-ELP(V)_90_ was performed in triplicate, yielding consistent results. **f** Mass spectrometry analysis for Lst and Lst-ELP(V)_90_. “Calc” represents the theoretical molecular weight. “Obsd” represents the detected value. **g** Analysis for Lst, ELP(V)_90_ and Lst-ELP(V)_90_ by CD. **h** DLS analysis for Lst, ELP(V)_90_ and Lst-ELP(V)_90_ at 0.5 µM in PBS. **i** Heating and cooling profiles of Lst-ELP(V)_90_ in PBS. OD_350_ represents optical density at 350 nm. “C.” represents concentration. **j** Photograph of the solution of Lst-ELP(V)_90_ at 50 µM in PBS at a temperature above or below *T*_t_. **k** SEM images of the precipitate of Lst-ELP(V)_90_. SEM examination of Lst-ELP(V)_90_ was conducted on four parallel samples prepared on copper grids, yielding reproducible results. Scale bars, 20 μm. **l** Turbidity versus temperature of Lst-ELP(V)_90_ at varying concentrations. **m** *T*_t_ versus concentration relationship of Lst-ELP(V)_90_, quantified from the data presented in this figure (**l**). Source data are provided as a Source Data file.

In this study, we employed AlphaFold2 to predict the 3D structure of Lst-ELP(V) (Fig. 2a–d). In the soluble state, the 3D structure of Lst within Lst-ELP(V) is well preserved compared with that of free Lst, indicating that tethering ELP(V) to the C-terminus does not perturb Lst folding. AlphaFold2 further predicted that Lst is dynamically surrounded by the tethered ELP(V) chain, which adopts a random coil conformation. Moreover, the dynamic coverage of Lst by ELP(V) increases with the increasing chain length of ELP(V). We reasoned that this dynamic coverage can not only enhance Lst stability and pharmacokinetics—by protecting Lst and increasing its size—but also reduce Lst’s immunogenicity by preventing its recognition and uptake by immune cells. Therefore, we determined to select Lst-ELP(V)_90_ for further investigations.

### Physicochemical characterization of Lst-ELP(V)_90_

Lst-ELP(V)_90_ was overexpressed in *Escherichia coli* and purified utilizing the reversible thermoresponsive phase transition property of ELP(V)_90_, showing high purity (Fig. 2e). Further characterization by mass spectrometry showed that the observed molecular weight of Lst-ELP(V)_90_ (63800.4 Da) was identical to the theoretical value (63801.8 Da) (Fig. 2f). Circular dichroism (CD) analysis of its secondary structure revealed similarities to Lst rather than ELP(V)_90_, indicating that the secondary structure of Lst is well retained after fusion with ELP(V)_90_ (Fig. 2g). As determined by dynamic light scattering (DLS), the hydrodynamic diameter of Lst-ELP(V)_90_ (16.5 nm) exceeded those of Lst (4.8 nm) and ELP(V)_90_ (8.1 nm) (Fig. 2h). We reasoned that this increased size would reduce renal clearance of Lst, thereby improving its pharmacokinetics. We further investigated the thermosensitive phase transition behavior of Lst-ELP(V)_90_ via turbidity measurements of sample solutions at different concentrations. A rapid turbidity increase was observed when the solution temperature reached *T*_t_, indicating a sharp phase transition (Fig. 2i). This thermally induced phase transition exhibited reversible characteristics accompanied by mild hysteresis behavior, which can be clearly observed in Fig. 2j. Scanning electron microscopy (SEM) characterization demonstrated that the condensed aggregates formed by Lst-ELP(V)_90_ possessed a porous layered structural morphology (Fig. 2k). Additionally, the *T*_t_ of Lst-ELP(V)_90_ was 30.5 ± 0.8 °C at 50 μM, 32.2 ± 0.6 °C at 25 μM, and 35.2 ± 0.5 °C at 10 μM (Fig. 2l, m), showing an inverse correlation between concentration and *T*_t_ (Fig. 2l, m), meaning that high-concentration Lst-ELP(V)_90_ may remain soluble at low temperatures below *T*_t_ but precipitates at 37 °C (above *T*_t_). Conversely, even at 37 °C, low-concentration Lst-ELP(V)_90_ remains soluble because its *T*_t_ exceeds 37 °C. Based on this concentration dependence of *T*_t_, we reasoned that high-concentration Lst-ELP(V)_90_ would precipitate upon subcutaneous injection, forming a sustained-release depot with long-acting properties.

### High bactericidal activity of Lst-ELP(V)_90_

**Fig. 3:**
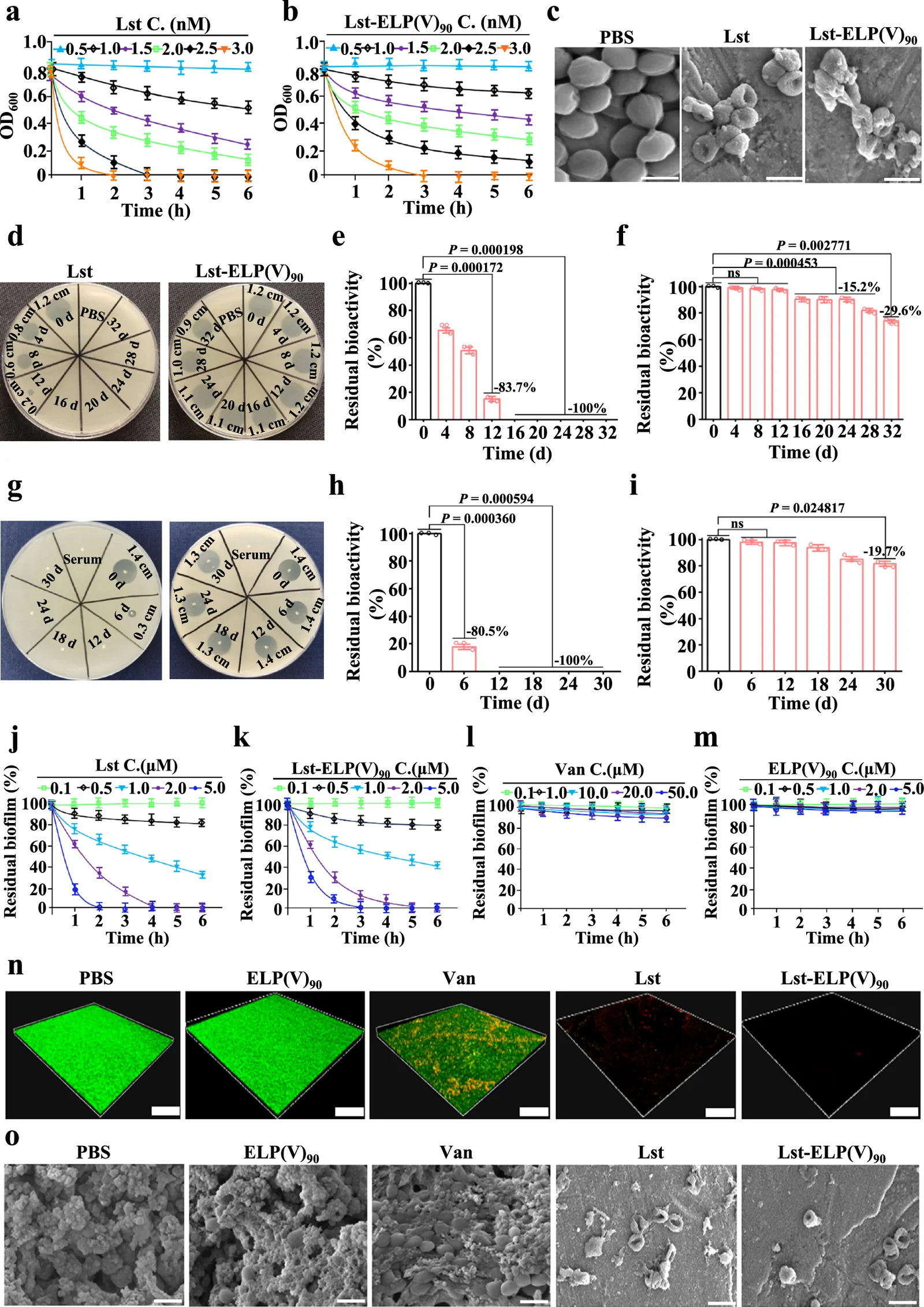
High antibacterial and biofilm-clearing activity and enhanced stability of Lst-ELP(V)_90_. **a**, **b** Turbidity-time curves of planktonic MRSA treated with Lst and Lst-ELP(V)_90_ at given concentrations. OD_600_ denotes optical density at 600 nm. All experiments were performed in at least three independent biological replicates in vitro. In this figure (**a**, **b**), *n* denotes the OD600 absorbance values obtained from three technical replicates within a 96-well plate. **c** SEM images of planktonic MRSA treated with Lst and Lst-ELP(V)_90_ at 3 nM at 37 °C for 2 h. The experiment was performed in triplicate, yielding highly consistent results. Scale bars, 1 μm. **d** Evaluation of the antibacterial activity of Lst and Lst-ELP(V)_90_ at 5 μM in PBS post-incubation at 37 °C for various times by means of the zone of inhibition assay. **e**, **f** Quantification of the residual activity of Lst and Lst-ELP(V)_90_ from this figure (**d**). **g** Evaluation of the antibacterial activity of Lst and Lst-ELP(V)_90_ at 50 μM in serum post-incubation at 37 °C for various times by means of the zone of inhibition assay. **h**, **i** Quantification of the residual activity of Lst and Lst-ELP(V)_90_ from this figure (**g**). In figure (**e**, **f**, **h**, **i**), *n* corresponds to the diameters of the zones of inhibition measured across three independent plates. **j–m** Biomass-time curves of MRSA biofilms treated with Lst, Lst-ELP(V)_90_, Van, and ELP(V)_90_ at given concentrations at 37 °C. In this figure (**j**–**m**), *n* represents the OD595 absorbance readings from three technical replicates per well. **n**, **o** CLSM and SEM analyses of MRSA biofilms after varying treatments with PBS, ELP(V)_90_ at 5 μM, Van at 20 μM, Lst and Lst-ELP(V)_90_ at 5 μM at 37 °C for 3 h. For microscopic imaging experiments, three parallel preparations were made for each sample. Green fluorescence indicates viable cells, while red fluorescence indicates non-viable cells. Scale bars, 50 μm for CLSM images, 1 μm for SEM images. All data are expressed as the mean ± standard deviation (SD) (*n* = 3). *P*-values are assessed by one-way analysis of variance (ANOVA) analysis; *P < 0.05, **P < 0.01, ***P < 0.001, and ****P < 0.0001, ns denotes no significance. Source data are provided as a Source Data file.

To evaluate the bioactivity of Lst-ELP(V)_90_, a MRSA suspension was treated with the fusion protein, and the optical density of the suspension was measured to monitor bacterial growth over time. The inhibitory effect on bacterial growth was dose-dependent for Lst-ELP(V)_90_, consistent with that of Lst (Fig. 3a, b). The minimum inhibitory concentration (MIC = 0.5 nM) and minimum bactericidal concentration (MBC = 3.0 nM) of Lst-ELP(V)_90_ were comparable to those of Lst (MIC = 0.5 nM, MBC = 2.5 nM), indicating that ELP(V)_90_ fusion does not significantly impair the antimicrobial activity of Lst. Notably, the MIC and MBC of Lst-ELP(V)_90_ were 700- and 350-fold lower than those of Van (MIC = 0.35 μM, MBC = 1.05 μM), respectively (Supplementary Fig. 1), demonstrating that Lst-ELP(V)_90_ exhibits superior antibacterial activity compared to Van. As expected, ELP(V)_90_ showed no antimicrobial activity (Supplementary Fig. 2).

SEM revealed that MRSA morphology was severely altered—from spherical to irregular shapes—following treatment with Lst-ELP(V)_90_ and Lst at the same concentration of 3.0 nM (Fig. 3c), or with Van at a much higher concentration of 1.5 μM (Supplementary Fig. 3a). No morphological changes were observed in MRSA treated with ELP(V)_90_ (Supplementary Fig. 3b). Together, these results confirm that Lst-ELP(V)_90_ effectively retains the antimicrobial activity of Lst and efficiently kills MRSA.

### Enhanced stability of Lst-ELP(V)_90_

To investigate the stability of Lst-ELP(V)_90_, we evaluated its retention of antibacterial activity at low concentration (soluble state) or high concentration (precipitated state) in PBS or serum following incubation at 37 °C for specified durations. In the soluble state, Lst-ELP(V)_90_ retained 75% in PBS and 18.2% in serum of its initial activity after 32 d and 3 h, respectively—whereas Lst lost all its initial activity under the same conditions (Supplementary Fig. 4 and Fig. 3d–f). This indicates that soluble Lst-ELP(V)_90_ exhibits significantly greater stability than free Lst in both PBS and serum. Furthermore, in the precipitated state, Lst-ELP(V)_90_ retained 98.8% of its initial activity in serum after 12 d, compared to 0% for Lst (Fig. 3g–i), suggesting that the fusion protein would retain high bioactivity following subcutaneous injection to form a sustained-release depot. We further evaluated Lst-ELP(V)_90_ stability in mouse liver homogenate (37 °C), finding that it retained 82.1% of its initial activity after 30 d, compared to 17.9% for free Lst after 6 d (Supplementary Fig. 5). This result verifies that the prepared sample achieves superior stability under physiological tissue-mimicking conditions. Additionally, we used CD spectroscopy to directly measure protein unfolding over time, confirming that Lst-ELP(V)_90_ well retains its secondary structure (almost 100% of initial α-helix content) after 7 d in PBS at 37 °C, whereas Lst loses most of its α-helix content after 7 d (Supplementary Fig. 6). Collectively, these results demonstrate that ELP(V)_90_ fusion enhances Lst’s stability, which is attributed to the dynamic protection provided by the tethered ELP(V)_90_ chain. Such structural stabilization would be conducive to optimizing the in vivo pharmacokinetic behavior of Lst.

### High biofilm-clearing activity of Lst-ELP(V)_90_

Biofilm formation by bacteria represents a crucial characteristic of infections associated with implanted medical devices^5^. Biofilms protect bacteria from host immune defenses and the bactericidal effects of antibiotics, making them particularly challenging to eradicate^7,8^. Lst is capable of not only eradicating staphylococcal bacteria but also degrading the biofilms produced by staphylococci. Given that Lst-ELP(V)_90_ retains high anti-MRSA activity comparable to Lst, we reasoned that it can also preserve Lst’s anti-biofilm activity. Notably, in all in vitro biofilm experiments, MRSA biofilms were allowed to form for 72 h before treatment with Lst-ELP(V)_90_, Lst, Van, or ELP(V)_90_. Crystal violet staining of MRSA biofilms demonstrated that both Lst and Lst-ELP(V)_90_ exhibited dose-dependent biofilm elimination (Supplementary Fig. 7 and Fig. 3j, k). The minimum biofilm inhibitory concentration (MBIC = 0.1 μM) and minimum biofilm eradication concentration (MBEC = 4.0 μM) of Lst-ELP(V)₉₀ were comparable to those of Lst (MBIC = 0.1 μM, MBEC = 4.0 μM) (Supplementary Fig. 8), indicating that ELP(V)_90_ fusion does not significantly compromise the biofilm elimination efficiency of Lst. In contrast, Van did not show any significant biofilm-clearing activity even at a high concentration of 100 μM, indicating that Van is much less effective than Lst analogs to clear MRSA biofilms (Fig. 3l and Supplementary Fig. 8). Consistent with our anticipation, ELP(V)_90_ failed to exert any elimination effect against MRSA biofilms. (Fig. 3m).

To further evaluate the elimination efficacy of bacteria embedded in biofilms, live/dead fluorescent staining was adopted in this study. Confocal laser scanning microscopy (CLSM) observations confirmed that both free Lst and Lst-ELP(V)_90_ conjugates could effectively remove MRSA biofilm structures (Fig. 3n). In contrast, Van was far less effective, and ELP(V)_90_ had no biofilm-eliminating activity. SEM imaging confirmed complete biofilm removal on titanium discs treated with Lst-ELP(V)_90_ (Fig. 3o). These results demonstrate that Lst-ELP(V)_90_ is effective against biofilms formed on implant materials, closely simulating clinical conditions. Collectively, these results indicate that ELP(V)_90_ fusion does not significantly impair Lst’s anti-biofilm activity.

We serially passaged MRSA in the presence of sub-MIC concentrations of Lst-ELP(V)_90_, free Lst, and Van for 30 d that correspond to 150 generations. Both Lst-ELP(V)_90_ and Lst did not induce any resistance, as indicated by no MIC increase (Supplementary Fig. 9). By stark contrast, the MIC of Van started to increase on day 15 (75 generations) and increased by twofold on day 30, indicating the induction of resistance to Van.

We tested Lst-ELP(V)_90_ against MRSA in stationary phase (10⁹ CFU/mL, starvation for 48 h) and persister cells (induced by 5 × MIC of Van for 24 h). Lst-ELP(V)_90_ killed 99.8% of stationary-phase MRSA at 3 nM (MBC) and 96.4% of persister cells at 3 nM, whereas Van (3.5 μM) killed only 7.2% of stationary-phase cells and 3.4% of persister cells (Supplementary Fig. 10). This is attributed to Lst’s catalytic mechanism, which is independent of bacterial metabolic state—unlike Van, which requires active bacterial growth to exert its effects.

We conducted antibacterial tests of Lst-ELP(V)_90_ against a clinical isolate of Van-resistant *Staphylococcus aureus* (VRSA), showing MIC = 0.5 nM and MBC = 3.0 nM—comparable to its activity against MRSA (Supplementary Fig. 11). These data confirm that Lst-ELP(V)_90_ has very low potential for resistance induction and is effective against Van-resistant bacteria.

Furthermore, our experimental results showed that the Lst-ELP(V)_90_ fusion protein exhibited similar anti-MRSE activity (MIC = 1.0 nM, MBC = 3.0 nM) and biofilm-removing effect against MRSE compared with MRSA (Supplementary Fig. 12), indicating that Lst-ELP(V)_90_ is applicable to both MRSA and MRSE.

### Increased maximum tolerated dose (MTD) of Lst-ELP(V)_90_

To conduct in vivo studies, we initially determined the MTD of Lst-ELP(V)_90_ in mice via dose escalation (Supplementary Fig. 13). The MTDs of Lst-ELP(V)_90_ and Lst were measured as 128 and 32 μmol/kg, respectively, indicating that Lst-ELP(V)_90_ is significantly tolerable relative to Lst. Since ELP(V)_90_ exhibited a safety profile that precluded MTD determination, we elected to use the MTD of Lst-ELP(V)_90_ as the experimental dose for ELP(V)_90_ in subsequent experiments.

### Sustained release of Lst-ELP(V)_90_

**Fig. 4:**
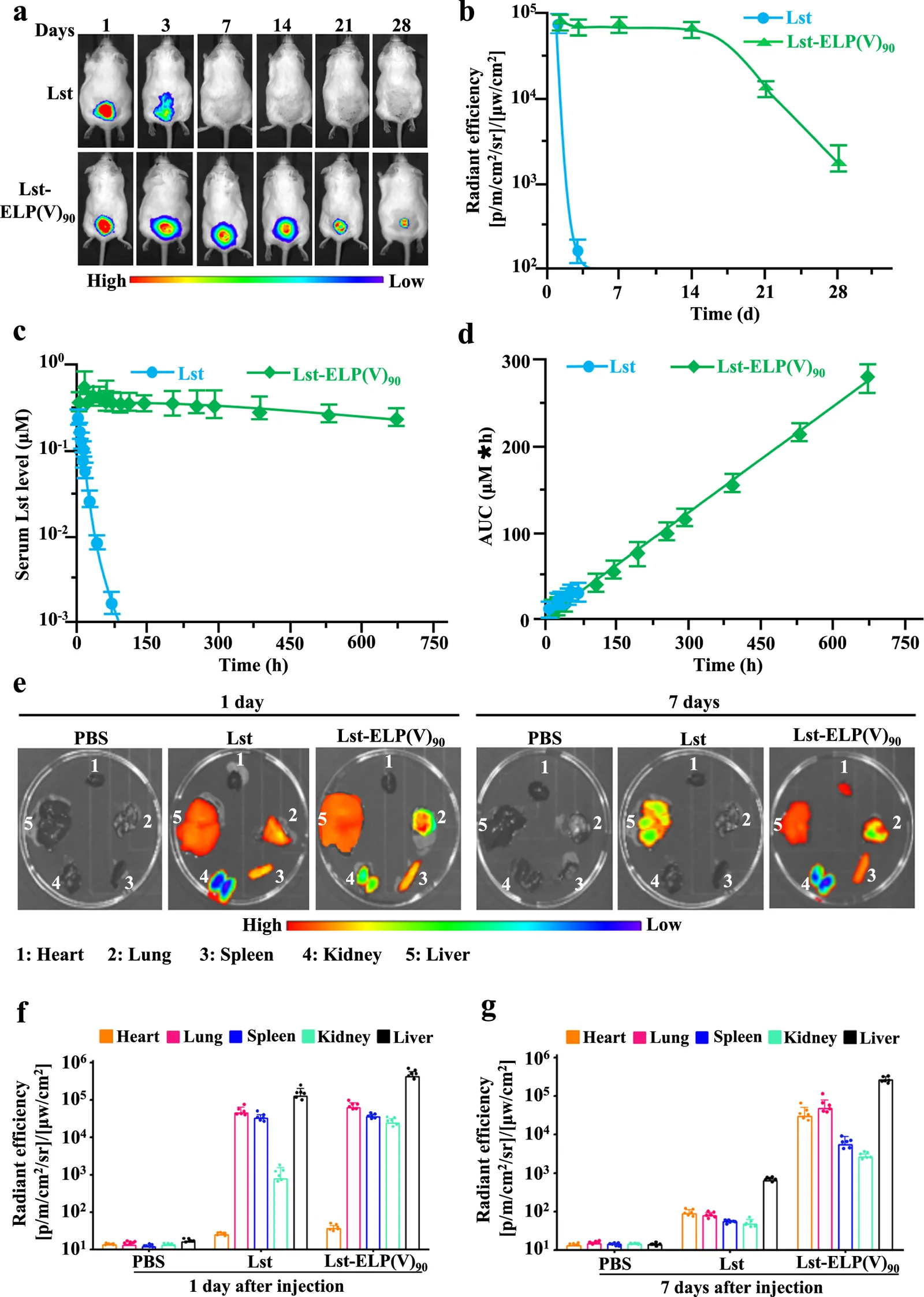
Sustained release, enhanced pharmacokinetics and biodistribution profile of Lst-ELP(V)_90_. **a** In vivo fluorescence imaging of Cy5-labeled Lst and Lst-ELP(V)_90_ at subcutaneous injection locations (*n* = 6). The color bar indicates fluorescence intensity, with red signifying high intensity and blue signifying low intensity. **b** Fluorescence evolution of Cy5-labeled Lst and Lst-ELP(V)_90_, quantified from this figure (**a**). **c** Serum Lst concentration as a function of time after subcutaneous injection of Cy5-labeled Lst and Lst-ELP(V)_90_ (*n* = 6). The experiment was repeated three times using independently prepared samples, and the results showed negligible inter-batch variability. **d** AUC versus time (*n* = 6), calculated from the data in this figure (**c**). **e** Fluorescence imaging of major organs at 1 day and 7 days after subcutaneous injection of Cy5-labeled Lst and Lst-ELP(V)_90_ (*n* = 6), respectively. **f**, **g** Fluorescence quantification of Cy5-labeled Lst and Lst-ELP(V)_90_ in the major organs (*n* = 6), calculated from the data in this figure (**e**). Three independent experimental runs were performed, yielding highly consistent results across all batches. In this figure (**b**–**d**, **f**, **g**), data are presented as mean ± SD and Error bars represent standard deviation (SD). Source data are provided as a Source Data file.

To investigate the in vivo sustained-release behavior of Lst-ELP(V)_90_, Cy5-labeled Lst-ELP(V)_90_ at its MTD was administered via subcutaneous injection and monitored using in vivo fluorescence imaging (Fig. 4a, b). Immediately after subcutaneous injection, Cy5-labeled Lst-ELP(V)_90_ formed a sustained-release depot exhibiting strong fluorescence. The fluorescence gradually diffused from the depot and decreased in intensity, with this sustained-release profile persisting for over 28 d. By contrast, following subcutaneous injection of Cy5-labeled Lst, the fluorescence diffused rapidly and was no longer detectable at the injection site within one week. The long-term sustained release of Lst-ELP(V)_90_ is thought to result from its reverse concentration-dependent thermosensitivity.

### Advanced pharmacokinetics of Lst-ELP(V)_90_

We hypothesized that the long-term sustained release of Lst-ELP(V)_90_ would improve its pharmacokinetic profile compared to free Lst. Following subcutaneous injection, the serum fluorescence of Cy5-labeled Lst-ELP(V)_90_ increased rapidly to a maximum at 17.6 h, remained relatively constant during the subsequent 16 d, and eventually declined (Fig. 4c). In contrast, Cy5-labeled Lst exhibited a rapid rise in serum fluorescence immediately after injection, followed by a rapid subsequent decrease—consistent with the fast clearance of free Lst from the bloodstream. Using these pharmacokinetic data, we determined pharmacokinetic parameters via a two-compartment model (Supplementary Table 1). Notably, the terminal half-life of Cy5-labeled Lst-ELP(V)_90_ was determined to be 487.6 h, representing a 115.8-fold increase compared with that of Cy5-labeled Lst, which was 4.2 h. The area under the curve (AUC) of Cy5-labeled Lst-ELP(V)_90_ exhibited a linear increase over time, indicating that Lst-ELP(V)_90_ follows zero-order release kinetics (Fig. 4d). Additionally, the AUC of Cy5-labeled Lst-ELP(V)₉₀ was 66.9-fold greater than that of Cy5-labeled Lst, demonstrating a marked improvement in the bioavailability of Lst-ELP(V)₉₀ relative to free Lst. Collectively, these results demonstrate that fusion with ELP(V)_90_ significantly enhances the pharmacokinetic properties of Lst.

### Bettered biodistribution of Lst-ELP(V)_90_

We investigated the distribution of Cy5-labeled Lst-ELP(V)_90_ in major organs via ex vivo fluorescence imaging, with organs collected at 1 and 7 d post-injection. On day 1, fluorescence intensity in all major organs was detected both in the Cy5-labeled Lst and the Cy5-labeled Lst-ELP(V)_90_ group (Fig. 4e–g), and no obvious differences were observed in fluorescence signals between the two experimental groups. In contrast, on day 7, fluorescence intensity in all major organs was substantially stronger in the Cy5-labeled Lst-ELP(V)_90_ group compared to the Cy5-labeled Lst group. Combined with the data from Fig. 4a, b, these results indicate that, relative to free Lst, Lst-ELP(V)_90_ can persist at the injection site near infected implants and diffuse to major organs—thereby maintaining high concentrations over an extended period. This distribution profile is attributed to the sustained release property and improved pharmacokinetic profile of Lst-ELP(V)_90_ compared to free Lst.

### Diminished immunogenicity of Lst-ELP(V)_90_

**Fig. 5:**
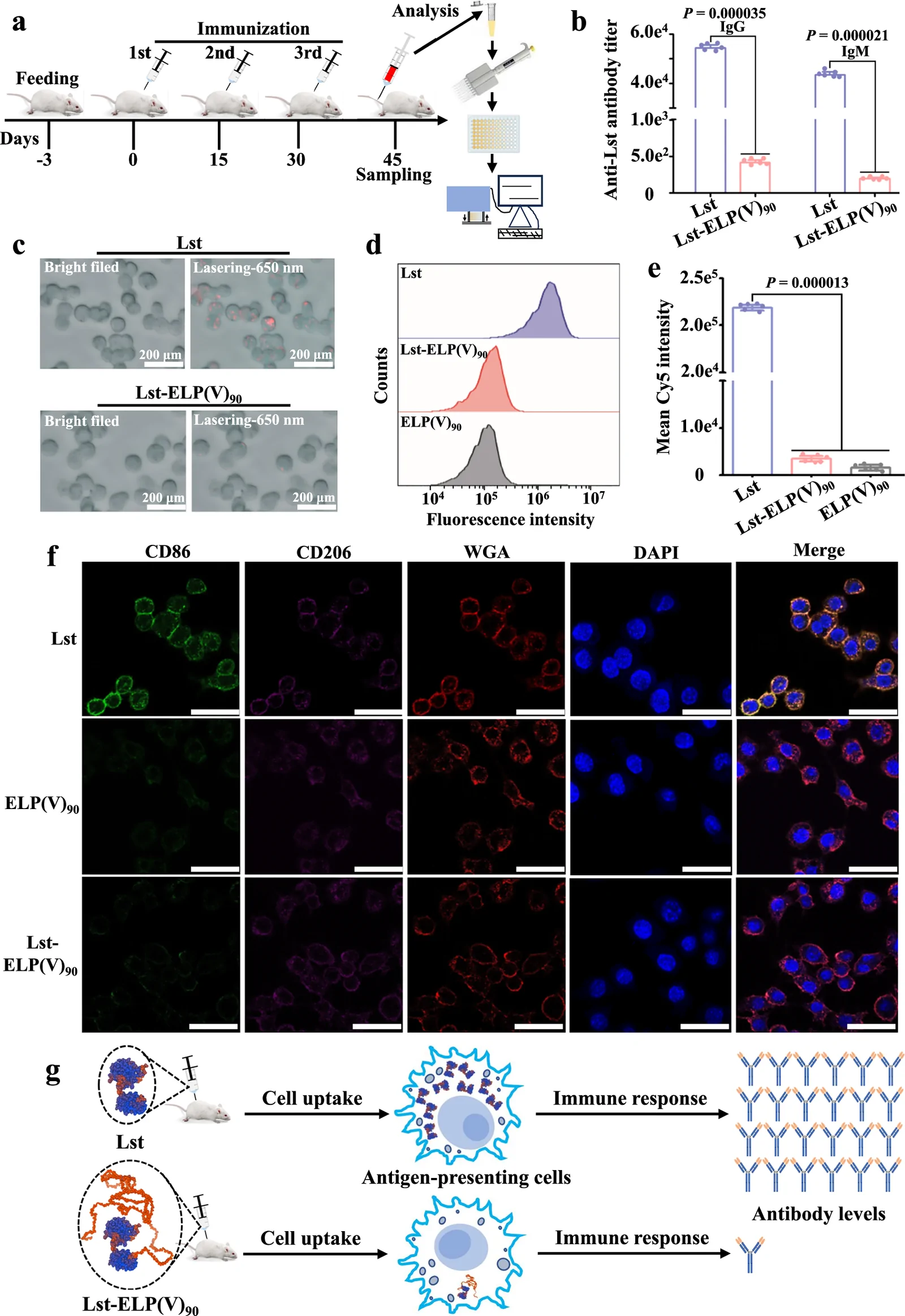
Reduced immunogenicity of Lst-ELP(V)_90_ over Lst. **a** Experimental procedures of evaluating the immunogenicity of Lst-ELP(V)_90_ in mice. **b** Anti-Lst IgG and IgM titers in serums of mice immunized with Lst or Lst-ELP(V)_90_. **c** CLSM observation of the phagocytosis of Cy5-labeled Lst and Lst-ELP(V)_90_ by macrophages. (I) and (III) both represents the bright field, (II) and (IV) both represents the lasering field under 650 nm. Scale bars, 200 μm. **d** Flow cytometry analysis of the phagocytosis of Cy5-labeled Lst, ELP(V)_90_ and Lst-ELP(V)_90_ by macrophages. **e** Fluorescence intensity quantification of this figure (**d**). **f** CLSM observation of macrophage activation by Lst, ELP(V)_90_, and Lst-ELP(V)_90_. **g** Proposed mechanism of Lst’s immunogenicity reduction by ELP(V)_90_ fusion. The data are presented as the mean ± SD (*n* = 6). The statistical significance was analyzed by ANOVA (one-way, Tukey’s multiple comparisons test). *P*-value: ****P < 0.0001, ns no significance. Source data are provided as a Source Data file.

With respect to in vivo application scenarios, the reduction of Lst’s immunogenicity is of great necessity. Therefore, we evaluated the immunogenicity of Lst-ELP(V)_90_ via multiple immunizations of mice with this Lst analog (Fig. 5a). The titers of anti-Lst IgG and IgM in the Lst-ELP(V)_90_ group were 130.6- and 221.2-fold lower than those in the Lst group (Fig. 5b), demonstrating that fusion with ELP(V)_90_ significantly diminishes the immunogenicity of Lst. CLSM revealed that the phagocytosis of Lst-ELP(V)_90_ and ELP(V)_90_ by antigen-presenting cells (APCs, herein macrophages) was substantially lower than that of Lst (Fig. 5c and Supplementary Fig. 14). Furthermore, flow cytometry analysis showed that the cellular uptake of Lst was 59.5-fold higher than that of Lst-ELP(V)_90_, which is attributable to the extremely low uptake of ELP(V)_90_ by macrophages (Fig. 5d, e and Supplementary Fig. 15).

CLSM further indicated that CD86 (a maturation biomarker of M1 macrophages, associated with pro-inflammatory responses) was significantly more highly upregulated in the Lst group compared with the ELP(V)_90_ and Lst-ELP(V)_90_ groups; however, no significant upregulation of CD206—a marker associated with M2 macrophage polarization—was detected in the Lst-ELP(V)_90_ group when compared with the Lst group. (Fig. 5f). This finding suggests that Lst-ELP(V)_90_ remarkably reduces pro-inflammatory activation, contributing to its significant mitigation of endocytosis by macrophages as compared to Lst. Collectively, these results underlie the reduced immunogenicity of Lst-ELP(V)_90_ compared with Lst (Fig. 5g).

### Improved biosafety of Lst-ELP(V)_90_

**Fig. 6:**
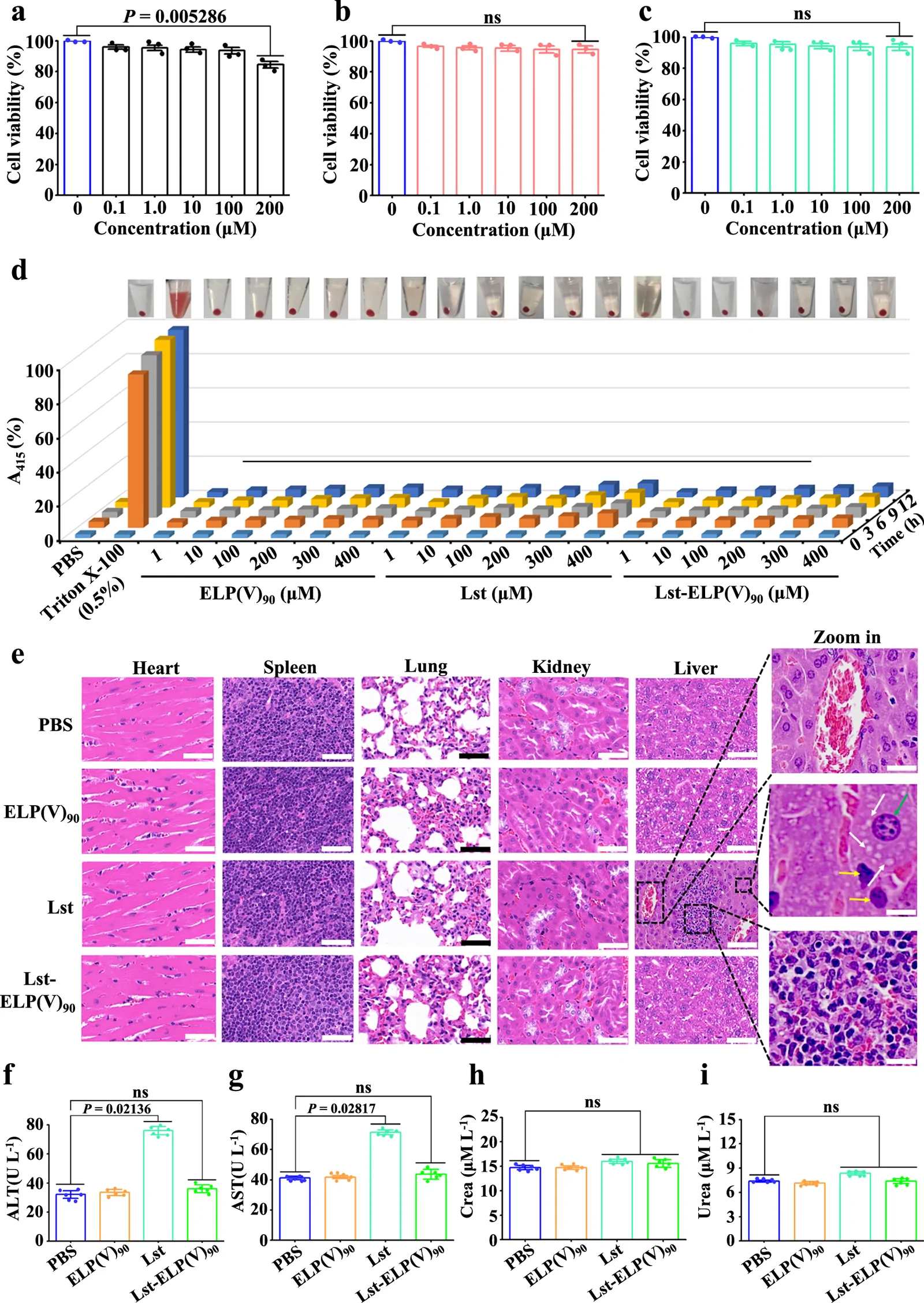
Biosafety evaluation of Lst-ELP(V)_90_. **a–c** Cytotoxicity of Lst, ELP(V)_90_ and Lst-ELP(V)_90_ at different concentrations to MC3T3-E1 cells. Error bars present the mean ± SD of three independent biological replicates; *P*-values are assessed by one-way analysis of variance (ANOVA) analysis; *P < 0.05, ns denotes no significance. **d** Hemolytic assay of ELP(V)_90_, Lst, and Lst-ELP(V)_90_ at various concentrations, with Triton ×-100 served as the positive control. **e** H&E-stained tissue sections of key organs (liver, heart, lung, spleen, and kidney) obtained from PBS, ELP(V)_90_, Lst and Lst-ELP(V)_90_ groups on day 28 post-treatment. Biosafety experiments were repeated at least three times. Scale bars in the left five columns apply to all panels except the Lst liver group (200 μm); all other panels use 100 μm. Scale bars for the enlarged images from top to bottom correspond to 50, 10, and 10 μm, respectively. **f–i** Biochemical and hematological analyses of whole blood collected for biosafety evaluation. The indicators of ALT, AST, Crea, and Urea levels were employed to evaluate liver and kidney damage, respectively. *n* indicates the number of mice (six biological replicates) used for blood biochemistry analyses in this figure (**f**–**i**). The data are presented as the mean ± SD (*n* = 6); *P*-values are assessed by one-way analysis of variance (ANOVA) analysis; **P < 0.01, ns denotes no significance. Source data are provided as a Source Data file.

At first, we evaluated the cytocompatibility of Lst-ELP(V)_90_ by CCK-8 assay. No significant cytotoxicity of Lst-ELP(V)_90_ and ELP(V)_90_ to mouse embryonic osteoblasts (MC3T3-E1) was observed even at high concentrations, whereas Lst exhibited some cytotoxicity at 200 μM (Fig. 6a–c). Moreover, even at high dosages, Lst-ELP(V)_90_, free Lst and ELP(V)_90_ triggered negligible hemolytic effects relative to the positive control Triton ×-100 (Fig. 6d). Collectively, these findings demonstrate that fusion with ELP(V)_90_ is able to enhance the cellular compatibility of Lst.

To assess the in vivo biosafety of Lst-ELP(V)_90_, mice were subcutaneously administered the fusion protein at its MTD, with comprehensive evaluations conducted on day 28 post-injection. Hematoxylin-eosin (H&E) staining of major visceral tissues including heart, liver, spleen, lung and kidney showed no obvious pathological lesions in mice receiving Lst-ELP(V)_90_ or ELP(V)_90_ treatment, in comparison with the PBS blank control group (Fig. 6e). In stark contrast, the Lst treatment group exhibited evident liver damage, characterized by hepatic vasodilation and congestion, hepatocellular nuclear fragmentation and pyknosis, cytoplasmic vacuolation (consistent with steatosis), and extensive neutrophil infiltration. Collectively, these histopathological findings serve to confirm the inherent hepatotoxic potential of Lst.

Further blood biochemistry analyses also verified hepatic damage in mice administrated with Lst. Noticeably increased levels of aspartate aminotransferase (AST) and alanine aminotransferase (ALT), two core indicators reflecting liver cell impairment, were detected in this group relative to the PBS control group (Fig. 6f–i). Conversely, measurements of AST, ALT, urea, and creatinine (Crea) demonstrated that all indices remained within the normal physiological range in mice receiving Lst-ELP(V)_90_ or ELP(V)_90_. Additionally, no significant perturbations in the hematological system were observed in the fusion protein or ELP(V)_90_ groups (Supplementary Fig. 16).

H&E staining of injection site tissues showed no significant neutrophil infiltration or tissue damage in the Lst and Lst-ELP(V)_90_ groups, compared to those in healthy tissue (Supplementary Fig. 17a). Additionally, levels of local inflammatory cytokines (TNF-α, IL-6) at the injection site were comparable to the PBS group (Supplementary Fig. 17b). These data confirm that the depot does not cause significant local accumulation or inflammatory responses.

In conclusion, Lst-ELP(V)_90_ and ELP(V)_90_ exhibit favorable systemic safety profiles in mice when administered at the MTD via subcutaneous injection, effectively mitigating the hepatotoxicity associated with Lst therapy.

### In vivo eradication of MRSA biofilms on implants by Lst-ELP(V)_90_

**Fig. 7:**
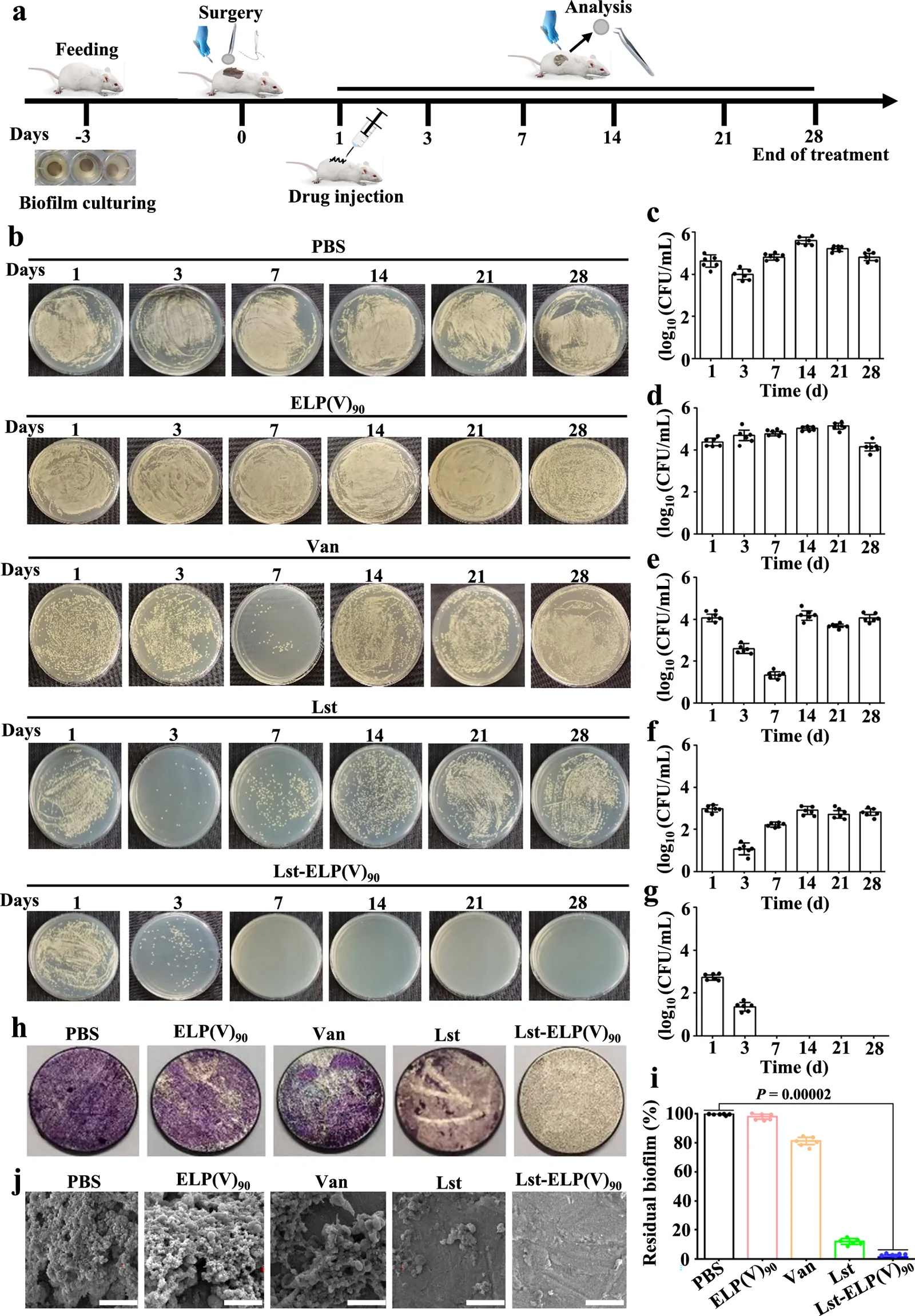
In vivo eradication of MRSA biofilms on implants after a single subcutaneous injection of Lst-ELP(V)_90_. **a** Experimental protocols for the treatment of MRSA biofilm-associated implant infections in mice. **b** Measurement of the bacterial contents of titanium alloy sheets after varying treatments by the colony formation assay. **c–g** CFU as a function of time, quantified from the data in this figure (**b**). Error bars present the mean ± SD (*n* = 6). **h** Crystal violet staining assay of titanium implants at 28 days after various treatments. **i** Relative biofilm biomass on day 28 after varying treatments, quantified from the data in this figure (**h**). The data are presented as the mean ± SD (*n* = 6). *P*-values are assessed by one-way analysis of variance (ANOVA) analysis; ****P < 0.0001. **j** SEM images of titanium implants at 28 days after various treatments. Scale bars,10 μm. The experiment was repeated independently three times. Source data are provided as a Source Data file.

We established a mouse model of implant infection via subcutaneous implantation of MRSA biofilm-infected titanium alloy sheets to evaluate the in vivo anti-infection efficacy of Lst-ELP(V)_90_ (Fig. 7a). Titanium alloy plates were co-cultured with MRSA suspension at a concentration of 10⁹ CFU/mL for 72 h under 37 °C to construct mature biofilms. After incubation, each plate contained an initial bacterial load of 10⁶ CFU/mL. The sheets were then subcutaneously implanted in mice, and treatment was initiated on day 1 post-implantation to allow further biofilm maturation in vivo. Initially, we assessed the anti-biofilm activity of Lst-ELP(V)_90_ using the colony formation assay, which involved quantifying the bacterial load on titanium alloy sheets post-treatment (Fig. 7b–g). In the Lst-only group, CFUs decreased from 1.802 (± 0.580) × 10³ CFU/mL on day 1 to 0.011 (± 0.006) × 10³ CFU/mL on day 3—initially outperforming Lst-ELP(V)₉₀, which decreased to 0.019 (± 0.006) × 10³ CFU/mL on day 3. However, CFUs in the Lst group subsequently rebounded to 0.170 (± 0.044) × 10³ CFU/mL on day 7, whereas Lst-ELP(V)₉₀ continued to reduce CFUs to zero by day 7. This early-time-point superiority of Lst likely reflects its faster initial diffusion due to smaller molecular weight compared to Lst-ELP(V)_90_, which is initially retained in the depot. However, the sustained release of Lst-ELP(V)_90_ from the depot ensures long-term exposure to therapeutic concentrations, leading to complete biofilm eradication—an outcome not achieved by Lst, which is rapidly cleared from the infection site. We further found that Van exhibited even lower efficacy against biofilm infections than Lst. As anticipated, ELP(V)_90_ showed no therapeutic effect on MRSA biofilm infections.

These findings were further validated by crystal violet staining of MRSA biofilms on titanium alloy sheets on day 28 post-treatment (Fig. 7h). The biofilm biomass in the Lst-ELP(V)₉₀ group was nearly negligible, being 10.75-fold, 58.63 fold, 69.05-fold, and 69.19-fold lower than those in the Lst, Van, ELP(V)₉₀, and PBS groups, respectively (Fig. 7i). Furthermore, SEM revealed complete elimination of MRSA biofilms in the Lst-ELP(V)₉₀ group, a phenomenon not observed in the other treatment groups (Fig. 7j). Demonstrate that Lst-ELP(V)_90_ significantly outperforms Lst and Van in treating MRSA biofilm-associated implant infections. This superiority is attributed to the long-term sustained-release properties of Lst-ELP(V)_90_ within the drug depot formed adjacent to MRSA biofilm-infected implants.

### Elimination of MRSA in the bloodstream by Lst-ELP(V)_90_

**Fig. 8:**
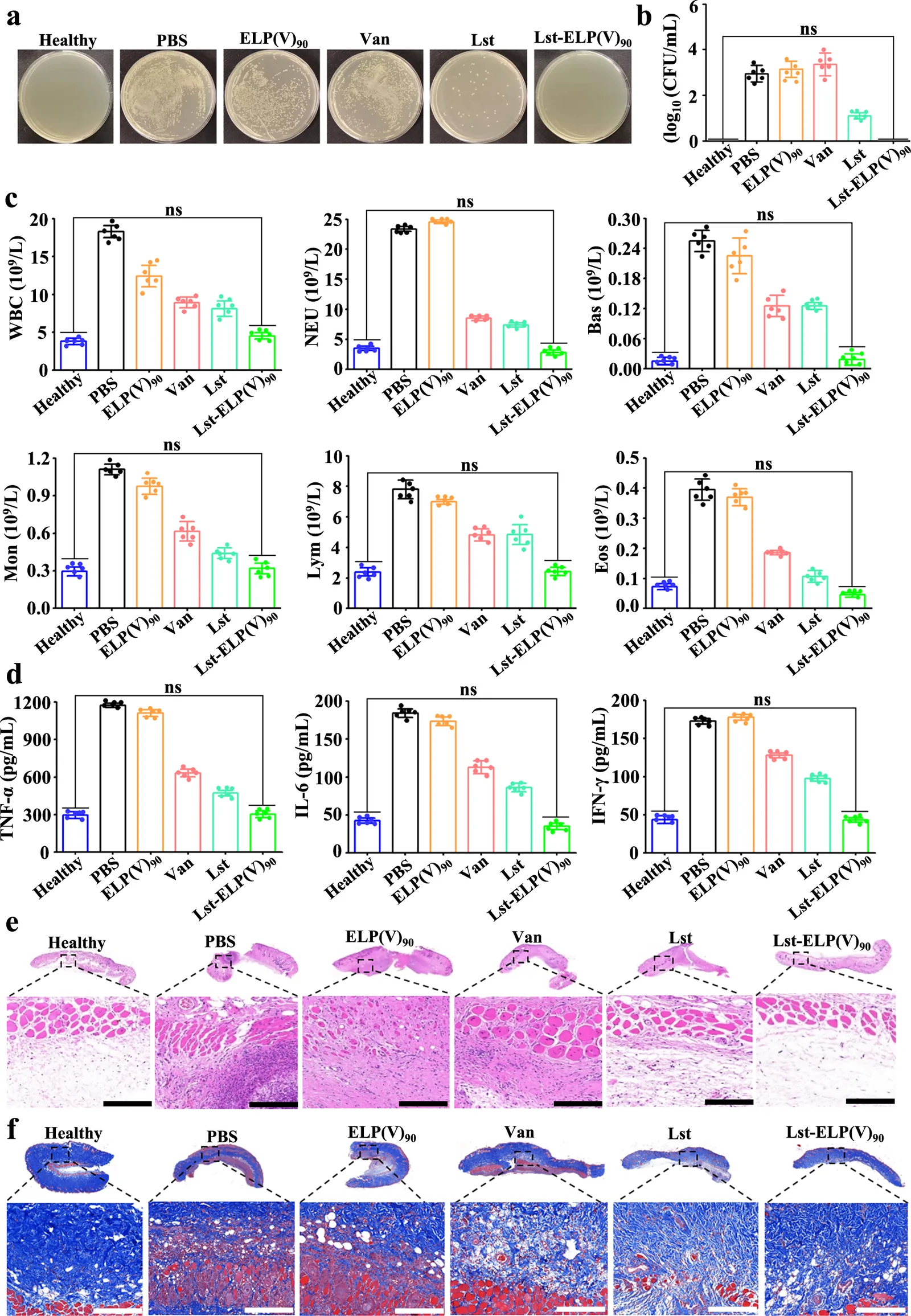
Eradication of bloodstream MRSA as well as systemic and local inflammation after a single subcutaneous injection of Lst-ELP(V)_90_. **a** Measurement of bloodstream MRSA after varying treatments by the colony formation assay. **b** CFU as a function of time, quantified from the data in this figure (**a**). **c** Levels of typical inflammatory immune cells 28 days post-different treatments. The healthy group and Lst-ELP(V)_90_ group were both highlighted with blue. **d** TNF-α, IL-6, and IFN-γ levels at 28 days after receiving various treatments. Both the healthy and Lst-ELP(V)_90_ groups were highlighted in blue. *n* indicates the number of mice (six biological replicates) used for the quantification of cell counts and inflammatory cytokines in this figure (**b**–**d**). **e**, **f** H&E and Masson-stained tissue slices of wounds from different treatment groups on day 28 post-treatment. The data are presented as the mean ± SD (*n* = 6). Three independent experimental runs were performed at separate time points. Scale bars: 100 μm for both H&E and Masson’s staining. *P*-values are assessed by one-way analysis of variance (ANOVA) analysis; **P < 0.01, ns denotes no significance. Source data are provided as a Source Data file.

MRSA can detach continuously or intermittently from biofilms and disseminate into the circulatory system, leading to bacteremia and even sepsis^7^. Therefore, we quantified the bacterial load in the blood using the colony formation assay following Lst-ELP(V)_90_ treatment in the same mice used for implant infection (Fig. 8a, b). Consistently, Lst-ELP(V)_90_ treatment completely eradicated MRSA from the bloodstream. In contrast, Lst and Van treatments failed to efficiently eradicate MRSA from the bloodstream, with Lst demonstrating greater efficacy than Van. As anticipated, ELP(V)_90_ was ineffective in eradicating MRSA in the bloodstream. These results demonstrate that Lst-ELP(V)_90_ substantially outperforms Lst in eradicating MRSA from the bloodstream, attributed to its dramatically improved pharmacokinetic profile as well as superior ability to fully eliminate MRSA biofilms compared to Lst.

### Reduction of systemic and local inflammation by Lst-ELP(V)_90_

MRSA present in the circulatory system, along with the toxins they secrete—including enterotoxins and toxic shock syndrome toxin-1 (TSST-1)—are capable of triggering an excessive and dysregulated systemic inflammatory reaction in the host organism, which may eventually lead to sepsis^8^. On this basis, we further explored the anti-inflammatory potential of Lst-ELP(V)_90_. Our experimental results demonstrated that MRSA biofilm-related implant infections led to a significant increase in the levels of inflammatory immune cells, such as white blood cells (WBC), neutrophils (NEU), basophils (Bas), monocytes (Mon), lymphocytes (Lym), and eosinophils (Eos) (Fig. 8c). This observation suggests that MRSA infection induces a severe systemic inflammatory response in the host. Treatment with Lst-ELP(V)_90_ downregulated the levels of these inflammatory immune cells to the range observed in healthy mice. In contrast, both Lst and Van showed weaker anti-inflammatory activity when compared with Lst-ELP(V)_90_. Consistent with our expectations, ELP(V)_90_ displayed almost no effectiveness in suppressing the inflammatory response. In addition, the abnormally elevated levels of inflammatory biomarkers—including tumor necrosis factor-alpha (TNF-α), interleukin-6 (IL-6), interferon-gamma (IFN-γ), C-reactive protein (CRP), and procalcitonin (PCT)—were reduced to the levels seen in healthy mice following Lst-ELP(V)_90_ treatment. This therapeutic effect was not observed in the groups treated with Lst or Van (Fig. 8d and Supplementary Fig. 18). Unsurprisingly, ELP(V)_90_ had no such effect. In summary, Lst-ELP(V)_90_ is far more effective than Lst and Van in mitigating MRSA infection-induced inflammation. This superiority is attributed to Lst-ELP(V)_90_’s ability to fully eliminate both MRSA biofilms on implants and bloodstream MRSA—an outcome not achieved by Lst or Van.

MRSA detached from biofilms on implants can disseminate into surrounding wound tissues, triggering wound infection and impairing wound healing. We therefore analyzed wound tissues following Lst-ELP(V)_90_ treatment. H&E staining revealed that neutrophil infiltration in the wound tissues was reduced to the level observed in healthy tissue—an effect not achieved by Lst or Van monotherapy (Fig. 8e). Following the treatment, Masson staining was employed to detect the presence of collagen in the wound skin tissues of different treatment groups. The results showed that the collagen content in the Lst-ELP(V)_90_ treatment group was significantly higher than that in the other treatment groups, and its level was close to that of the healthy control group. This fully demonstrates that the exogenous infection model caused by MRSA biofilm in this study was successfully cured (Fig. 8f).

### Acceleration of wound healing by Lst-ELP(V)_90_

**Fig. 9:**
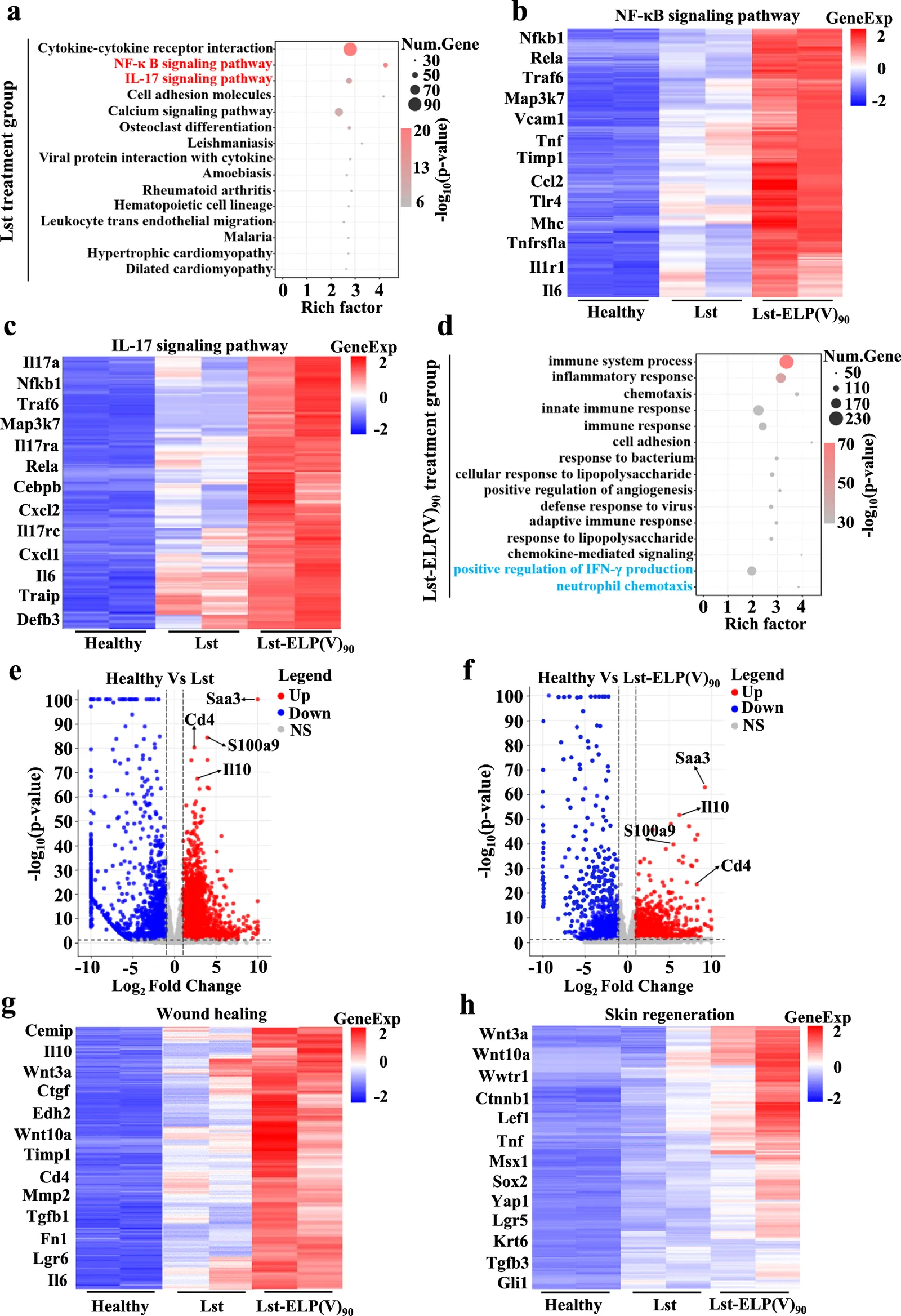
Transcriptome analysis for wounded skin tissues at 28 days following a single subcutaneous administration of Lst-ELP(V)_90_. **a** Kyoto Encyclopedia of Genes and Genomes (KEGG) enrichment analysis of the Lst treated Group. The NF-κB and IL-17 pathways, which are involved in mediating inflammatory responses, were highly expressed and highlighted in red. **b**, **c** Heat map of genes overexpressed in the NF-κB and IL-17 pathways. **d** KEGG pathway enrichment analysis for the Lst-ELP(V)_90_ treated group. The downregulation of the “positive regulation of IFN-γ production” and “neutrophil chemotaxis” pathways correlated with the resolution of infection symptoms, indicating a return to a healthy state. These two pathways were highlighted in blue. **e**, **f** Volcano plot analyses for the inflammation-related transcriptional genes of wound tissues collected at 28 d after Lst and Lst-ELP(V)_90_ treatments. **g**, **h** Heat map of the gene expression levels for wound healing and skin regeneration in the Lst and Lst-ELP(V)_90_ treatment groups. In this figure (**a**, **d**–**f**), data were analyzed by one-way ANOVA followed by Tukey’s multiple comparison test and a two-tailed *P* value < 0.05 was considered statistically significant. Source data are provided as a Source Data file.

To further evaluate the effects of Lst-ELP(V)_90_ treatment on wounded skin tissues, we performed transcriptome analysis on these tissues at 28 d post-treatment. Notably, the NF-κB and IL-17 signaling pathways were found to be highly activated in the Lst treatment group (Fig. 9a–c). Notably, the Nfkb1, Traf6 and Rela that are responsible for signal transduction in the inflammatory response were both upregulated. As a key mediator of immune defense in mice, the Il17a was widely expressed. These pathways are generally recognized as molecular alarms of inflammatory responses and reflect coordinated immune efforts to eliminate pathogens, implying a substantial MRSA burden in the wounded skin tissues. In contrast, the Lst-ELP(V)_90_ treatment group exhibited a strikingly different signaling pathway profile (Fig. 9d). The pathway mediating IFN-γ production showed minimal to no activity and was among the least active of all analyzed inflammation-related pathways. The significant downregulation of this pathway demonstrates that the inflammatory response was effectively controlled in these mice. The observed decrease in neutrophil chemotaxis—a process critical for pathogen phagocytosis—provides further evidence that MRSA infections were effectively eradicated.

We further found that in the Lst treatment group, the Saa3 and S100a9—both involved in the acute inflammatory response—were prominently upregulated, accompanied by high expression of Cd4 and Il10, genes that restrict inflammatory responses (Fig. 9e). These results revealed that pronounced inflammatory responses were triggered in the Lst-treated group. Conversely, the expression levels of Saa3 and S100a9 were markedly downregulated in the Lst-ELP(V)_90_ group relative to the Lst group, reflecting a far milder inflammatory response in the Lst-ELP(V)_90_ group (Fig. 9f). Concurrently, the inflammation-suppressive Cd4 and Il10 were also expressed at significantly lower levels in the Lst-ELP(V)_90_ group compared to the Lst group.

Collectively, our transcriptome analysis results demonstrate that Lst-ELP(V)_90_ is significantly more potent than Lst in suppressing inflammation through MRSA eradication. Thus, we hypothesized that the superior anti-inflammatory activity of Lst-ELP(V)_90_ relative to Lst would translate to enhanced wound healing and skin regeneration efficacy. Transcriptomic analysis of wound tissues further validated this speculation (Fig. 9g, h). In particular, compared with the Lst monotherapy group, the Lst-ELP(V)_90_ group displayed distinctly elevated expression of genes associated with cell proliferation, including Wnt3a and Wwtr1, as well as Wnt10a, an essential modulator governing skin tissue morphogenesis. This indicates that the synergistic effects of cell proliferation and skin remodeling on wound repair are considerably more remarkable in the Lst-ELP(V)_90_ group than in the Lst group. Our experimental results further verified this outcome, as treatment with Lst-ELP(V)_90_ was found to promote wound healing at a significantly faster rate compared with the other treatment groups involved in the study (Supplementary Fig. 19). Specifically, wounds in the Lst-ELP(V)_90_ group were nearly fully healed at 28 d post-treatment. As anticipated, the Lst, Van, ELP(V)_90_, and PBS groups had not achieved complete recovery by this time point. As a result, the body weight of mice in the Lst-ELP(V)_90_ group prominently increased with time as compared to in the other treatment groups (Supplementary Fig. 20).

The transcriptomic results of suppression of inflammatory responses and promotion of wound healing are likely a combination of bacterial clearance and direct immunomodulatory effects of Lst-ELP(V)_90_. To distinguish these, we performed in vitro experiments with lipopolysaccharide (LPS)-stimulated macrophages to mimic inflammation in the absence of bacteria. None of the treatments with Lst, Lst-ELP(V)_90_, or ELP(V)_90_ was able to downregulate the LPS-induced production of TNF-α, IL-6, and IFN-γ (Supplementary Fig. 21), which suggests that these treatments do not exert direct immunomodulatory effects on the host’s immune cells. All observed immunomodulatory activities in vivo are indirect, resulting from its peptidoglycan hydrolytic activity that eliminates MRSA, reduces pathogen-associated molecular pattern (PAMP)-driven inflammation, and enhances opsonophagocytosis. Therefore, the fusion protein exerts indirect immunomodulatory functions through the clearance of bacteria.

## Discussion

Here, we report a versatile strategy to overcome the inherent limitations of bacteriolytic enzymes by genetically fusing them with thermosensitive ELPs, using Lst as a model antibacterial enzyme. Guided by AlphaFold2 structure prediction, we rationally designed and constructed the chimeric fusion protein Lst-ELP(V)_90_, which enables single-dose subcutaneous administration adjacent to MRSA-infected implants to achieve complete eradication of biofilm-associated implant infections. Lst-ELP(V)_90_ fully preserves the potent antibacterial and antibiofilm activities of native Lst, while markedly enhancing protein stability in PBS, serum and liver homogenate at physiological temperature.

Notably, fusion with thermosensitive ELP(V)_90_ imparts reversible inverse thermoresponsiveness to Lst, characterized by a concentration-dependent *T*_t_. This unique property renders Lst-ELP(V)_90_ soluble at dilute concentrations but insoluble and self-assembling at high concentrations under physiological conditions (37 °C). Consequently, upon subcutaneous injection, concentrated Lst-ELP(V)_90_ undergoes rapid in situ phase separation to form a porous, lamellar sustained-release depot that remains stable and bioactive for at least 28 days. Mechanistically, this depot formation arises from hydrophobic interactions among ELP(V)_90_ blocks above the *T*_t_, driving reversible coacervation into a semi-solid reservoir that enables near-zero-order release kinetics. While ELP-based depot systems have been explored for long-acting delivery of protein therapeutics including interferon-α, L-asparaginase, growth hormone, trichosanthin, and anti-PD-L1 nanobodies^43–53^, this study represents the first demonstration that an ELP-Lst fusion protein can form *a* > 28-day in situ depot for the treatment of implant-associated MRSA biofilm infections. Although 28-day in vivo biosafety assessments revealed no obvious organ toxicity or histopathological damage, long-term (90-day) safety evaluations in large animal models will be performed in future studies to support clinical translation.

Compared with existing Lst engineering strategies, our ELP fusion approach offers four distinct and synergistic advantages that have not been simultaneously achieved by prior methods: (1) Full preservation of bioactivity: Lst-ELP(V)_90_ retains MIC and MBC values nearly identical to native Lst, without the activity loss observed in PEGylated Lst; (2) Dramatically reduced immunogenicity: ELP(V)_90_ shielding decreases anti-Lst antibody titers by more than two orders of magnitude and reduces macrophage uptake and pro-inflammatory activation, overcoming a major barrier to repeated clinical use; (3) Markedly enhanced stability: Lst-ELP(V)_90_ retains substantial bacteriolytic activity in serum and tissue homogenate for days to weeks, whereas free Lst is rapidly degraded; (4) Ultra-long sustained release: Single injection forms a depot that provides therapeutic concentrations for > 28 d, eliminating the need for frequent dosing and overcoming rapid systemic clearance. By contrast, PEGylation prolongs the half-life yet impairs bioactivity, while the deletion of T-cell epitopes decreases immunogenicity but fails to enhance stability or pharmacokinetic properties; and encapsulation in polymeric carriers achieves sustained release but suffers from low loading efficiency, burst release, and limited control over release kinetics. The multifunctional benefits of Lst-ELP(V)_90_, therefore, establish a paradigm for engineering bacteriolytic enzymes toward clinical translation.

A key mechanistic advantage of Lst-ELP(V)_90_ is its metabolism-independent bactericidal action, which enables efficient killing of non-dividing, stationary-phase, and persister MRSA cells—cell populations that are inherently tolerant to Van and the majority of conventional antibiotics. This feature allows Lst-ELP(V)_90_ to exert potent efficacy at nanomolar concentrations, several hundred-fold lower than Van, thereby reducing therapeutic dosage and potential off-target effects. Furthermore, Lst-ELP(V)_90_ can be efficiently produced via recombinant expression in *E. coli* and purified using ELP-mediated inverse transition cycling without chromatographic steps, supporting scalable, cost-effective manufacturing that is favorable for clinical and global health applications.

Notably, Lst-ELP(V)_90_ exhibits species-specific activity targeted toward staphylococci, including MRSA, MRSE, and VRSA, but does not act on other Gram-positive or Gram-negative pathogens. This narrow spectrum reduces disruption of commensal microbiota and lowers the risk of resistance emergence in non-target species. To broaden the antibacterial spectrum, future work will engineer modular multi-domain fusion proteins by incorporating additional antibacterial motifs (e.g., magainin, LL-37, or TCP-25) into the Lst-ELP framework to target a wider range of pathogens, including *Pseudomonas aeruginosa* and *E. coli*.

Collectively, Lst-ELP(V)_90_ combines potent bactericidal and antibiofilm activity, ultra-long sustained release, low immunogenicity, high stability, and favorable biosafety into a single injectable fusion protein. This platform enables single-dose curative therapy for MRSA biofilm-associated implant infections, addressing a major unmet clinical need that currently relies on surgical debridement, prolonged antibiotic therapy, and implant removal. Beyond implant infections, Lst-ELP(V)_90_ holds promise for treating diverse staphylococcal diseases, including sepsis, osteomyelitis, endocarditis, pneumonia, and skin and soft-tissue infections. Further preclinical evaluation in large animal models will be critical to fully validate safety, efficacy, and translational potential for clinical use.

## Methods

### Animal ethics guidance

All animal experiments were conducted in strict compliance with national laws and institutional guidelines on the care and use of laboratory animals in China. All animal experimental protocols were reviewed and approved by the Animal Ethics Committee of Peking University First Hospital (Approval ID: J2024032).

### Materials and reagents

Genes and primers were synthesized by RuiBiotech and GENEWIZ, Inc. (Suzhou, China). DNA polymerases, DNA ladders, and protein ladders were purchased from GenStar (China). DNA gel extraction kits and plasmid purification kits were obtained from TIANGEN (China). The pET24a plasmid (Cat. No. 69749-3) was purchased from Thermo Fisher Scientific (USA). The Golden Gate DNA Assembly Kit for DNA recombination was acquired from New England Biolabs (NEB, USA).

Cell Counting Kit-8 (CCK-8), SYTO 9/PI biofilm staining kit, Cy5 fluorescent dye, wheat germ agglutinin (WGA), and 4’,6-diamidino-2-phenylindole (DAPI) were purchased from Beyotime Biotechnology (China). Protein gels were obtained from CellorLab (China). DNA staining dye was purchased from Biosharp (China). Desalting columns were from Cytiva (USA), and cation exchange columns were purchased from Bio-Rad (USA). Coomassie brilliant blue staining solution for protein visualization was obtained from Leagene (China).

Primary antibodies against CD86 (Catalog No: A21198) and CD206 (Catalog No: A26785) were purchased from Abclonal Biotechnology (China). Antibody specificity was validated via manufacturer’s online validation data (https://abclonal.com.cn/) and consistent staining results reported in published literatures^55,56^. LPS was purchased from AIDISHENG (Jiangsu, China). The BCA protein quantitation kit was acquired from Beijing Solarbio Science & Technology Co., Ltd. (China). Titanium alloy discs were purchased from Xinteng Metal Technology Co., Ltd. (China). Kanamycin and vancomycin were obtained from Beijing Dingguo Biotechnology Co., Ltd. (China). Other general reagents were purchased from SINOPHARM (China).

### Bacterial strains, cells and animal feeding

*Escherichia coli* (*E. coli*) strains Top 10 and BL21(DE3) were purchased from WEIDI Biotech Co., Ltd. (China). Methicillin-resistant *Staphylococcus aureus* (MRSA, ATCC33591) and Methicillin-resistant *Staphylococcus epidermidis* (MRSE, ATCC35984) was both obtained from the American Type Culture Collection (ATCC, USA). Vancomycin-resistant *Staphylococcus aureus* (VRSA, LC1203) was obtained from Qianfoshan Hospital (Jinan, China). Mouse embryonic osteoblast cell line MC3T3-E1(Catalog No: KMCC-001) were procured from Kunmeng Biotech (Shanghai, China), murine macrophage cell line RAW264.7(Catalog No: CL-0190), and rabbit red blood cells (Catalog No: JX022-1) were procured from Procell Life Science & Technology Co., Ltd. (China). Six-week-old male Kunming mice (male) were supplied by Beijing Vital River Laboratory Animal Technology Co., Ltd. (China). Only male mice were used in this study to minimize variability associated with hormonal cycles and to ensure stable Staphylococcus aureus infection phenotypes. All experimental mice were housed in a specific pathogen-free (SPF) animal facility under controlled environmental conditions. A 12-h light/12-h dark photoperiod was implemented. Lights were switched on at 07:00 and turned off at 19:00 daily, with gradual dimmer transitions to avoid abrupt light stimulation. The room temperature was maintained at 22 ± 2 °C throughout the housing period. Indoor relative humidity was stabilized at 50 ± 10%. The strains, cells and animal used in this study are summarized in Supplementary Table 2.

### Plasmid construction, protein expression, and purification

AlphaFold2 was employed to predict the structures of a panel of fusion proteins. The encoding genes of Lst and ELP(V)₉₀ were amplified via polymerase chain reaction (PCR). The primers and plasmids utilized in this study are listed in Supplementary Tables 3 and 4, respectively. Purified DNA fragments were cloned into linearized pET24a vector using the Golden Gate assembly protocol, yielding the recombinant plasmids pET24a-Lst, pET24a-ELP(V)₉₀, pET24a-ELP(V)₉₀-Lst, and pET24a-Lst-ELP(V)₉₀. Following successful sequence verification, all constructed plasmids were transformed into the heterologous expression host *E. coli* BL21(DE3) cells.

For protein expression, a single colony harboring the recombinant plasmid was selected and inoculated into fresh Luria-Bertani (LB) liquid medium supplemented with 50 μg/mL kanamycin, followed by overnight incubation at 37 °C. The resulting seed culture was inoculated at a 1:100 (v/v) ratio into 800 mL of LB liquid medium in a 2 L Erlenmeyer flask. The bacterial culture was incubated at 37 °C with shaking until the optical density at 600 nm (OD₆₀₀) reached 0.8–1.0. Subsequently, isopropyl β-D-thiogalactoside (IPTG) was added via syringe to a final concentration of 0.5 mM to induce heterologous protein overexpression, and the culture was further incubated at 18 °C and 120 rpm for an additional 20–24 h. Finally, bacterial cells were harvested by centrifugation at 4075 × *g* and 4 °C for 15 min.

For Lst purification, the collected cell pellets were resuspended in binding buffer (50 mM Tris-HCl, 500 mM NaCl, pH 8.0) and lysed using a high-pressure homogenizer (ATS, Canada). The cell lysate was subjected to centrifugation at 18,894 × *g* and 4 °C for 15 min. The resulting supernatant was filtered through a 0.45 μm syringe filter (JinTeng) and purified by immobilized metal affinity chromatography (IMAC) equipped with a nickel-charged column, using an AKTA Purifier system (GE Healthcare, USA). The elution buffer consisted of 50 mM Tris-HCl, 500 mM NaCl, and 500 mM imidazole (pH 8.0). The purification program was set as follows: washing with binding buffer for 20 min, followed by a linear gradient elution with elution buffer increasing from 0 to 100% over 90 min. Eluted fractions were monitored in real time by measuring UV absorbance at 280 nm.

Purification of ELP(V)₉₀ and Lst-ELP(V)₉₀ fusion proteins was performed using the inverse transition cycling (ITC) method. After cell lysis, the lysate was centrifuged at 18,894 × *g* and 4 °C for 10 min to collect the supernatant. Polyethyleneimine (PEI) solution was then added to the supernatant to a final concentration of 1% (v/v) for nucleic acid precipitation. The mixture was centrifuged again at 25,155 × g and 4 °C for 10 min, and the resulting supernatant was retained. Solid NaCl was added to the supernatant to a final concentration of 3 M; the mixture was thoroughly vortexed and incubated in a 37 °C water bath, which triggered the formation of abundant white precipitates. The mixture was centrifuged at 25155 × *g* and 37 °C for 10 min, and the precipitate was collected. The precipitate was redissolved in pre-chilled PBS, and the solution was centrifuged at 25,155 × *g* and 4 °C for 10 min to remove insoluble impurities. This ITC cycle was repeated twice more by sequentially adding NaCl to the supernatant to final concentrations of 2 M and 1 M, with all other operations consistent with the above-mentioned procedure. After three rounds of ITC, the protein solution was desalted using a desalting column (Cytiva, USA) to replace PBS with 50 mM Tris-HCl (pH 7.5). The desalted protein was further purified by cation exchange chromatography, followed by endotoxin removal using a Berpharose FF agarose gel column. The purity of all purified proteins was assessed by sodium dodecyl sulfate-polyacrylamide gel electrophoresis (SDS-PAGE), with gels stained using Coomassie Brilliant Blue. Protein concentrations were quantified using the bicinchoninic acid (BCA) assay.

### Mass spectrometry

Protein samples (Lst and Lst-ELP(V)_90_) at a concentration of 1 mg/mL were filtered through a 0.22 μm filter membrane. Subsequently, 5 μL of each sample was pipetted for high-resolution mass spectrometry analysis (Q-TOF/MS, WatersSYNAPTG2-Si, USA). MS mode chose the electrospray ionization mass spectrometry (ESI-MS). ESI source in positive mode; capillary voltage 4500 V; dry gas 8 L/min, 200 °C Molecular weight is represented as the mass-to-charge ratio (m/z). Raw MS data were imported into the Data Analysis software for processing. Each spectrum was calibrated with standards. Three replicate experiments were conducted, and the test results were identical.

### Circular dichroism

Lst, ELP(V)₉₀, and Lst-ELP(V)₉₀ proteins were diluted in ultrapure water to a concentration gradient ranging from 0.01 to 1.0 mg/mL, with the specific concentrations of 0.01, 0.05, 0.1, 0.5, and 1.0 mg/mL applied to each protein sample. To directly evaluate the stability of Lst and Lst-ELP(V)_90_ in PBS, we serially diluted them to concentrations of 0.01, 0.05, 0.1, and 0.5 mg/mL, and then examined the changes in their circular dichroism (CD) spectra on the 0th and 7th days. Circular dichroism spectroscopy was performed on a Jasco International (Japan) spectrometer to characterize the secondary structures of the proteins over a wavelength range of 190–260 nm. All measurements were conducted in triplicate for each concentration.

### Dynamic light scattering

The hydrodynamic diameters of Lst, ELP(V)₉₀, and Lst-ELP(V)₉₀ proteins were determined by dynamic light scattering (DLS) using a Malvern Zetasizer Nano ZSP instrument (Malvern Panalytical, UK) at 15 °C. Protein samples were diluted to a final concentration of 0.1 mg/mL with PBS and filtered through 0.22 μm syringe filters to eliminate particulate impurities. Data acquisition and analysis were performed using Zetasizer Software (Version 7.13). All measurements were conducted in triplicate for each protein sample.

### Thermoresponsive phase transition

Lst-ELP(V)₉₀ samples at a series of concentrations (0.1, 1.0, 5.0, 10, 50, 100, 200, 300, 400 μM) were added to a standard 96-well plate at 200 μL per well. Absorbance at 350 nm (OD₃₅₀) was monitored over a temperature range of 10–60 °C at 2 °C intervals using a microplate reader (SpectraMax M3, USA). To verify the reversibility of the thermoresponsiveness of Lst-ELP(V)₉₀, samples at different concentrations (5.0, 10, 50, 100 μM) were first heated from 10 to 50 °C at 2 °C intervals, and changes in OD₃₅₀ absorbance were then monitored during the subsequent cooling process (back to 10 °C, same temperature interval). The phase transition temperature (*T*ₜ) was defined as the temperature corresponding to 50% of the maximum OD₃₅₀ value attained.

### Broth dilution assay for MIC and MBC determination

A single colony of each pathogen was selected and inoculated into fresh Luria-Bertani (LB) liquid medium. Bacterial cultures of MRSA (ATCC, 33591), MRSE (ATCC, 35984) and VRSA(LC1203) were diluted with LB medium to an optical density at 600 nm (OD₆₀₀) of ~0.8. Aliquots of the bacterial suspension were dispensed into a 96-well microplate at a final volume of 100 μL per well, followed by the addition of Lst or Lst-ELP(V)₉₀ at a series of gradient concentrations (0.5, 1.0, 1.5, 2.0, 2.5, and 3.0 nM). The microplate was incubated at 37 °C for a total duration of 6 h. Bacterial growth inhibition was assessed by measuring OD₆₀₀ at 1-h intervals using a microplate reader (SpectraMax M3, USA). Minimum inhibitory concentration (MIC) and minimum bactericidal concentration (MBC) values were calculated from the resultant growth inhibition curves. Each concentration was tested in triplicate wells to ensure experimental reproducibility. Morphological alterations of planktonic bacteria following treatment with Lst or Lst-ELP(V)₉₀ were visualized using scanning electron microscopy (SEM; JSM-IT200, JEOL, Japan).

### Resistance induction assay

After the MRSA strains formed single colonies on agar plates, individual colonies were picked and inoculated into liquid LB medium for overnight culture to prepare seed cultures. Lst (0.25 nM), Lst-ELP(V)_90_ (0.25 nM), and Van (0.175 μM) at 0.5× MIC concentrations were separately added to 15 mL tubes containing 2 mL of fresh LB medium. Each drug concentration was set up in triplicate. Bacteria were then inoculated at a volume ratio of 1:100 and cultured for 24 h before being transfer into new tubes. This subculturing process was continued for 30 days (approximately 150 generations). The MIC values of the bacterial cultures against the respective drugs were measured on 0, 5, 10, 15, 20, 25, and 30 days. A curve showing the change in MIC values over the passaging time was plotted.

### Bactericidal effects for stationary phase and persister bacteria

MRSA was cultured to mid-logarithmic phase. The cells were harvested by centrifugation, washed three times with PBS, and resuspended in nutrient-poor LB medium (containing one-tenth the nutrients of standard LB, sufficient only for basic bacterial maintenance). The culture was then starved for 48 h, during which OD_600_ value was measured at 12-h intervals to generate a growth curve. Once the OD_600_ value stabilized, indicating entry into stationary phase, the 48-h starved culture was inoculated at 1% (v/v) into fresh, nutrient-rich LB medium to assess growth recovery. For the killing assay, after 48 h of starvation, Lst-ELP(V)_90_ was added at the minimum bactericidal concentration (MBC). OD_600_ value was recorded hourly, and samples collected at each time point were plated on agar for colony-forming unit (CFU) enumeration. The reduction in CFU relative to the control was used to quantify the bactericidal activity of Lst-ELP(V)_90_ against stationary-phase bacteria.

### Agar diffusion assay for stability determination

MRSA was cultured overnight at 37 °C in LB liquid medium. LB agar plates were prepared by mixing 20 mL of melted agar with the bacterial culture at a ratio of 1% (v/v), followed by immediate pouring into sterile Petri dishes. For the assessment of protein stability, Lst and Lst-ELP(V)₉₀ proteins (5 μM) dissolved in PBS were incubated at 37 °C; samples were collected at 4-day intervals over a 32-day period. Similarly, the stability of Lst and Lst-ELP(V)₉₀ (5 μM) in serum was evaluated via incubation at 37 °C for 3 h, with aliquots collected at 0.5-h intervals. All collected samples were subjected to the agar diffusion assay: 15 μL aliquots of each sample were spotted onto the prepared MRSA-containing agar plates, and the changes in antimicrobial activity of the proteins were determined based on the diameter of the growth inhibition zones.

Additionally, Lst and Lst-ELP(V)₉₀ proteins were prepared at a concentration of 50 μM in serum and incubated at 37 °C, a condition that triggered the phase transition of Lst-ELP(V)₉₀. The phase-separated Lst-ELP(V)₉₀ solution was centrifuged at 25,155 × *g* and 4 °C for 10 min to pellet the coacervate at the bottom of the tube, thereby simulating a drug depot system. Samples were collected from this system at 6-day intervals over a 30-day period, and residual antibacterial activity was assessed by measuring the inhibition zone diameter using the agar diffusion assay described above. Additionally, the stability of Lst and Lst-ELP(V)₉₀ (5 μM) in liver homogenate was evaluated via incubation at 37 °C within 30 days, with aliquots collected at 6-day intervals. Serum and the liver homogenate served as the negative control in all antibacterial activity assays. Residual bioactivity of the proteins was calculated using the following formula:

$${{{\rm{Residual}}}}\,\,{{{\rm{bioactivity}}}}\left(\%\right)=\frac{{{{{\rm{d}}}}}_{{{{\rm{sample}}}}}}{{{{{\rm{d}}}}}_{{{{\rm{control}}}}}}\times 100\%$$

where

d_control_: Initial inhibition zone diameter of the protein prior to incubation;

d_sample_: Inhibition zone diameter of the protein following incubation.

### Biofilm elimination experiments in vitro

Methicillin-resistant *Staphylococcus aureus* (MRSA, ATCC 33591) biofilms were used as an in vitro model to evaluate the antibiofilm activity of vancomycin (Van), ELP(V)₉₀, Lst, and Lst-ELP(V)₉₀. Briefly, MRSA was streaked onto LB agar plates to obtain single colonies; a single colony was then picked and cultured overnight in LB liquid medium at 37 °C. The MRSA bacterial suspension was adjusted to a concentration of 1 × 10^8^ CFU/mL, and 200 μL aliquots were inoculated into 96-well plates for static incubation at 37 °C for 72 h to form mature biofilms. Planktonic bacteria were removed via three sequential washes with sterile phosphate-buffered saline (PBS). Subsequently, ELP(V)₉₀, Lst, and Lst-ELP(V)₉₀ were added to the wells at final concentrations of 0.1, 0.5, 1.0, 2.0, and 5.0 μM, respectively, while Van was supplemented at final concentrations of 0.1, 1.0, 10.0, 20.0, and 50.0 μM. The plates were incubated at 37 °C for a total of 6 h, with samples collected at 1-h intervals for subsequent analysis. For biofilm quantification, 200 μL of 0.1% crystal violet solution was added to each well, followed by incubation for 20 min at room temperature. Excess unbound stain was removed by gently washing the plate three times with PBS; thereafter, 200 μL of 95% ethanol was added to each well to solubilize the adherent crystal violet, with incubation for 30 min at room temperature. Finally, the absorbance at 595 nm (OD₅₉₅) was measured using a microplate reader. Pathogens of MRSE (ATCC, 35984) and VRSA(LC1203) biofilm elimination experiments operation was consistent with the MRSA.

The residual biofilm rate was calculated using the following formula:

$${{{\rm{Residual}}}}\,\,{{{\rm{biofilmrate}}}}\left(\%\right)=\frac{{{{{\rm{OD}}}}}_{{{{\rm{sample}}}}}}{{{{{\rm{OD}}}}}_{{{{\rm{control}}}}}}\times 100\%$$

where

OD_control_: OD_595_ of the un-treated MRSA biofilm group.

OD_sample_: OD_595_ of the MRSA biofilm group treated with the test agent.

### MBIC and MBEC experiments

Minimum biofilm inhibition concentrations (MBIC) and Minimum biofilm eradication concentrations (MBEC) values were carried out as follows. For the determination of MBIC values, gradient dilutions (0.01, 0.1, 1.0, 2.0, 4.0, 8.0, 16.0 μM) of Lst and Lst-ELP(V)_90_, as well as vancomycin (0.1, 1.0, 5.0, 10.0, 20.0, 50.0, 100.0 μM), were added during biofilm cultivation. After crystal violet staining, the OD_595_ values were measured. Using the PBS group as the control, the biofilm removal rate at each concentration was calculated. For the determination of MBEC values, after the biofilm had fully matured, the corresponding gradient-diluted drugs mentioned above were added. The released viable bacteria from the biofilm were subjected to colony counting. Compared with the PBS group, the concentration corresponding to a three-log reduction in colony count is the MBEC value. MBIC is defined as the minimum concentration of gradient-diluted drugs added during biofilm cultivation that results in a biofilm removal rate of ≥50%. The minimum biofilm eradication concentration is defined as the lowest concentration of gradient-diluted drugs added to a mature biofilm that leads to a three-log reduction in viable bacteria, as determined by colony counting, compared with the PBS group.

### SEM and CLSM analysis of biofilm elimination

A single colony of MRSA was picked and inoculated into LB liquid medium for overnight incubation at 37 °C. Sterile titanium alloy discs were placed at the bottom of 24-well plates prior to inoculation, after which the bacterial suspension was transferred into the plates at a 1% (v/v) ratio. The plates were statically incubated at 37 °C for 3 days to allow biofilm formation on the disc surfaces.

Subsequently, Lst analogs were added to the wells at a final concentration of 5 μM, while Van was supplemented at a final concentration of 50 μM. The plates were incubated for an additional 2 h, followed by thorough washing with PBS at least three times to remove planktonic bacteria and cellular debris. For sample fixation, the titanium alloy discs with adherent biofilms were incubated in 4% glutaraldehyde solution at 4 °C overnight. After fixation, the discs were rinsed three times with PBS, and gradient ethanol dehydration was performed sequentially using 30, 50, 70, 80, 90, and 100% (v/v) ethanol aqueous solutions, with each dehydration step lasting 15 min. The dehydrated discs were mounted onto SEM sample stubs, followed by gold sputtering coating using an ion sputter coater (Cressington, UK). Finally, the biofilm morphology on the disc surfaces was observed and imaged using a scanning electron microscope (SEM; JSM-IT200, JEOL, Japan).

For three-dimensional (3D) structural visualization of MRSA biofilms, mature biofilms were first established on the bottom of specialized confocal dishes. The biofilms were then treated with ELP(V)₉₀ (5 μM), Van (20 μM), Lst (5 μM), and Lst-ELP(V)₉₀ (5 μM) at 37 °C for 3 h. After treatment, the dishes were washed three times with PBS to remove unbound agents, and the biofilms were stained with a SYTO 9/propidium iodide (PI) staining kit in the dark for 30 min. Residual dye was removed by three additional PBS washes, and the dishes were wrapped in aluminum foil to avoid light exposure. The 3D architecture of the stained biofilms was visualized using a confocal laser scanning microscope (CLSM; Olympus FV3000, Japan).

### Maximum tolerated dose determination

Graded doses of Lst (0, 2, 4, 8, 16, 32, 64, 128 μmol/kg) and Lst-ELP(V)₉₀ (0, 2, 4, 8, 16, 32, 64, 128, 256 μmol/kg) were administered subcutaneously to mice, with 3 mice per group (*n* = 3). Body weights were measured every 3 days over a 30-day period. Each mouse’s body weight was measured in triplicate, and the mean value of the three measurements was calculated for data analysis. Mice were humanely euthanized if their body weight loss reached or exceeded 10% of their initial body weight, in accordance with animal welfare guidelines.

### Biodistribution and pharmacokinetics

Male Kunming mice were subcutaneously administered the maximum tolerated dose (MTD) of Cy5-labeled Lst (32 μmol/kg) and Cy5-labeled Lst-ELP(V)₉₀ (128 μmol/kg), respectively. At predetermined time points (1, 3, 7, 14, 21, and 28 days) post-administration, mice were anesthetized with inhalational isoflurane, and fluorescent signals were acquired using an in vivo imaging system (IVIS Spectrum, PerkinElmer, USA).

For pharmacokinetic analysis, blood samples were collected from the tail vein at specific time points (5, 10, 15, 20, 25, 30, 100, 148, 196, 244, 292, 384, 528, and 672 h post-administration). Blood samples were allowed to clot at room temperature for 1 h to isolate serum, followed by centrifugation at 1006.2 × g and 4 °C for 20 min. A 15 μL aliquot of the serum supernatant was transferred to a black opaque 384-well plate, and fluorescence intensity was measured using a microplate reader with excitation at 646 nm and emission at 650 nm. The concentrations of Cy5-labeled Lst and Lst-ELP(V)₉₀ in serum samples were calculated using a Cy5 standard curve (*y* = 297.54x + 271.84, *R*² = 0.9832), where *x* represents the Cy5-labeled protein concentration and *y* represents the measured fluorescence intensity.

To investigate the in vivo distribution of Cy5-labeled Lst and Lst-ELP(V)₉₀, mice were humanely sacrificed at 1 day and 7 days post-administration, respectively, and major organs (heart, lung, spleen, kidney, and liver) were harvested. The collected organs were subjected to fluorescence imaging using the same IVIS Spectrum in vivo imaging system (PerkinElmer, USA) for quantitative and qualitative analysis of protein distribution.

### Immunogenicity analysis

Six-week-old male Kunming mice were randomly divided into three groups, which received three subcutaneous injections of Lst, Lst-ELP(V)₉₀ (each at a dose of 10 μmol/kg body weight), or vehicle control, respectively, with a two-week interval between consecutive injections. At the end of the immunization period, blood samples were collected for immunogenicity evaluation using an enzyme-linked immunosorbent assay (ELISA).

Antigen working solution was prepared at 1 μg/mL in coating buffer, and 0.1 mL of this solution was added to each well of a polystyrene microplate for overnight incubation at 4 °C to coat the wells. Serum was isolated from blood samples by allowing clot formation at room temperature for 30 min, followed by centrifugation at 1006.2 × *g* and room temperature for 10 min. After overnight coating, the antigen solution was discarded by aspiration, and each well was washed three times with wash buffer. The wells were then blocked with blocking buffer at 37 °C for 2 h; after blocking, the plate was washed again as described above. Serially diluted serum samples were added to the wells and incubated at 37 °C for 2 h. Following another round of washing, 0.1 mL of freshly diluted horseradish peroxidase (HRP)-conjugated anti-mouse IgG/IgM secondary antibody was added to each well, and the plate was incubated at 37 °C for 1 h.

After the final wash step, 0.1 mL of 3,3′,5,5′-tetramethylbenzidine (TMB) substrate solution was added to each well to initiate the colorimetric reaction, which proceeded at 37 °C for 15 min. The reaction was terminated by adding 100 μL of 0.2 M sulfuric acid to each well. The optical density (OD) values of each well were measured at 450 nm (test wavelength) using a microplate reader (SpectraMax M3, USA). The OD₆₃₀ value was used as a reference to subtract background absorbance from the OD₄₅₀ value for data normalization.

### Cellular uptake by macrophages

The cellular uptake efficiency of Cy5-labeled Lst and Lst-ELP(V)₉₀ was evaluated using RAW264.7 murine macrophage cells. For confocal laser scanning microscopy (CLSM) imaging, cells were seeded into 35-mm glass-bottom dishes at a density of 5 × 10⁴ cells/mL and allowed to adhere overnight in a 37 °C, 5% CO₂ humidified incubator. On the following day, cells were rinsed twice with PBS to remove non-adherent cells, then incubated with 1 μM Cy5-labeled Lst or Lst-ELP(V)₉₀ for 3 h under the same culture conditions (37 °C, 5% CO₂). After incubation, unbound fluorescent proteins were thoroughly washed away with PBS, and cell morphology was visualized under bright-field illumination. Cy5 fluorescence intensity was quantified with excitation at 633 nm and emission at 650 nm using CLSM.

For flow cytometry analysis, RAW264.7 cells were seeded into 12-well plates at the same density (5 × 10⁴ cells/mL) and allowed to adhere for approximately 8 h in the 37 °C, 5% CO₂ incubator. Cells were treated separately with 1 μM Cy5-labeled Lst or Lst-ELP(V)₉₀ for 3 h under standard culture conditions. Subsequently, cells were gently rinsed twice with PBS, detached using a cell scraper, and centrifuged at 600 × *g* (4 °C, 10 min) to collect cell pellets. The pellets were fixed with 2% paraformaldehyde (PFA) in PBS at room temperature for 15 min to preserve cell integrity, rinsed twice more with PBS, and finally gently resuspended in flow cytometry staining buffer. The Cy5 fluorescence intensity in the cells was quantified using a Beckman Coulter CytoFlex S flow cytometer (Brea, CA, USA).

### Activation of macrophages

RAW264.7 murine macrophage cells were seeded at a density of 1 × 10⁶ cells per well in 35-mm glass-bottom dishes and cultured overnight in a 37 °C, 5% CO₂ humidified incubator. Following a 6-h treatment with 1 μM Lst, ELP(V)₉₀, or Lst-ELP(V)₉₀, cells were rinsed twice with PBS to remove unbound proteins, then fixed with 4% PFA in PBS for 15 min at room temperature (RT). After two additional PBS rinses, non-specific binding sites were blocked with 3% bovine serum albumin (BSA) in PBS for 30 min at RT.

Cells were subsequently incubated overnight at 4 °C with primary antibodies targeting CD86 (1:100 dilution in blocking buffer, clone name:ARC54011) or CD206 (1:200 dilution in blocking buffer, clone name:ATC0035). Unbound primary antibodies were removed by three consecutive PBS washes, after which cells were stained with iFluor® 594-conjugated wheat germ agglutinin (WGA; 5 μg/mL in PBS) for 15 min at RT to label the plasma membrane. To preserve the staining pattern, cells were re-fixed with 4% PFA in PBS for 15 min at RT, followed by counterstaining of nuclei with 300 nM 4’,6-diamidino-2-phenylindole (DAPI) for 15 min at RT in the dark. After a final round of PBS rinses, high-resolution fluorescent images were acquired using a Zeiss LSM880 confocal laser scanning microscope (CLSM) equipped with an Airyscan detector (Zeiss, Oberkochen, Germany).

### Inflammatory response for Lst and Lst-ELP(V)_90_

Bacterial LPS was used to mimic inflammation induced by bacterial infection, with the target being Raw264.7 macrophages. The experiment was divided into five groups: the first group consisted of macrophages only plus culture medium, reflecting the baseline level of inflammatory factor production by macrophages; the second group was was macrophages plus LPS; the third group was macrophages plus LPS, with the addition of ELP(V)_90_; the fourth group was macrophages plus LPS, with the addition of Lst; and the fifth group was macrophages plus LPS, with the addition of Lst-ELP(V)_90_. The volume of each well was 200 μL. Proteins of the Lst, ELP(V)_90_, and Lst-ELP(V)_90_ were added at equimolar concentrations (1 μM). Three replicates were prepared for each of the above groups. Subsequently, the cells were cultured in a CO₂ incubator for 6 h, and the expression levels of the inflammatory factors TNF-α, IL-6, and IFN-γ were measured using ELISA kits. Graphs were plotted to compare the differences among the groups.

### Cytotoxicity evaluation

Mouse embryonic osteoblast cell line MC3T3-E1 was employed to assess the in vitro cytotoxicity of Lst, ELP(V)₉₀, and Lst-ELP(V)₉₀. Cells were seeded at a density of 5 × 10³ cells per well in 96-well plates and cultured in complete Dulbecco’s modified Eagle’s medium (DMEM), supplemented with 10% fetal bovine serum (FBS) and 1% penicillin-streptomycin solution. The plates were incubated in a humidified incubator at 37 °C with 5% CO₂ for 24 h to allow cell adhesion and proliferation.

Subsequently, the culture medium was replaced with fresh complete DMEM containing serial concentrations of Lst, ELP(V)₉₀, or Lst-ELP(V)₉₀, and the cells were incubated for an additional 24 h under the same conditions. After treatment, 10 μL of CCK-8 reagent was added to each well, followed by incubation at 37 °C for 2 h to allow color development. The absorbance of each well was measured at 450 nm using a SpectraMax M3 microplate reader (Molecular Devices, USA).

Cell viability was calculated using the following formula:

$$\frac{\left({{{{\rm{OD}}}}}_{{{\rm{sample}}}}-{{{{\rm{OD}}}}}_{{{\rm{blank}}}}\right)}{\left({{{{\rm{OD}}}}}_{{{\rm{control}}}}-{{{{\rm{OD}}}}}_{{{\rm{blank}}}}\right)}\times 100\%$$

where

OD_sample_: Absorbance of wells containing cells, complete medium, target protein, and CCK-8 reagent;

OD_blank_: Absorbance of wells containing only complete medium and CCK-8 reagent (no cells);

OD_control_: Absorbance of wells containing cells, complete medium, and CCK-8 reagent (no target protein).

### Hemolytic activity assessment

Rabbit red blood cells were washed with PBS via centrifugation (252 × *g*, 5 min, 4 °C) at least five times, until the supernatant appeared completely clear, to remove residual plasma components. The washed red blood cells were then diluted with PBS to a 10% (v/v) suspension and immediately used for the hemolytic activity assay.

Lst, ELP(V)₉₀, and Lst-ELP(V)₉₀ proteins were individually dissolved in PBS to yield a concentration gradient ranging from 1 to 400 μM. Equal volumes of each protein solution were mixed with the 10% red blood cell suspension, and the mixtures were transferred to a 96-well plate. The plate was incubated in a humidified 37 °C, 5% CO₂ incubator for a total of 12 h. For experimental controls, 0.5% Triton ×-100 and PBS alone were included as the positive control (complete hemolysis) and negative control (no hemolysis), respectively.

Following incubation, the absorbance at 415 nm (a wavelength optimized for the detection of rabbit hemoglobin) was measured using a SpectraMax M3 microplate reader (Molecular Devices, USA), with all measurements performed in triplicate to ensure data reproducibility. The hemolytic activity was calculated using the following formula:

$$\frac{\left({{{{\rm{OD}}}}}_{{{\rm{test}}}}-{{{{\rm{OD}}}}}_{{{\rm{negative}}}}\right)}{\left({{{{\rm{OD}}}}}_{{{\rm{positive}}}}-{{{{\rm{OD}}}}}_{{{\rm{negative}}}}\right)}\times 100\%$$

where

OD_test_: Absorbance of wells containing red blood cells, PBS, and the target protein;

OD_negative_: Absorbance of wells containing red blood cells and PBS only (no protein);

OD_positive_: Absorbance of wells containing red blood cells and 0.5% Triton ×-100 (complete lysis control).

### Biosafety evaluation

Mice were subcutaneously administered the maximum tolerated doses (MTDs) of Lst and Lst-ELP(V)₉₀, respectively. For ELP(V)₉₀, the injection dose was set to match the molar concentration of Lst-ELP(V)₉₀; Van was administered at a dose of 50 mg/kg based on previous studies. On day 28 post-administration, blood samples (with six mice per group, *n* = 6) and major tissue samples (heart, liver, spleen, lung, and kidney) were collected for hematological and histopathological analyses.

Hematological parameters, including red blood cells (RBCs), white blood cells (WBCs), hemoglobin (HGB), platelets (PLTs), neutrophils (Neu), basophils (Bas), monocytes (Mon), lymphocytes (Lym), and eosinophils (Eos), were quantified using an automatic hematology analyzer (Beckman-Coulter, USA). For liver and kidney function assessments, serum levels of alanine aminotransferase (ALT), aspartate aminotransferase (AST), creatinine (Crea), and blood urea nitrogen (Urea) were measured using standard clinical biochemical assays.

Fresh tissue blocks were immersion-fixed in 4% PFA in PBS at room temperature overnight for histopathological processing. Following fixation, the tissues were embedded in paraffin, sectioned into 4-μm slices, and stained with H&E. The stained paraffin sections were imaged and scanned using a VECTRA automated Quantitative Pathology Imaging System (PerkinElmer, USA) for morphological observation and quantitative analysis.

### MRSA infection treatment in vivo

Three days prior to surgery, titanium alloy discs were placed in 24-well plates and co-cultured with MRSA to form mature biofilms, following the same protocol described above. Meanwhile, experimental mice were housed under standard pathogen-free conditions for acclimatization. Mice were first anesthetized via intraperitoneal injection of 5% tribromoethanol (17.8 mL/kg body weight). After confirming deep anesthesia, the dorsal fur was shaved and the skin was disinfected sequentially with iodine tincture and 75% ethanol. Under aseptic conditions in a laminar flow hood, a 0.5–1 cm skin incision was made using a sterile surgical scalpel. A titanium alloy disc with pre-formed biofilm was subcutaneously implanted into the incision, and the wound was sutured with sterile surgical sutures. On day 1, 200 μL of the respective test agent PBS, ELP(V)₉₀, Van, Lst, and Lst-ELP(V)₉₀ was subcutaneously injected adjacent to the implant site. Finally, mice were returned to their cages for postoperative recovery, with the surgical day designated as day 0.

Body weight and wound size of mice in all groups were measured and recorded at predetermined time points (1, 3, 7, 14, 21, and 28 d post-surgery). Changes in body weight and wound area were monitored throughout the experimental period to assess general health and wound healing. On day 28, mice were humanely euthanized, and the titanium alloy discs were aseptically removed in a biological safety cabinet.

Each explanted disc was placed into a sterile centrifuge tube containing sterile PBS, followed by vortex for 5 min to dislodge adherent bacteria. After centrifugation at 1006.2 × *g* and 4 °C for 10 min, the bacterial pellet was collected, serially diluted, and plated onto LB agar plates for viable bacterial colony counting (CFU assay). Separately, the explanted discs were subjected to crystal violet staining for biofilm quantification: after staining and elution, the absorbance was measured at 595 nm (OD₅₉₅) using a microplate reader. Additionally, the explanted discs were observed via SEM as described previously to evaluate biofilm morphology.

To assess systemic infection, serum levels of pro-inflammatory cytokines (TNF-α, IL-6, and IFN-γ) were measured using enzyme-linked immunosorbent assay (ELISA) kits, with serum from healthy, non-surgically treated mice as the control. Hematological analysis of blood samples was also performed using an automatic hematology analyzer (Beckman-Coulter, USA) as described earlier.

### Bacteremia assay

On the final day of treatment, the mouse tail was first disinfected with povidone-iodine, and approximately 100 μL of blood was collected under a biological safety cabinet. The blood sample was centrifuged at speed of 447.2 × g for 5 min to allow erythrocyte sedimentation. The supernatant was then plated onto solid agar plates and incubated overnight at 37 °C. The following day, bacterial colonies were counted to assess the severity of sepsis in the mice based on the number of colonies in the blood. The number of viable bacteria in the blood was compared among different groups to evaluate the therapeutic efficacy.

### Hematoxylin-eosin and Masson staining and immunohistochemistry

Tissue samples, including wound skin and major internal organs, were fixed in 4% PFA in PBS for 48 h at room temperature, followed by gradient dehydration in an ascending ethanol series (30, 50, 70, 80, 90, 95, and 100% v/v), clearing in xylene, and embedding in paraffin wax. The paraffin-embedded tissue blocks were sectioned into 4-μm-thick slices using a rotary microtome, and the sections were mounted onto glass slides for subsequent staining.

For H&E staining, the sections were subjected to routine deparaffinization and rehydration procedures: sequential immersion in xylene (twice, 10 min each) for deparaffinization, followed by rehydration through a descending ethanol series (100, 95, 80, 70% v/v, 5 min each) and a final rinse in distilled water. The sections were stained with hematoxylin working solution for 3–5 min to visualize cell nuclei, followed by rinsing under running tap water for 5 min. Subsequently, the sections were differentiated in 1% acid alcohol for 10–15 s to enhance nuclear staining contrast, and then blued in weak ammonia solution until the nuclei turned bright blue. The sections were counterstained with eosin working solution for 1–3 min to stain the cytoplasm, followed by dehydration through an ascending ethanol series (70, 80, 90, 95, 100% v/v, 5 min each), clearing in xylene (twice, 10 min each), and mounting with neutral balsam. Histopathological evaluation of the stained sections was performed under a light microscope (Olympus, Japan).

For Masson’s trichrome staining (to assess collagen deposition), sections were subjected to the same deparaffinization and rehydration steps described above. The rehydrated sections were stained with hematoxylin working solution for 5–10 min to label nuclei, followed by a rinse in distilled water. The sections were then stained with scarlet-acid fuchsin solution for 5–10 min to stain cytoplasm and muscle fibers red. Next, the sections were differentiated in phosphomolybdic-phosphotungstic acid solution for 10–15 min; this step selectively removes the red stain from collagen fibers while retaining it in other tissue components. Without intermediate washing, the sections were immediately transferred to aniline blue solution and stained for 5–10 min to color collagen fibers blue. The sections were rinsed briefly in distilled water, then differentiated in 1% acetic acid for 1–2 min to intensify the color contrast, followed by dehydration, clearing, and mounting using the same protocol as for H&E staining. Collagen deposition was identified by blue-stained regions under light microscopy, and the staining intensity was quantified for semi-quantitative analysis of tissue repair.

For immunohistochemistry assay, deparaffinized and rehydrated mouse skin sections were subjected to heat-induced antigen retrieval in citrate buffer (pH 6.0). After blocking endogenous peroxidase and non-specific sites, sections were incubated overnight at 4 °C with primary antibodies against mouse TNF-α or IL-6. Following PBS washes, HRP-conjugated secondary antibody was applied. Antigen localization was visualized using DAB (brown precipitate), and nuclei were counterstained with hematoxylin (blue). Specificity was confirmed by positive control tissue and a no-primary-antibody negative control.

### Transcriptomic analysis

Transcriptome analysis was performed on wounded skin tissues from each group upon treatment completion. The wounded skin tissues were dissected with sterile surgical scissors and immediately transferred into RNase-free centrifuge tubes. To preserve RNA integrity, the tubes were rapidly immersed in liquid nitrogen, and the entire tissue collection process was completed within 5 min. The samples were then stored at −80 °C until further use. Total RNA extraction, RNA sequencing, and bioinformatic data processing were outsourced to Liebing Biotech Co., Ltd. (Shangrao, China).

To investigate treatment-induced transcriptional alterations, transcriptome profiles of the Lst and Lst-ELP(V)₉₀ groups were compared with that of the healthy group, with a focus on identifying the most significantly upregulated genes. Additionally, key inflammatory signaling pathways (NF-κB and IL-17 pathways) were analyzed, emphasizing the upregulation of inflammatory pathways in the Lst group and their downregulation in the Lst-ELP(V)₉₀ group. Subsequently, the top 13 upregulated genes in these two signaling pathways were selected for targeted analysis.

Global transcriptional analysis was further performed on all genomic genes, focusing on three aspects: comparing the top four genes with the highest transcriptional levels between the Lst and Lst-ELP(V)₉₀ groups, and analyzing the top 13 highly transcribed genes associated with wound healing and skin regeneration.

### Reporting summary

Further information on research design is available in the Nature Portfolio Reporting Summary linked to this article.

## Supplementary information

Supplementary Information

Reporting Summary

Transparent Peer Review file

## Source data

Source Data

## Data Availability

All data supporting the findings of this study are available within the Article and its supplementary files. The nucleotide sequences of the synthetic constructs generated in this study have been deposited in the NCBI GenBank database under accession numbers PZ629001 (LST gene construct) and PZ629002 (LST_ELP_V90 construct). The RNA sequencing data generated in this study have been deposited in the NCBI Sequence Read Archive (SRA) database under BioProject accession number PRJNA1492776. The dataset includes transcriptomic profiles from PBS, lysostaphin (Lst), and LstELP treatment groups. The protein mass spectrometry molecular weight data generated in this study have been deposited in the PRIDE repository under accession number PXD080789. Any additional requests for information can be directed to and will be fulfilled by the corresponding authors. Source data are provided with this paper.

## References

[1] Chambers, H. F. & Deleo, F. R. Waves of resistance: *Staphylococcus aureus* in the antibiotic era. *Nat. Rev. Microbiol.* **9**, 629–641 (2009). 10.1038/nrmicro2200 PMC2871281 19680247

[2] Lee, A. S., Otto, M., Ong, D. S. Y., Takeuchi, K. A. & See, W. D. C. Methicillin-resistant *Staphylococcus aureus*. *Nat. Rev. Dis. Prim.* **4**, 18033 (2018). 29849094 10.1038/nrdp.2018.33

[3] Turner, N. A. et al. Methicillin-resistant *Staphylococcus aureus*: an overview of basic and clinical research. *Nat. Rev. Microbiol.* **4**, 203–218 (2019). 10.1038/s41579-018-0147-4 PMC6939889 30737488

[4] Parsons, J. B., Mourad, A., Conlon, B. P., Kielian, T. & Fowler, V. G. Jr Methicillin-resistant and susceptible *Staphylococcus aureus*: tolerance, immune evasion and treatment. *Nat. Rev. Microbiol.***24**, 397–409 (2026).10.1038/s41579-025-01226-240835978

[5] Arciola, C. R., Campoccia, D. & Montanaro, L. Implant infections: adhesion, biofilm formation and immune evasion. *Nat. Rev. Microbiol.* **16**, 397–409 (2018). 29720707 10.1038/s41579-018-0019-y

[6] Magill, S. S. et al. Multistate point-prevalence survey of health care-associated infections. *N. Engl. J. Med.* **13**, 1198–1208 (2014). 10.1056/NEJMoa1306801 PMC4648343 24670166

[7] Flemming, H. C. & Wingender, J. The biofilm matrix. *Nat. Rev. Microbiol.* **9**, 623–633 (2010). 10.1038/nrmicro2415 20676145

[8] Koo, H., Allan, R. N., Howlin, R. P., Stoodley, P. & Hall-Stoodley, L. Targeting microbial biofilms: current and prospective therapeutic strategies. *Nat. Rev. Microbiol.* **12**, 740–755 (2017). 10.1038/nrmicro.2017.99 PMC5685531 28944770

[9] Darouiche, R. O. Treatment of infections associated with surgical implants. *N. Engl. J. Med.* **350**, 1422–1429 (2004). 10.1056/NEJMra035415 15070792

[10] Stewart, P. S. & Costerton, J. W. Antibiotic resistance of bacteria in biofilms. *Lancet* **9276**, 135–138 (2001). 10.1016/s0140-6736(01)05321-1 11463434

[11] Guarner, F. & Malagelada, J. R. Gut flora in health and disease. *Lancet* **9356**, 512–519 (2003). 10.1016/S0140-6736(03)12489-0 12583961

[12] Rumbaugh, K. P. & Sauer, K. Biofilm dispersion. *Nat. Rev. Microbiol.* **10**, 571–586 (2020). 10.1038/s41579-020-0385-0 PMC8564779 32533131

[13] Schindler, C. A. & Schuhardt, V. T. Lysostaphin: a new bacteriolytic agent for the *Staphylococcus*. *Proc. Natl. Acad. Sci. USA* **51**, 414–421 (1964). 10.1073/pnas.51.3.414 PMC300087 14171453

[14] Kumar, J. K. Lysostaphin: an antistaphylococcal agent. *Appl. Microbiol. Biotechnol.* **80**, 555–561 (2008). 18607587 10.1007/s00253-008-1579-y

[15] Kusuma, C., Jadanova, A., Chanturiya, T. & Kokai-Kun, J. F. Lysostaphin-resistant variants of *Staphylococcus aureus* demonstrate reduced fitness in vitro and in vivo. *Antimicrob. Agents Chemother.* **51**, 475–482 (2007). 17101683 10.1128/AAC.00786-06 PMC1797764

[16] Climo, M. W., Ehlert, K. & Archer, G. L. Mechanism and suppression of lysostaphin resistance in oxacillin-resistant *Staphylococcus aureus*. *Antimicrob. Agents Chemother.* **45**, 1431–1437 (2001). 11302806 10.1128/AAC.45.5.1431-1437.2001 PMC90484

[17] Stranden, A. M., Ehlert, K., Labischinski, H. & Berger-Bächi, B. Cell wall monoglycine cross-bridges and methicillin hypersusceptibility in a femAB null mutant of methicillin-resistant *Staphylococcus aureus*. *J. Bacteriol.* **179**, 9–16 (1997). 8981974 10.1128/jb.179.1.9-16.1997 PMC178655

[18] Ling, B. & Berger-Bächi, B. Increased overall antibiotic susceptibility in *Staphylococcus aureus femAB*null mutants. *Antimicrob. Agents Chemother.* **42**, 936–938 (1998). 9559813 10.1128/aac.42.4.936 PMC105572

[19] Bastos, M. D., Coutinho, B. G. & Coelho, M. L. Lysostaphin: a staphylococcal bacteriolysin with potential clinical applications. *Pharmaceuticals* **3**, 1139–1161 (2010). 27713293 10.3390/ph3041139 PMC4034026

[20] Stark, F. R., Thornsvard, C., Flannery, E. P. & Artenstein, M. S. Systemic lysostaphin in man-apparent antimicrobial activity in a neutropenic patient. *N. Engl. J. Med.* **14**, 239–240 (1974). 10.1056/NEJM197408012910507 4525537

[21] Harrison, E. F., Fuquay, M. E. & Zygmunt, W. A. Antigenic response to topically applied proteins. *Infect. Immun.* **11**, 309–312 (1975). 163218 10.1128/iai.11.2.309-312.1975 PMC415062

[22] Daley, M. J. & Oldham, E. R. Lysostaphin: immunogenicity of locally administered recombinant protein used in mastitis therapy. *Vet. Immunol. Immunopathol.* **31**, 301–312 (1992). 1589957 10.1016/0165-2427(92)90017-k

[23] Zha, J., Li, J., Su, Z., Akimbekov, N. & Wu, X. Lysostaphin: engineering and potentiation toward better applications. *J. Agric. Food Chem.* **70**, 11441–11457 (2022). 36082619 10.1021/acs.jafc.2c03459

[24] Zhao, H. et al. Depletion of T cell epitopes in lysostaphin mitigates anti-drug antibody response and enhances antibacterial efficacy in vivo. *Chem. Biol.* **22**, 629–639 (2015). 26000749 10.1016/j.chembiol.2015.04.017 PMC4441767

[25] Blazanovic, K. et al. Structure-based redesign of lysostaphin yields potent antistaphylococcal enzymes that evade immune cell surveillance. *Mol. Ther. Methods Clin. Dev.* **2**, 15021 (2015). 26151066 10.1038/mtm.2015.21 PMC4470366

[26] Choi, Y., Verma, D., Griswold, K. E. & Bailey-Kellogg, C. EpiSweep: computationally driven reengineering of therapeutic proteins to reduce immunogenicity while maintaining function. *Methods Mol. Biol.* **1529**, 375–398 (2017). 27914063 10.1007/978-1-4939-6637-0_20 PMC5142831

[27] Zhao, H. et al. Globally deimmunized lysostaphin evades human immune surveillance and enables highly efficacious repeat dosing. *Sci. Adv.* **6**, eabb9011 (2020). 32917596 10.1126/sciadv.abb9011 PMC7467700

[28] Satishkumar, R. et al. Evaluation of the antimicrobial activity of lysostaphin-coated hernia repair meshes. *Antimicrob. Agents Chemother.* **55**, 4379–4385 (2011). 21709102 10.1128/AAC.01056-10 PMC3165354

[29] Belyansky, I. et al. The addition of lysostaphin dramatically improves survival, protects porcine biomesh from infection, and improves graft tensile shear strength. *J. Surg. Res.* **171**, 409–415 (2011). 21696759 10.1016/j.jss.2011.04.014

[30] Windolf, C. D., Lögters, T., Scholz, M., Windolf, J. & Flohé, S. Lysostaphin-coated titan-implants preventing localized osteitis by *Staphylococcus aureus* in a mouse model. *PLoS ONE* **9**, e115940 (2014). 25536060 10.1371/journal.pone.0115940 PMC4275259

[31] Xue, B. et al. A novel controlled-release system for antibacterial enzyme lysostaphin delivery using hydroxyapatite/ chitosan composite bone cement. *PLoS ONE* **9**, e113797 (2014). 25464506 10.1371/journal.pone.0113797 PMC4252040

[32] Johnson, C. T. et al. Hydrogel delivery of lysostaphin eliminates orthopedic implant infection by *Staphylococcus aureus* and supports fracture healing. *Proc. Natl. Acad. Sci. USA* **115**, E4960–E4969 (2018). 29760099 10.1073/pnas.1801013115 PMC5984524

[33] Johnson, C. T. et al. Lysostaphin and BMP-2 co-delivery reduces *S. aureus* infection and regenerates critical-sized segmental bone defects. *Sci. Adv.* **5**, eaaw1228 (2019). No. 31114804 10.1126/sciadv.aaw1228 PMC6524983

[34] Lin, X. et al. Lung-targeting lysostaphin microspheres for methicillin-resistant *Staphylococcus aureus* pneumonia treatment and prevention. *ACS Nano* **15**, 16625–16641 (2021). 34582183 10.1021/acsnano.1c06460

[35] Liu, J. et al. Modular engineering of lysostaphin with significantly improved stability and bioavailability for treating MRSA infections. *Acs. Appl. Mater. Interfaces* **17**, 6703–6715 (2025). 39812685 10.1021/acsami.4c18004

[36] Yang, X. et al. Eradicating intracellular MRSA *via* targeted delivery of lysostaphin and vancomycin with mannose-modified exosomes. *J. Control. Release* **10**, 454–467 (2021). 10.1016/j.jconrel.2020.11.045 33253805

[37] Walsh, S., Shah, A. & Mond, J. Improved pharmacokinetics and reduced antibody reactivity of lysostaphin conjugated to polyethylene glycol. *Antimicrob. Agents Chemother.* **47**, 554–558 (2003). 12543658 10.1128/AAC.47.2.554-558.2003 PMC151727

[38] Urry, D. W. et al. Temperature of polypeptide inverse temperature transition depends on mean residue hydrophobicity. *J. Am. Chem. Soc.* **113**, 4346–4348 (1991).

[39] MacEwan, S. R. & Chilkoti, A. Applications of elastin-like polypeptides in drug delivery. *J. Control. Release* **190**, 314–330 (2014). 24979207 10.1016/j.jconrel.2014.06.028 PMC4167344

[40] Rodríguez-Cabello, J. C., Arias, F. J., Rodrigo, M. A. & Girotti, A. Elastin-like polypeptides in drug delivery. *Adv. Drug Deliv. Rev.* **97**, 85–100 (2016). 26705126 10.1016/j.addr.2015.12.007

[41] Gong, L., Yang, Z., Zhang, F. & Gao, W. Cytokine conjugates to elastin-like polypeptides. *Adv. Drug Deliv. Rev.* **190**, 114541 (2022). 36126792 10.1016/j.addr.2022.114541

[42] Hu, J., Wang, G., Liu, X. & Gao, W. Enhancing pharmacokinetics, tumor accumulation, and antitumor efficacy by elastin-like polypeptide fusion of interferon alpha. *Adv. Mater.* **27**, 7320–7324 (2015). 26463662 10.1002/adma.201503440

[43] Wang, Z. et al. One-month zero-order sustained release and tumor eradication after a single subcutaneous injection of interferon alpha fused with a body-temperature-responsive polypeptide. *Biomater. Sci.* **7**, 104–112 (2019). 10.1039/c8bm01096j 30515491

[44] Zhang, S., Sun, Y., Zhang, L., Zhang, F. & Gao, W. Thermoresponsive polypeptide fused L-asparaginase with mitigated immunogenicity and enhanced efficacy in treating hematologic malignancies. *Adv. Sci.* **10**, 1–13 (2023). 10.1002/advs.202300469 PMC10427413 37271878

[45] Yang, Z. et al. A superlong-acting growth hormone-polypeptide fusion for growth hormone deficiency treatment. *Adv. Healthc. Mater.* **13**, 1–11 (2024). 10.1002/adhm.202302507 38030143

[46] Liang, P. et al. Spatiotemporal combination of thermosensitive polypeptide fused interferon and temozolomide for post-surgical glioblastoma immunochemotherapy. *Biomaterials* **264**, 120447 (2021). 33069137 10.1016/j.biomaterials.2020.120447

[47] Li, Y. et al. Synergic chemoimmunotherapy of cervical cancer by combining cisplatin with thermosensitive polypeptide fused interferon alpha. *Adv. Ther.* **7**, 2400098 (2024).

[48] Wang, Z. et al. Thermoresponsive and protease-cleavable interferon-polypeptide conjugates with spatiotemporally programmed two-step release kinetics for tumor therapy. *Adv. Sci.* **6**, 1900586 (2019). 10.1002/advs.201900586 PMC6702759 31453069

[49] Zhang, S., Gong, L., Sun, Y., Zhang, F. & Gao, W. An ultra-long-acting L-asparaginase synergizes with an immune checkpoint inhibitor in starvation-immunotherapy of metastatic solid tumors. *Biomaterials* **312**, 122740 (2025). 39096839 10.1016/j.biomaterials.2024.122740

[50] Gong, L. et al. Thermo-pH-sensitive polypeptides boost protein drug’s tumor permeation and pharmacology. *Small* **21**, 2501787 (2025). 10.1002/smll.202501787 40143732

[51] Yao, Q., Zhang, F., Yang, Z., Sun, Y. & Gao, W. A super-long-acting anti-PD-L1 nanobody fused to elastin-like polypeptide for triple-negative breast cancer immunotherapy. *J. Control. Release* **385**, 114002 (2025). 40614868 10.1016/j.jconrel.2025.114002

[52] Zhang, F. et al. Trichosanthin fused to thermo-pH-sensitive polypeptides for synergistic cancer immunotherapy. *ACS Appl. Mater. Interfaces* **17**, 50349–50363 (2025). 40875969 10.1021/acsami.5c11256

[53] Wang, Z., Guo, J., Liu, X., Sun, J. & Gao, W. Temperature-triggered micellization of interferon alpha-diblock copolypeptide conjugate with enhanced stability and pharmacology. *J. Control. Release* **328**, 444–453 (2020). 32898593 10.1016/j.jconrel.2020.08.065

[54] Gonzalez-Delgado, L. S. et al. Two-site recognition of *Staphylococcus aureus* peptidoglycan by lysostaphin SH3b. *Nat. Chem. Biol.* **16**, 24–30 (2020). 31686030 10.1038/s41589-019-0393-4 PMC6920042

[55] Xiao, Y. et al. A covalent peptide-based lysosome-targeting protein degradation platform for cancer immunotherapy. *Nat. Commun.* **16**, 138 (2025). 39910101 10.1038/s41467-025-56648-6 PMC11799215

[56] Zhang, Y. et al. Mitochondrial transplantation augments the reparative capacity of macrophages following myocardial injury. *Adv. Sci.* **12**, e06337 (2025). 10.1002/advs.202506337 PMC12622452 40827661

