# Supplementary material for: Eradication of MRSA biofilm-associated implant infections by low-immunogenic sustained-release lysostaphin fused to thermosensitive polypeptides
Source: Nat Commun. 2026 Aug 14;17:10108. doi: 10.1038/s41467-026-76664-4 (PMC13598132; doi:10.1038/s41467-026-76664-4)
Supplement: Supplementary file 1 [file 41467_2026_76664_MOESM1_ESM.pdf]

Supplementary Information

Eradication of MRSA biofilm-associated implant infections by low-immunogenic sustained-release lysostaphin fused to thermosensitive polypeptides

Guoqing Fan[1,4,5], Hao Wang[6], Yuxin Gong[1,4,5], Si Liu[2], Yuanzi Sun[1,4,5], Jie Tian[7], Hongbin Wang[6], Litao Zhang[1,4,5], Fan Zhang[1,4,5], Like Gong[1,4,5], Lin Wang[8], Fengmin Lu[8], Yongping Cao[6], Wanliang Lu[4,], Mingming Xu[3,*], Shengtao Zhu[2,*], Weiping Gao[1,4,5,*]*

[1]Beijing Advanced Center of Cellular Homeostasis and Aging-Related Diseases, Department of Biomedical Engineering, Institute of Advanced Clinical Medicine, Peking University, Beijing 100191, China

[2]Department of Gastroenterology, Beijing Friendship Hospital, Capital Medical University, State Key Laboratory of Digestive Health, National Clinical Research Center for Digestive Disease, Beijing 100050, China

[3]Department of Geriatric Dentistry, Peking University School and Hospital of Stomatology, & National Center for Stomatology & National Clinical Research Center for Oral Diseases & National Engineering Research Center of Oral Biomaterials and Digital Medical Devices, Beijing 100081, China.

[4]State Key Laboratory of Natural and Biomimetic Drugs, Beijing Key Laboratory of Advanced Pharmaceutical Preparation, School of Pharmaceutical Sciences, Peking University, Beijing 100191, China

[5]Peking University-Yunnan Baiyao International Medical Research Center, Peking University International Cancer Institute, Beijing 100191, China

[6]Department of Orthopedics, Peking University First Hospital, Beijing 100034, China

[7]Department of Dermatology and Venereology, Peking University First Hospital, Beijing 100034, China

[8]Department of Microbiology & Infectious Disease Center, School of Basic Medical Sciences, Peking University, Beijing, 100191, China

[*]Corresponding author. E-mail: gaoweiping@hsc.pku.edu.cn (W.P.G.); zhushengtao@ccmu.edu.cn(S.T.Z.); xumingming@pkuss.bjmu.edu.cn (M.M.X.); luwl@bjmu.edu.cn (W.L.L.)

G. Q. Fan, H. Wang, Y.X. Gong and S. Liu contributed equally to this work.

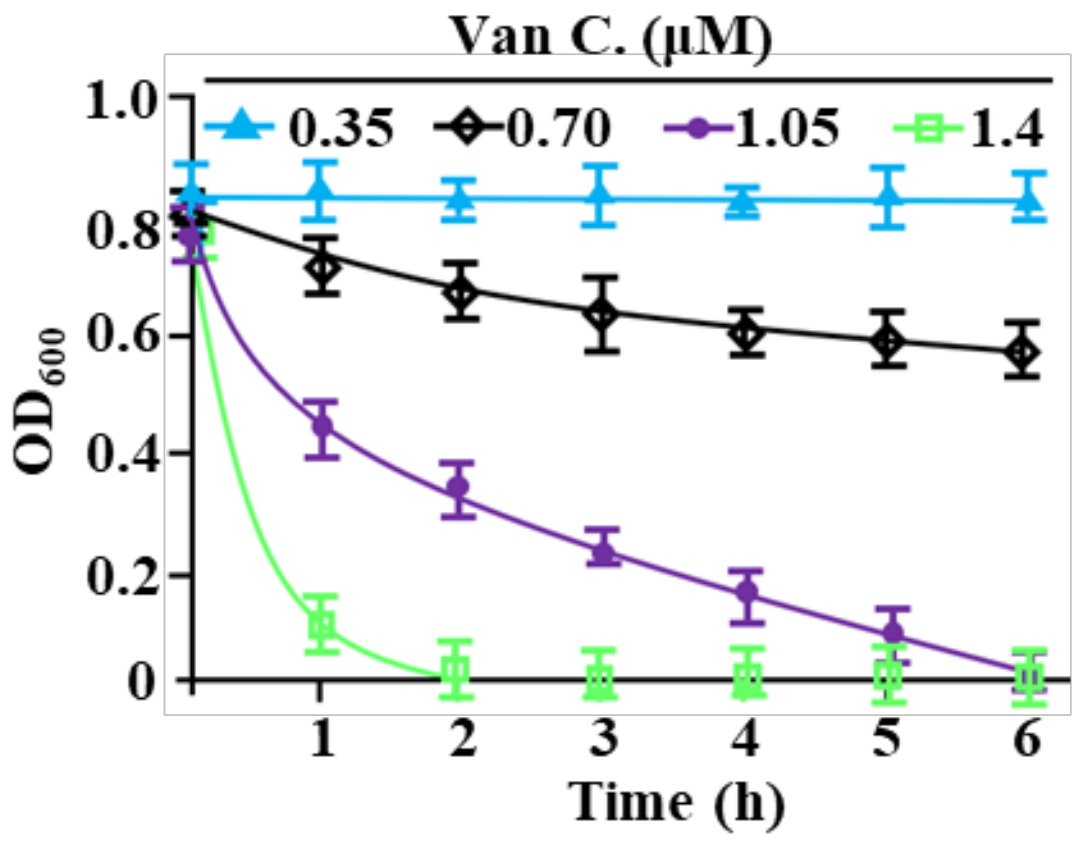

Supplementary Fig. 1 | Turbidity-time curves of planktonic MRSA treated with Van at given concentrations. OD_{600} denotes optical density at 600 nm. All data are expressed as the mean ± standard deviation (SD) (n = 3). Biomass-time curves of MRSA biofilms treated with vancomycin; three independent experiments were made for each concentration. Source data are provided as a Source Data file.

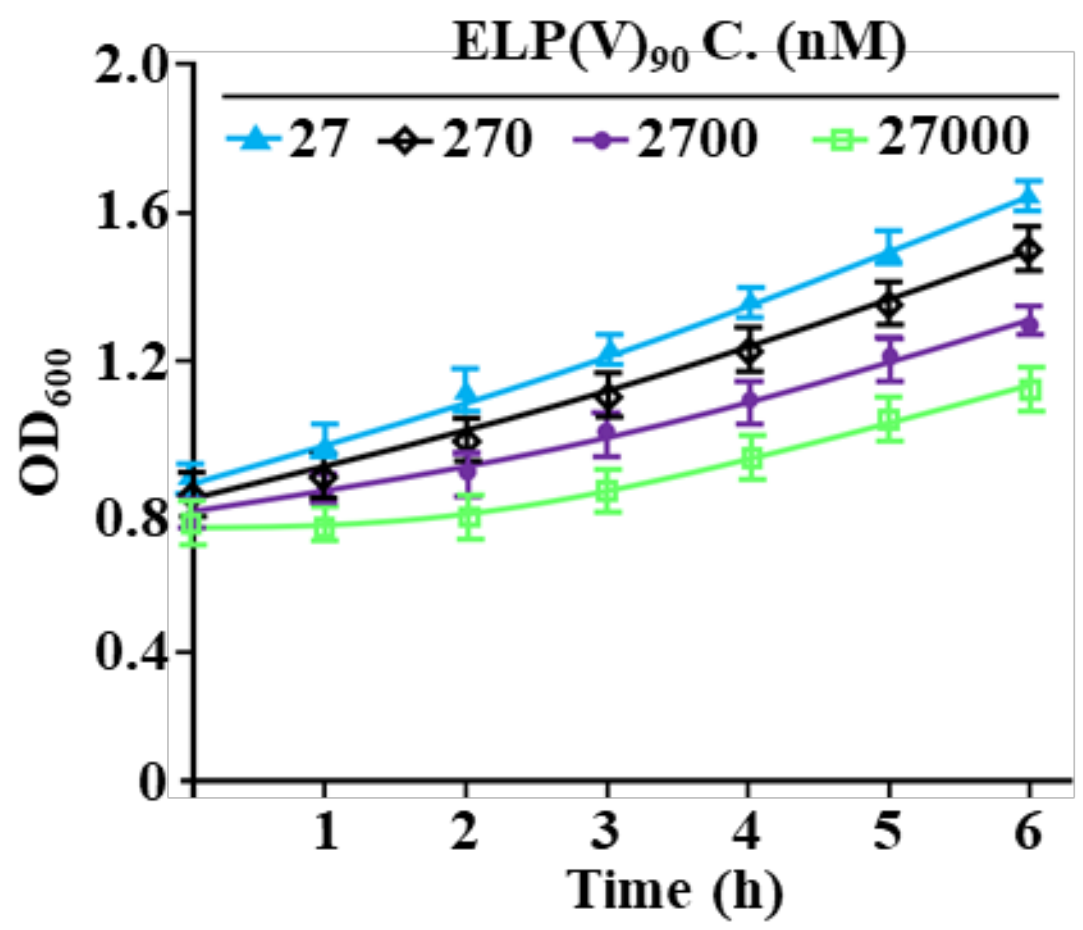

Supplementary Fig. 2 | Turbidity-time curves of planktonic MRSA treated with ELP(V)$_{90}$ at given concentrations. OD$_{600}$ denotes optical density at 600 nm. All data are expressed as the mean ± standard deviation (SD) (n = 3). Biomass-time curves of MRSA biofilms treated with vancomycin; three independent experiments were made for each concentration. Source data are provided as a Source Data file.

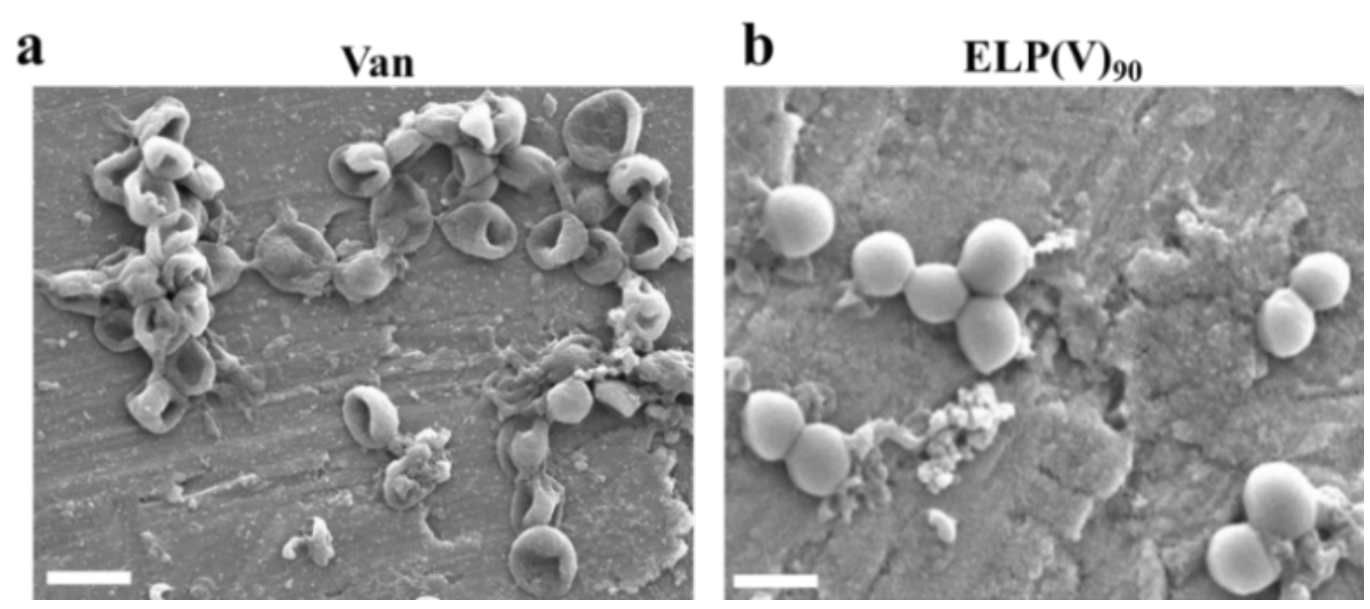

Supplementary Fig. 3 | SEM images of planktonic MRSA treated with Van at 1.5 μM and ELP(V)$_{90}$ at 3 nM. a, Cells of planktonic MRSA treated by Van with the concentration of 1.5 μM. b, Cells of planktonic MRSA treated by ELP(V)$_{90}$ with the concentration of 3 nM. Each sample was analyzed in triplicate (biological replicates); bacterial morphology remained uniform across all replicates and independent microscopic fields of view. Scale bars, 1μm. Source data are provided as a Source Data file.

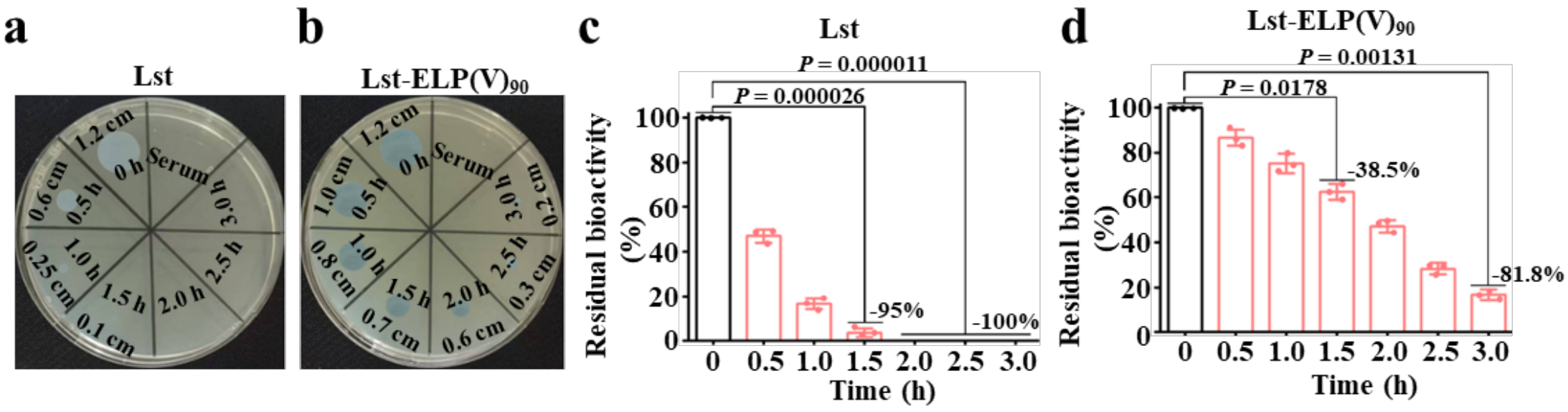

Supplementary Fig. 4 | Improved thermal stability of Lst-ELP(V)$_{90}$ over Lst at the soluble state in serum. a, b, Evaluation of the antibacterial activity of Lst and Lst-ELP(V)$_{90}$ at 5 μM in serum after incubation at 37 °C for given times via the zone of inhibition assay. **c, d,** Quantification of the residual activity of Lst and Lst-ELP(V)$_{90}$ from Supplementary Fig. 4a, b. The statistical significance differences were determined by one-way single factorial analysis of variance (ANOVA) and data were presented as the mean ± standard deviation (SD) (n = 3); **(P < 0.01) and ****(P < 0.0001), ns denotes no significance. Three independent experimental runs were performed at separate time points. P values were determined by unpaired two-tailed t-tests. Source data are provided as a Source Data file.

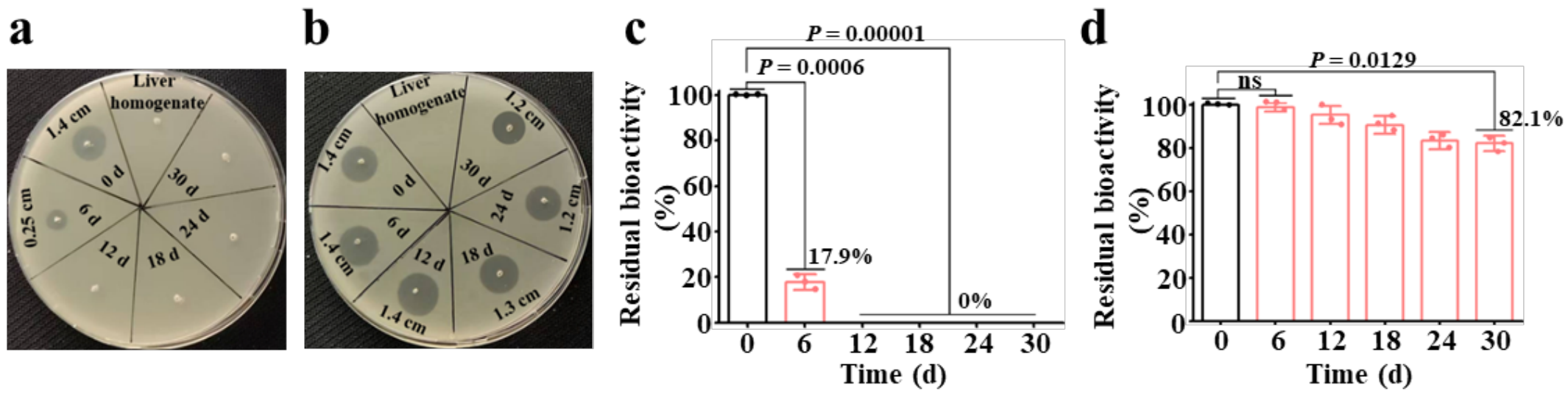

Supplementary Fig. 5 | Evaluation of the stability of Lst and Lst-ELP(V)$_{90}$ in liver homogenate. a, b, Changes in inhibition zone size of Lst (left) and Lst-ELP(V)$_{90}$ (right) at different times treated by the liver homogenate. **c, d,** Quantification of the residual bioactivity of Lst and Lst-ELP(V)$_{90}$, respectively. The statistical significance differences were determined by one-way single factorial analysis of variance (ANOVA) and data were presented as the mean ± standard deviation (SD) (n = 3); **(P < 0.01) and ****(P < 0.0001), ns denotes no significance. P values were determined by unpaired two-tailed t-tests. Source data are provided as a Source Data file.

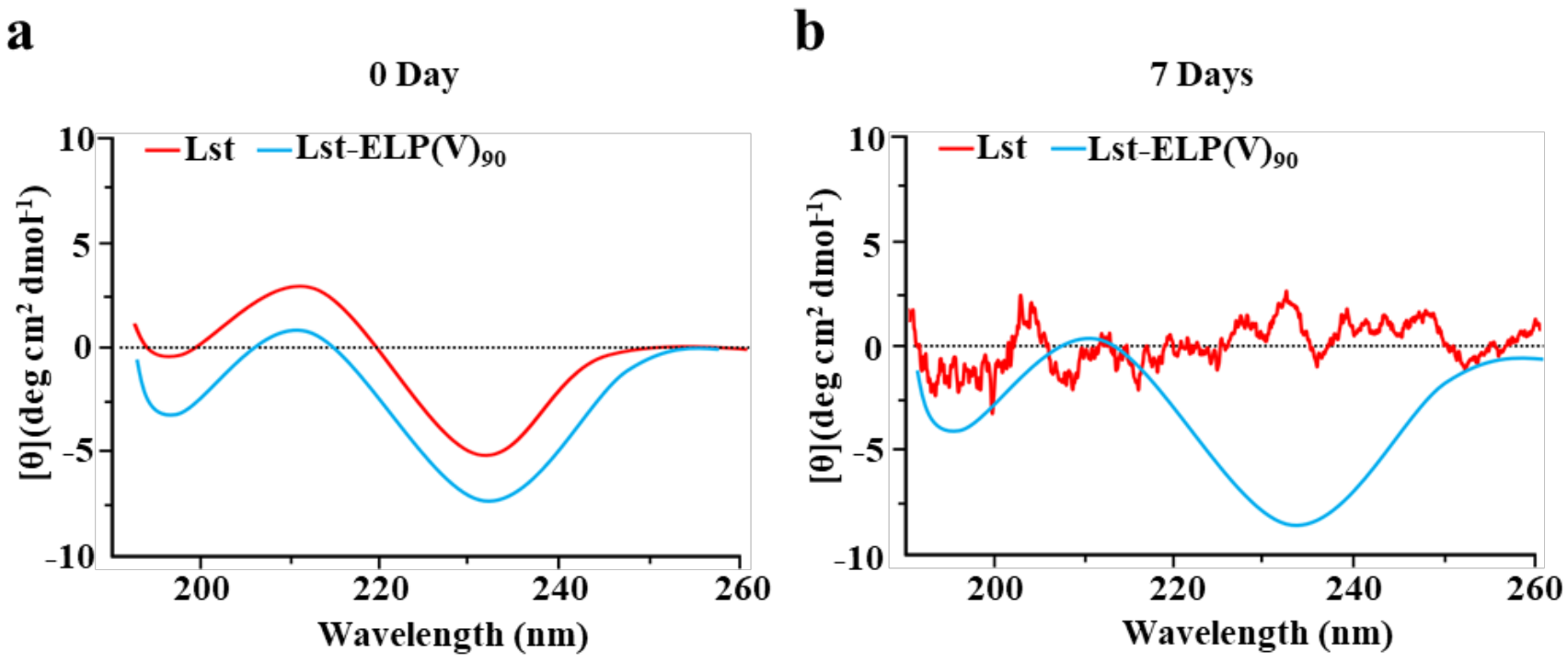

Supplementary Fig. 6 | CD spectra for Lst and Lst-ELP(V)$_{90}$ storage in PBS at 0 and 7 days. **a, b,** Changes in CD spectra of Lst and Lst-ELP(V)$_{90}$ on day 0 and 7 respectively. Three independent experimental runs were performed at separate time points. Source data are provided as a Source Data file.

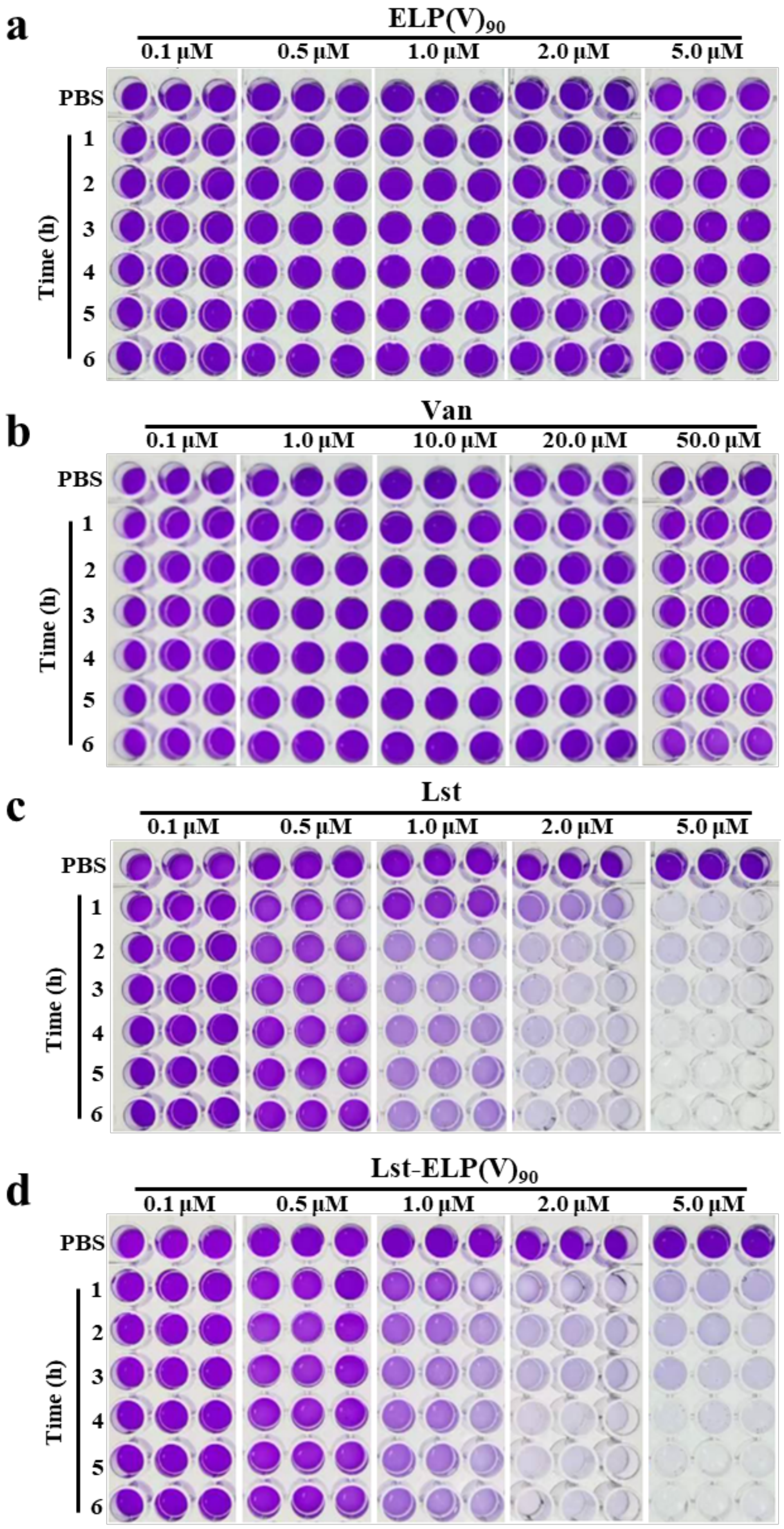

Supplementary Fig. 7 | Crystal violet staining of MRSA biofilms treated with ELP(V)$_{90}$, Van, Lst, and Lst-ELP(V)$_{90}$ respectively at given concentrations at 37 °C for specified times. Three independent experimental runs were performed at separate time points. Source data are provided as a Source Data file.

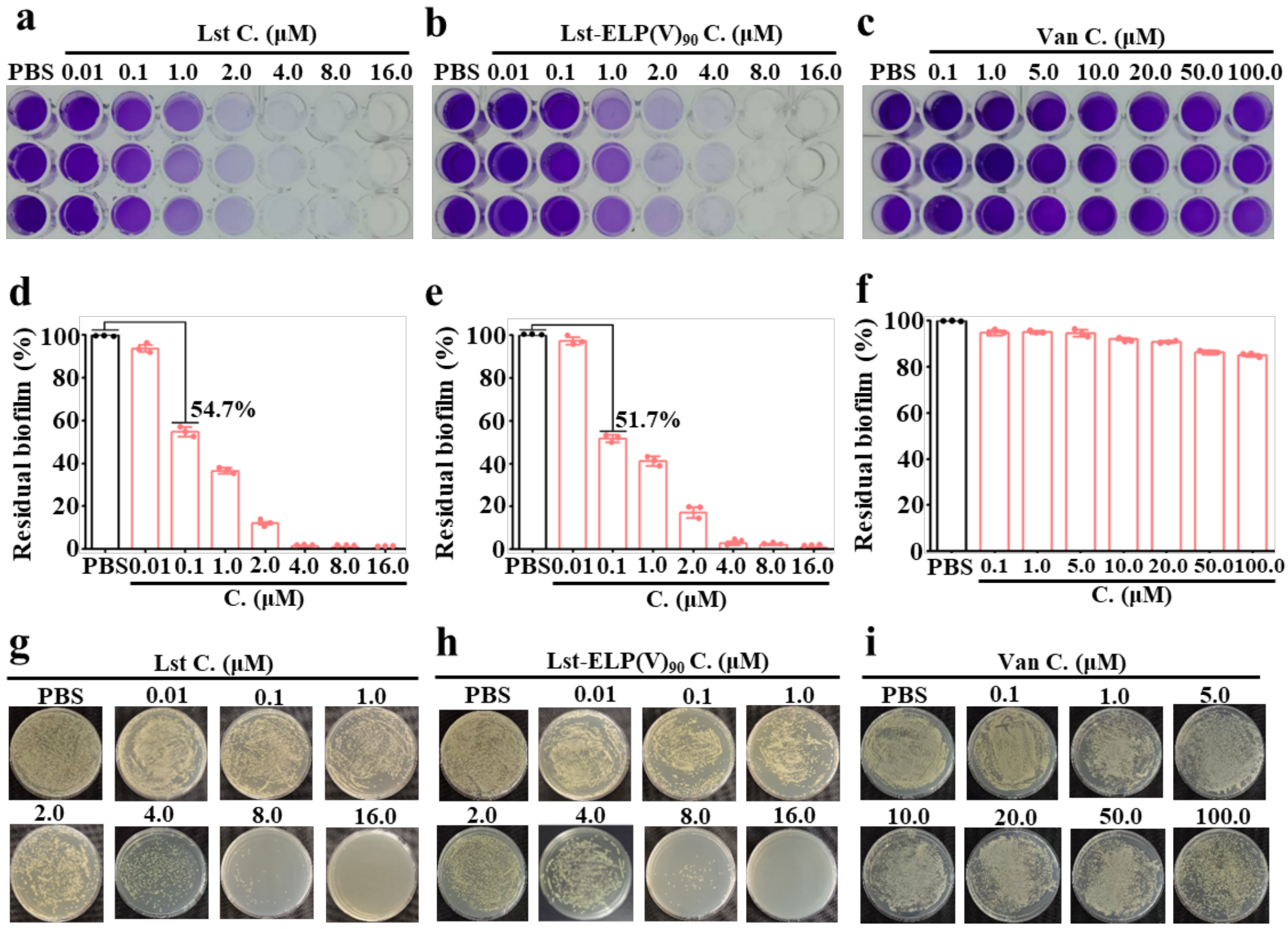

Supplementary Fig. 8 | MBIC and MBEC values determined for Lst, Lst-ELP(V)$_{90}$ and Van. **a-c,** Crystal violet staining of MRSA biofilms treated with Lst, Lst-ELP(V)$_{90}$ and Vancomycin at given concentrations. **d-f,** Minimum biofilm inhibitory concentration (MBIC) quantified for Lst, Lst-ELP(V)$_{90}$ and Vancomycin respectively. **g-i,** Minimum biofilm eradication concentration (MBEC) quantified for Lst, Lst-ELP(V)$_{90}$ and Vancomycin through CFU assay respectively. Three independent experimental runs were performed at separate time points. The data are presented as the mean ± SD. Source data are provided as a Source Data file.

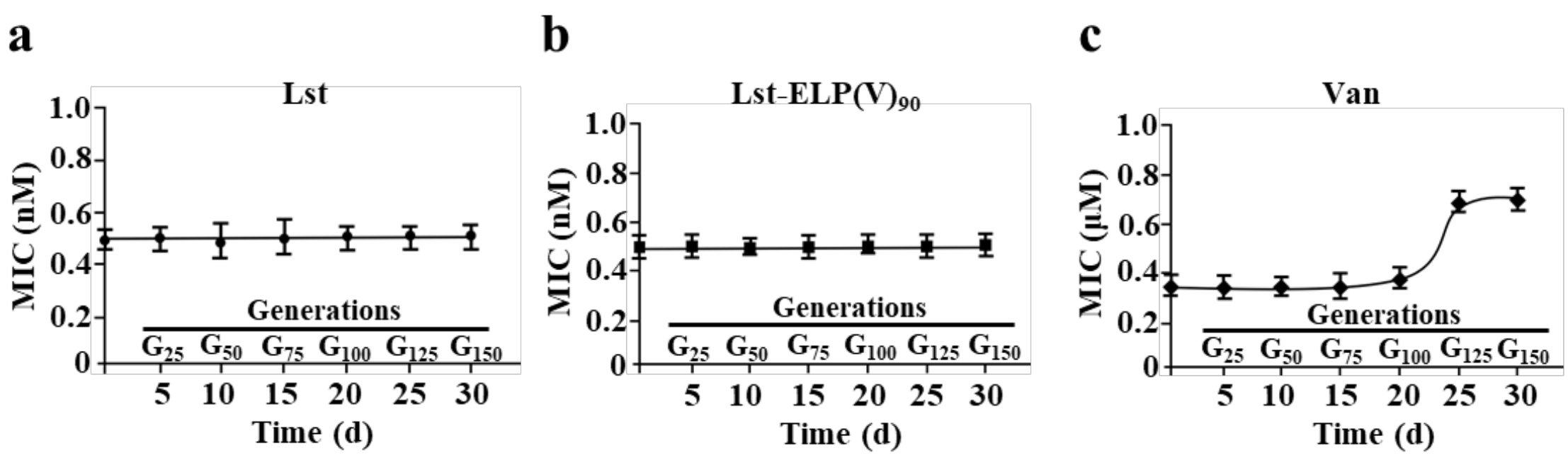

Supplementary Fig. 9 | Resistance tested for Lst, Lst-ELP(V)$_{90}$ and Van against the MRSA. a-c, MIC values tested results of Lst, Lst-ELP(V)$_{90}$ and Van against MRSA over 150 generations of

continuous passage respectively. Three independent experimental runs were performed at separate time points. The data are presented as the mean ± SD. Source data are provided as a Source Data file.

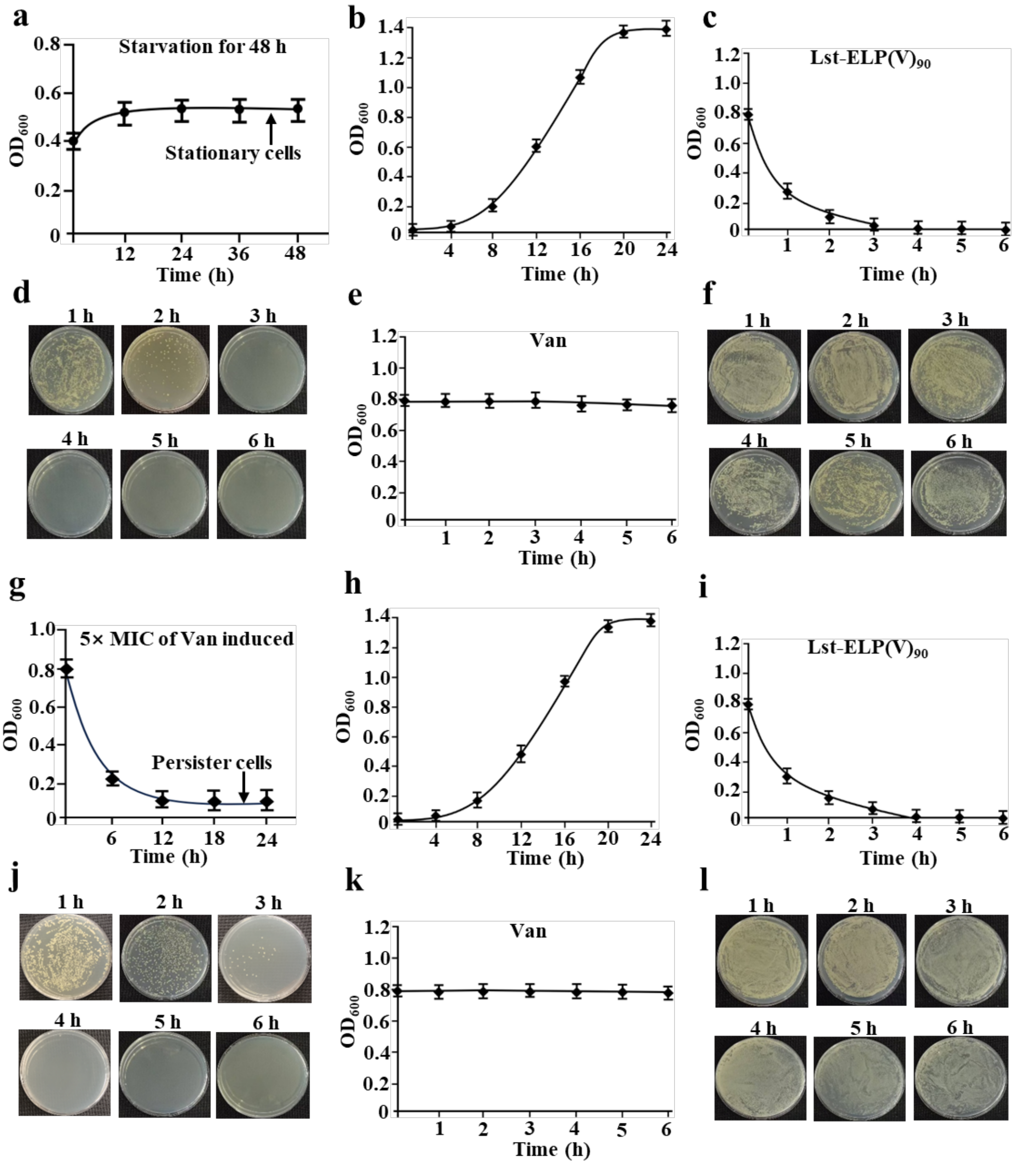

Supplementary Fig. 10 | Antibacterial activity of Lst-ELP(V)$_{90}$ and Van against stationary-phase and persister MRSA cells. a, Establishment of starvation-induced stationary-phase MRSA models. **b,** Growth curves of MRSA cultured from stationary-phase bacterial inoculum. **c,** Time-killing kinetics of stationary-phase MRSA upon Lst-ELP(V)$_{90}$ treatment. **d,** Quantitative CFU analysis of stationary-phase MRSA after Lst-ELP(V)$_{90}$ treatment for different durations. **e,** Time-

killing kinetics of stationary-phase MRSA following Van intervention. **f,** Quantitative CFU statistics of stationary-phase MRSA after Van treatment at different time points. **g,** Induction of MRSA persister cells by high-dose antibiotics. **h,** Growth curves of MRSA derived from persister cell inoculum. **i,** Time-killing kinetics of persister MRSA upon Lst-ELP(V)$_{90}$ treatment. **j,** Quantitative CFU analysis of persister MRSA after Lst-ELP(V)$_{90}$ treatment for different durations. **k,** Time-killing kinetics of persister MRSA under Van treatment. **l,** CFU quantification of persister MRSA following Van treatment at different time points. Three independent experimental runs were performed at separate time points. The data are presented as the mean ± SD. Source data are provided as a Source Data file.

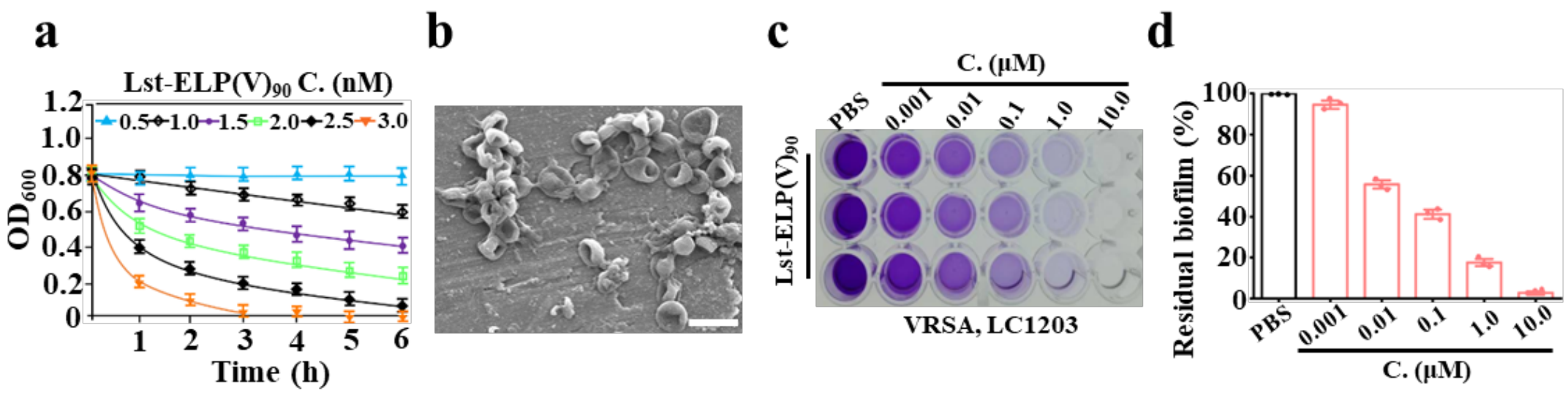

Supplementary Fig. 11 | Bioactivities tested for Lst-ELP(V)$_{90}$ against the VRSA planktonic bacteria and its biofilm. a, Time-killing curve of the VRSA planktonic cells under different concentrations of Lst-ELP(V)$_{90}$ treated. **b,** SEM observed results of the VRSA planktonic cells treated by the Lst-ELP(V)$_{90}$ under the MBC values. Scale bars:1µm. **c,** Crystal violet staining of VRSA biofilm treated by the Lst-ELP(V)$_{90}$ at given concentrations. **d,** Residual biofilm rates quantified for gradient concentrations of Lst-ELP(V)$_{90}$ against the VRSA biofilm. Three independent experimental runs were performed at separate time points. The data are presented as the mean ± SD. Source data are provided as a Source Data file.

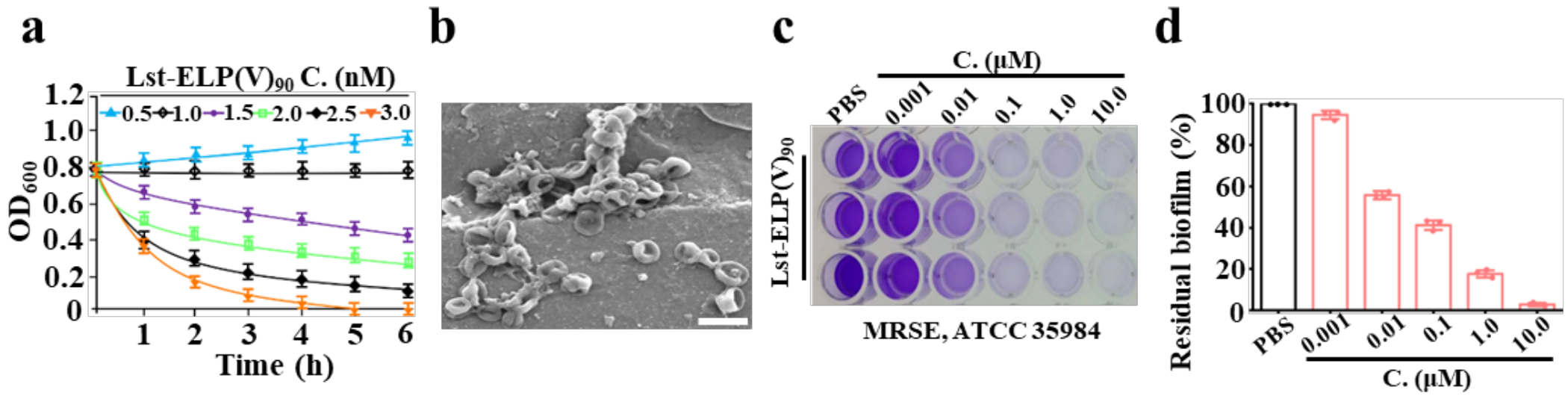

Supplementary Fig. 12 | The bactericidal activity of Lst-ELP(V)$_{90}$ against planktonic MRSE and its efficacy in eradicating MRSE biofilms. a, MIC and MBC values tested results of Lst-ELP(V)$_{90}$ against MRSE planktonic cells. **b,** Efficacy of Lst-ELP(V)$_{90}$ in eradicating MRSE biofilms across a gradient of concentrations by crystal violet staining. **c,** The biofilm eradication efficacy of Lst-ELP(V)$_{90}$ against MRSE was quantified across a range of concentrations. Three independent experimental runs were performed at separate time points. The data are presented as the mean ± SD. Source data are provided as a Source Data file.

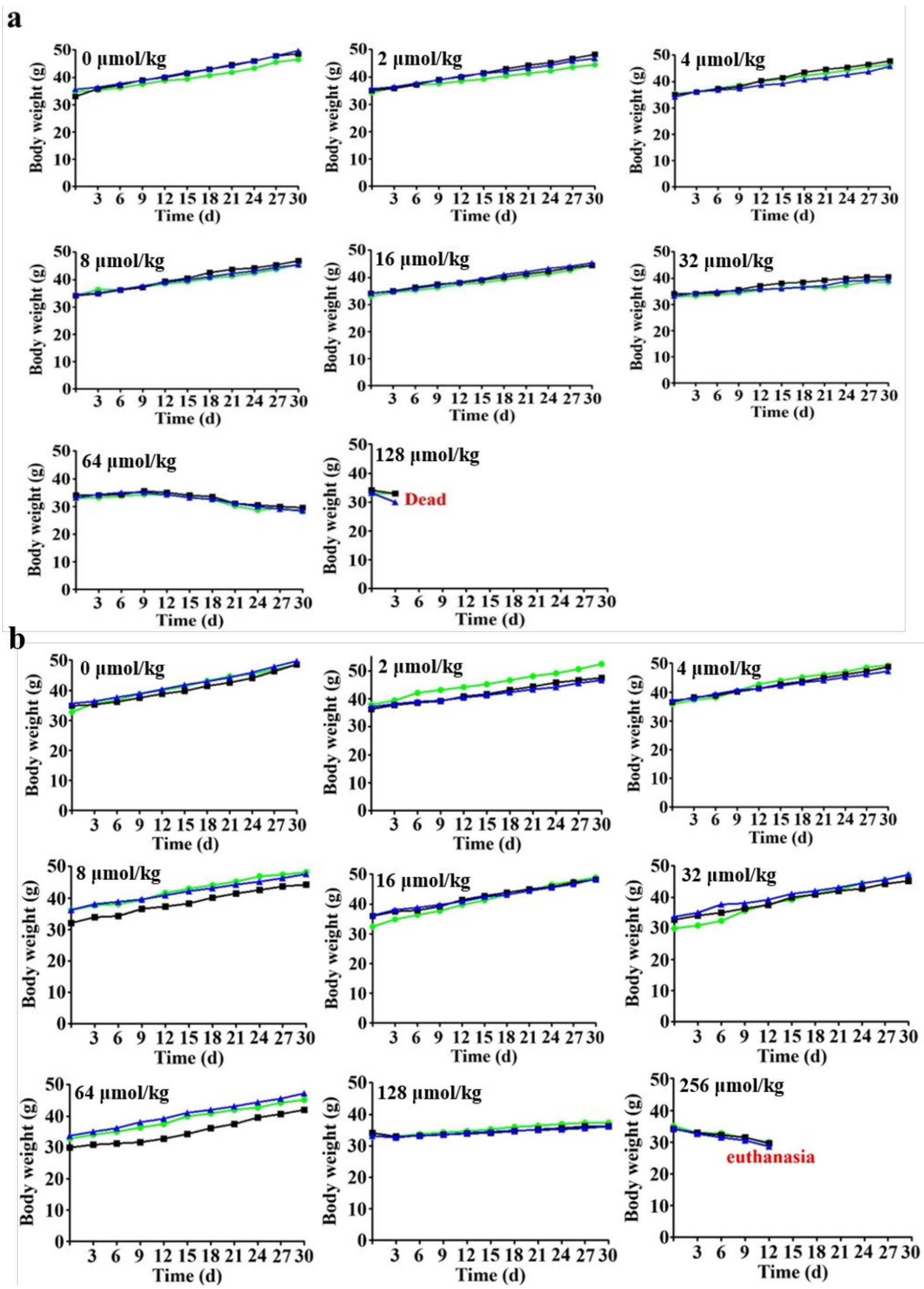

Supplementary Fig. 13 | Determination of MTDs of Lst (a) and Lst-ELP(V)$_{90}$ (b) in mice(n = 3 mice per group)by dose escalation. Three independent experimental runs were performed at separate time points. Source data are provided as a Source Data file.

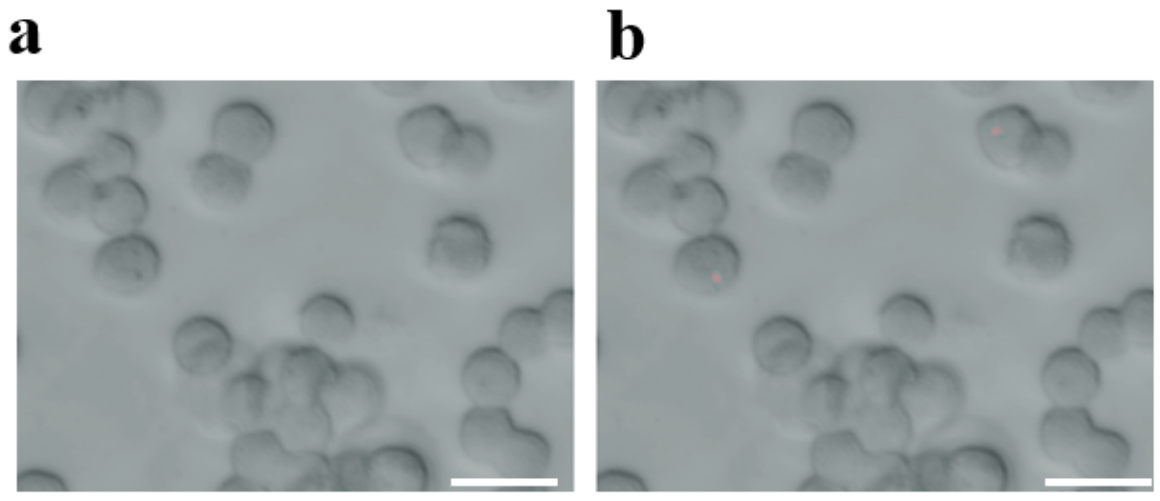

Supplementary Fig. 14 | CLSM observation of the phagocytosis of Cy5-labeled ELP(V)$_{90}$ by macrophages. a, Observation the Cy5 labeled ELP(V)$_{90}$ uptake by macrophage under the bright field. Scale bar:200 μm. **b,** Observation results for Cy5 labeled ELP(V)$_{90}$ uptake by macrophage under the lasering field with wavelength of 650 nm. Scale bars: 200 μm. The experiment was repeated three times independently, yielding consistent visual field observations across all trials. Source data are provided as a Source Data file.

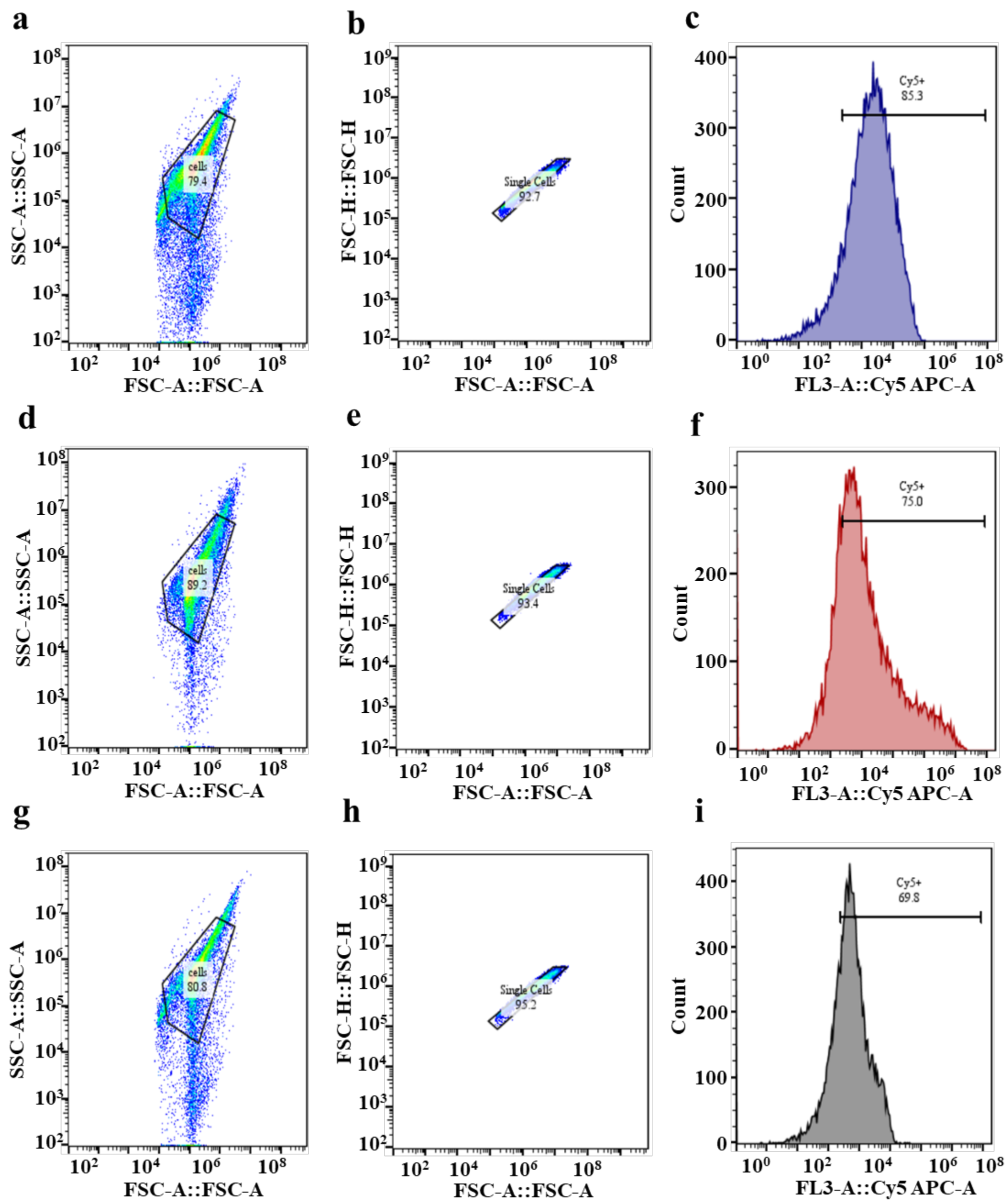

Supplementary Fig. 15 | Representative flow cytometry gating strategy for Cy5 uptake analysis. Cells were first gated based on forward scatter (FSC) and side scatter (SSC), followed by singlet gating using FSC-A versus FSC-H. Cy5 fluorescence intensity was then quantified from the single-cell population. The same gating strategy was applied to all samples. a–c, Cy5 uptake analysis of individual Lst proteins. **d–f,** Cy5 uptake analysis of fused Lst-ELP(V)$_{90}$ proteins. **g–i,** Cy5 uptake analysis of individual ELP(V)$_{90}$ proteins. Source data are provided as a Source Data file.

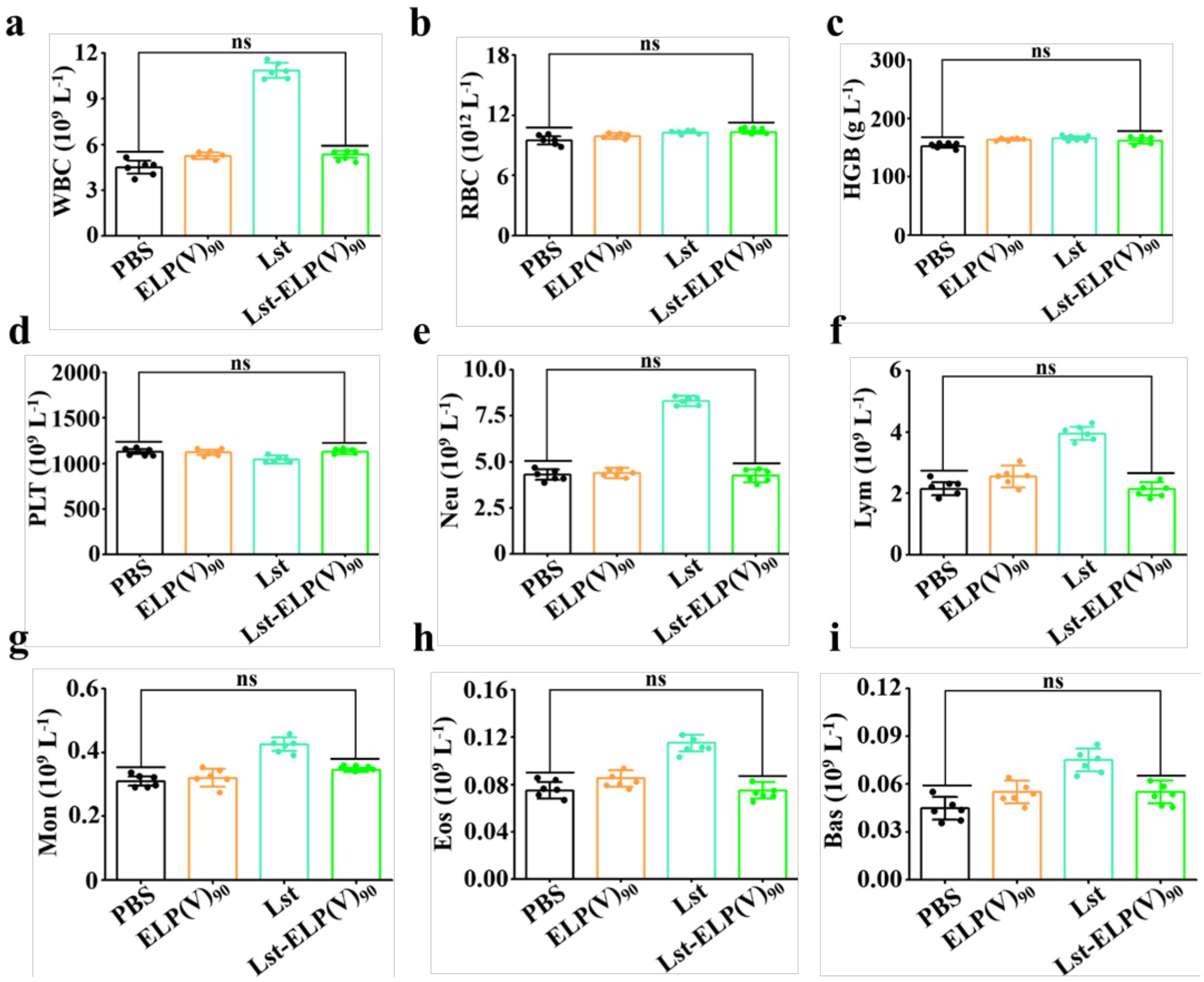

Supplementary Fig. 16 | Blood sample analysis for Lst and Lst-ELP(V)$_{90}$ biosafety assessment (n = 6). The data are presented as the mean ± SD. *(P < 0.05), **(P < 0.01), ns denotes no significance. WBC, white blood cell; RBC, red blood cell; HGB, hemoglobin; PLT, platelet; Neu, neutrophil; Lym, lymphocyte; Mon, monocytes; Eos, eosinophils; Bas, basophil. The experiment was performed in triplicate at separate times. Source data are provided as a Source Data file.

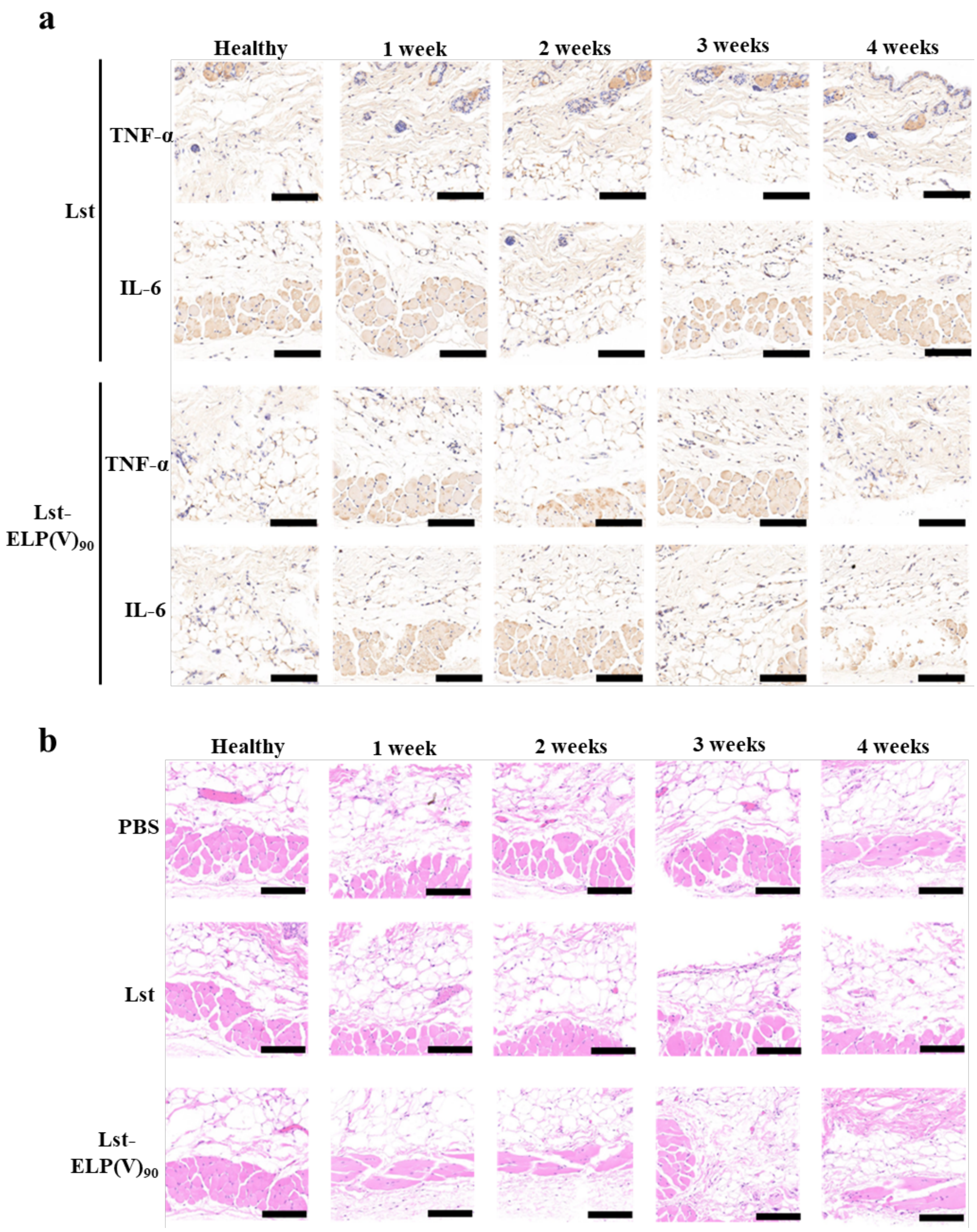

Supplementary Fig. 17 | Depot-related inflammatory responses induced by Lst-ELP(V)$_{90}$ fusion protein and free Lst at local injection sites(n=6). a, Immunohistochemical staining analyzed for the mouse skin tissues at injection sites. Scale bar: 100 μm. **b,** H&E staining results for mouse skin tissues at injection sites. Scale bar: 100 μm. Three independent experiment was performed at separate times. Source data are provided as a Source Data file.

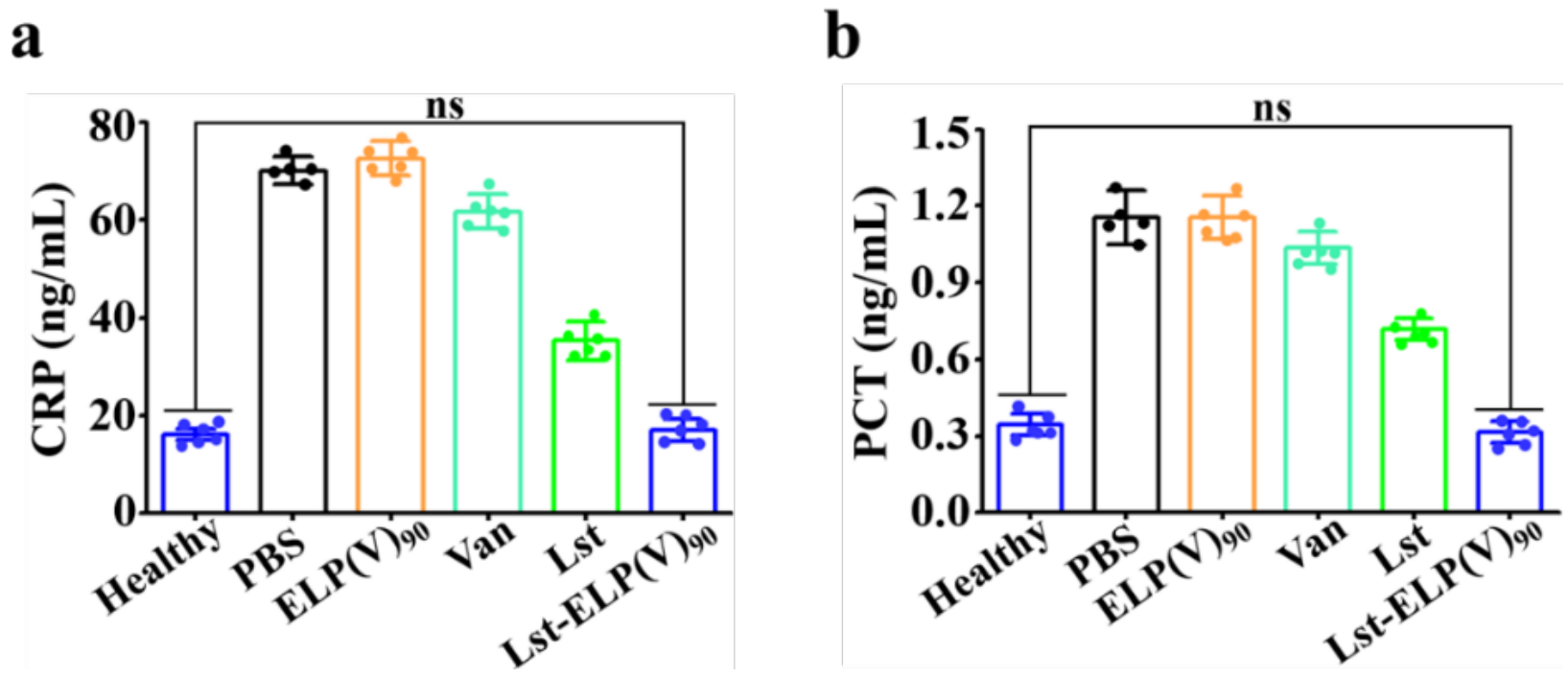

Supplementary Fig. 18 | The levels of CRP (a) and PCT (b) at 28 d after varying treatments. Both the healthy and Lst-ELP(V)$_{90}$ groups were highlighted in blue (n = 6). The data are presented as the mean ± SD. **(*P* < 0.01), ***(*P* < 0.001) and ****(*P* < 0.0001), ns denotes no significance. Source data are provided as a Source Data file.

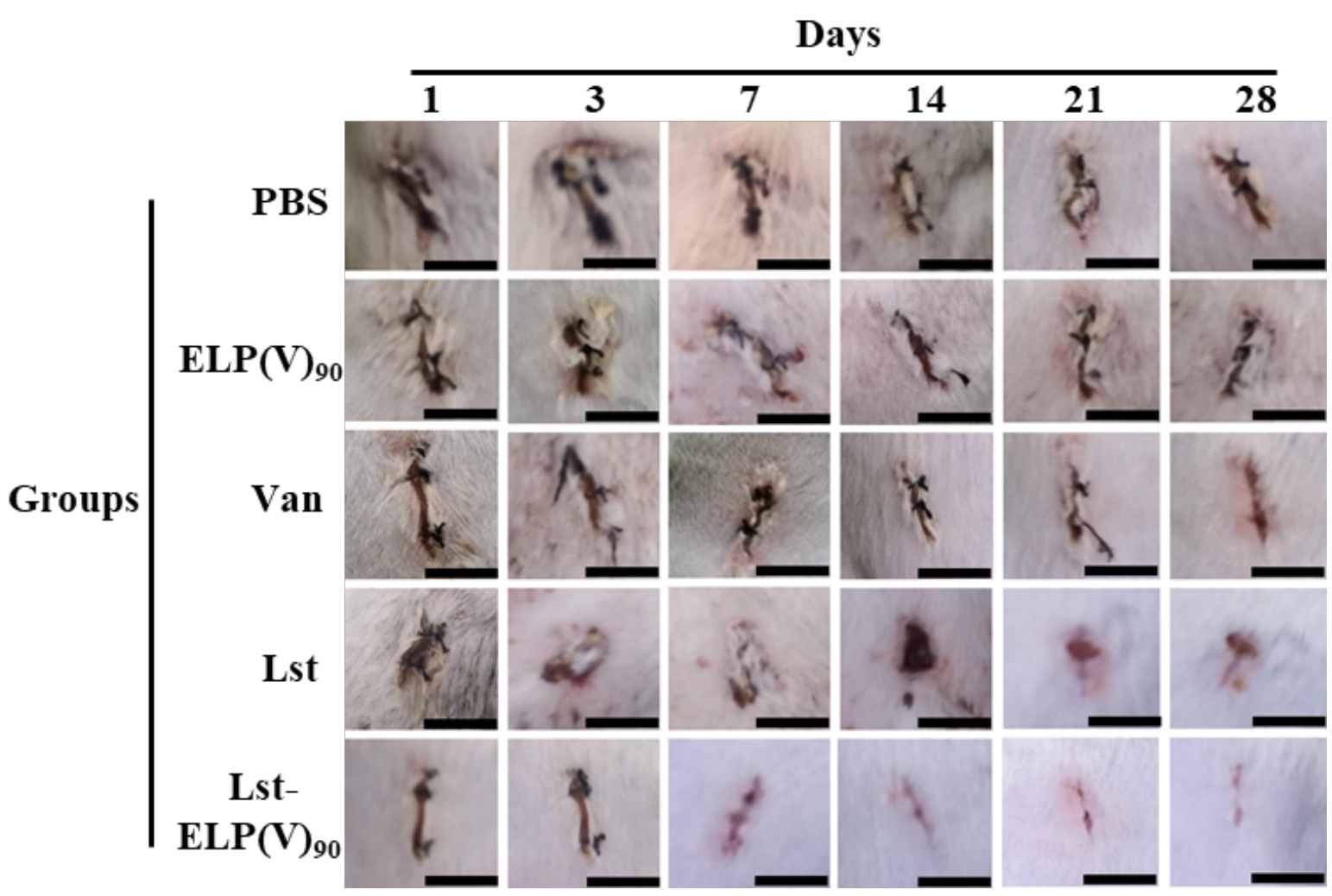

Supplementary Fig. 19 | Photographs of the process of skin wound healing after varying treatments in different times. Scale bar: 1 cm. Source data are provided as a Source Data file.

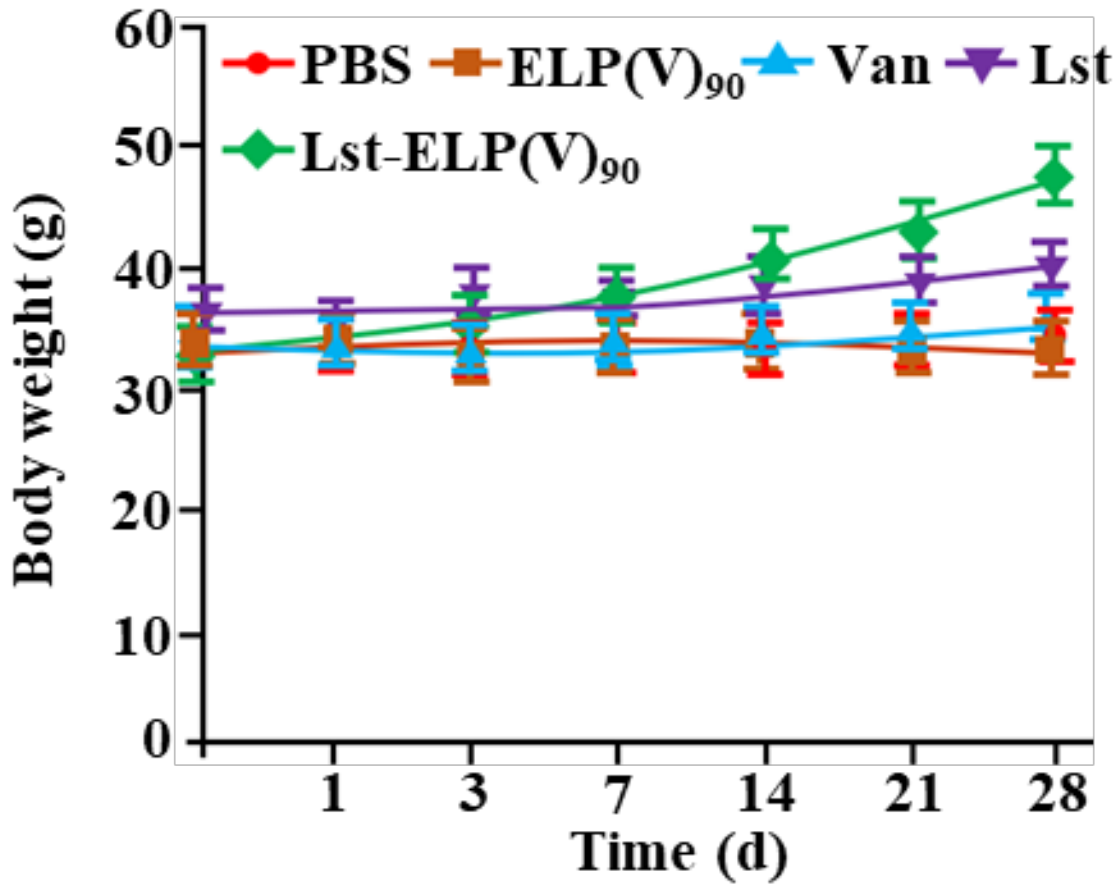

Supplementary Fig. 20 | Weight changes of mice in each group during the treatment process (n = 6). The data are presented as the mean ± SD. Source data are provided as a Source Data file.

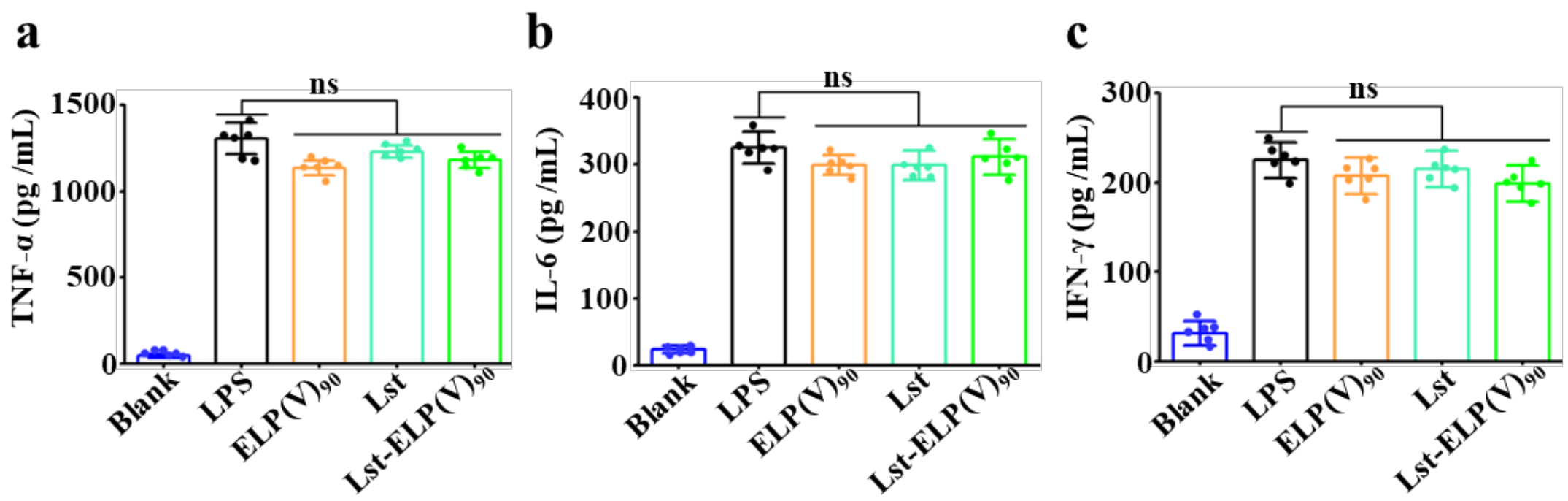

Supplementary Fig. 21 | Immunomodulatory effects of $ELP(V)_{90}$, Lst and Lst-$ELP(V)_{90}$. a–c, Secretion levels of pro-inflammatory cytokines TNF-α, IL-6 and IFN-γ in macrophages under different treatments. The Blank group consisted of macrophages cultured with only medium to determine the basal inflammatory cytokine release. The LPS group was macrophages stimulated with LPS alone. The $ELP(V)_{90}$, Lst and Lst-$ELP(V)_{90}$ groups referred to LPS-stimulated macrophages separately treated with $ELP(V)_{90}$, Lst and Lst-$ELP(V)_{90}$, respectively. The data are presented as the mean ± SD (n = 6), ns denotes no significance. Source data are provided as a Source Data file.

Supplementary Table 1. Pharmacokinetic parameters of Lst and Lst-$ELP(V)_{90}$

Objects	Lst (30 μmol kg^{-1})	Lst-ELP(V)$_{90}$ (30 μmol kg^{-1})
Half-life[h][a)]	4.21±0.03	487.63±13.03
$AUC_{0-\infty}$ [μg mL^{-1} h^{-1}] [b)]	6.12±1.35	285.23±9.24
C_{max} [μg mL^{-1}][c)]	5.58±1.08	22.32±0.73
T_{max} [h][d)]	7.19±0.02	210.00±31.26

a) The plasma half-life. [b)] The area under the curve. [c)] The plasma peak concentration. [d)] The time after administration of the drug at which C_{max} was observed. Data are shown as mean ± SEM (*n* = 6).

Supplementary Table 2. The strains, cells and animal used in this study.

Objects	Sub-strain/species	Source
Escherichia. coli	BL21(DE3)	WEIDI Biotech, China
Escherichia. coli	Top10	WEIDI Biotech, China
Methicillin-resistant *Staphylococcus aureus*	ATCC 33591	ATCC, USA
Methicillin-resistant *Staphylococcus epidermidis*	ATCC 35984	ATCC, USA
Vancomycin-resistant *Staphylococcus aureus*	LC1203	Qianfoshan Hospital, China
Mouse embryonic osteoblast cell	MC3T3-E1	Procell Life Science & Technology, China
Murine macrophage	RAW264.7	Procell Life Science & Technology, China
Mice	Kunming (male,6 weeks)	Beijing Vital River, China

Supplementary Table 3. The primers used in this study.

Primer name	DNA sequence (5'-3')
24a-F	GGCTACGGTCTCTACATAGCGCGCAGTGGCT
24a-R	GGCTACGGTCTCCAATGGTGCCCCACAGCAC
Lst-F	GGCTACGGTCTCCCATTAAACATCACCATCACCACCATG
Lst-R	GGCTACGGTCTCTATGTTCATGGGTCGCCGCTTTAATGGTGCCCCAC AG
24a-F0	GGCTACGGTCTCGTTGATAAGGATCCGAATTCGAGC
24a-R0	GGCTACGGTCTCTTCTCCTTCTTAAAGTTAAACAAAATTATTTC
V_1-F	GGCTACGGTCTCTGAGATATACATATGGTCCCTGGAGTAGGCGTTCC A
V_1-R	GGCTACGGTCTCCAACGCCTACTCCAGGGACACCTACTCCTGGGA CACCTACACC
V_2-F	GGCTACGGTCTCCCGTTCCAGGCGTCGGGGT
V_2-R	GGCTACGGTCTCATACTCCAGGGACACCTACTCCTGGGACACCTAC ACCAG
V_3-F	GGCTACGGTCTCAAGTAGGCGTTCCAGGCGTCG
V_3-R	GGCTACGGTCTCGTCAACCTACTCCTGGGACACCTACACCAGG
24a-CF	GGCTACGGTCTCTATGATAAGGATCCGAATTCGAG
24a-CR	GGCTACGGTCTCAAACGCCTACTCCAGGGACCATATGTATATCTCCT TCTTAAAGTTAA
V_1-CF	GGCTACGGTCTCACGTTCCAGGCGTCGGGGT
V_1-CR	GGCTACGGTCTCCTCCAGGGACACCTACTCCTGGGACACCTACACC AGG
V_2-CF	GGCTACGGTCTCCTGGAGTAGGCGTTCCAGGCGTC
V_2-CR	GGCTACGGTCTCTACTCCAGGGACACCTACTCCTGGGACACCTACA CCAGG
V_3-CF	GGCTACGGTCTCTGAGTAGGCGTTCCAGGCGTCGG
V_3-CR	GGCTACGGTCTCCCTCCACCTACTCCTGGGACACCTACACCAGG

Lst-CF	GGCTACGGTCTCCGGAGGTGGCGGAAGCGCG
Lst-CR	GGCTACGGTCTCTTCATTATTTAATGGTGCCCCACAGCACGCCCA
24a-NF	GGCTACGGTCTCTCGAATTCGAGCTCCGTCG
24a-NR	GGCTACGGTCTCGCCGCCATATGTATATCTCCTTCTTAAAGTTAAACAAAATTATTTCT
Lst-NF	GGCTACGGTCTCGGCGGCGACCCATGAACATAGCG
Lst-NR	GGCTACGGTCTCAAATGGTGCCCCACAGCACGC
V_1-NF	GGCTACGGTCTCACATTAAAGTCCCTGGAGTAGGCGTTCCAGG
V_1-NR	GGCTACGGTCTCAGACACCTACTCCTGGGACACCTACACCAGG
V_2-NF	GGCTACGGTCTCATGTCCCTGGAGTAGGCGTTCCAGGCG
V_2-NR	GGCTACGGTCTCCTCCTGGGACACCTACACCAGGCA
V_3-NF	GGCTACGGTCTCCAGGAGTAGGTGTCCCTGGAGTAGGCGTTCCAGG
V_3-NR	GGCTACGGTCTCTTTCGGATCCTTATCAACCTACTCCTGGGACACCTACACCAGG

Supplementary Table 4. The plasmids used in this study.

Plasmid name	Description
pET24a-Lst (Kan^+)	Expression plasmid for the protein Lst
pET24a-ELP(V)$_{90}$ (Kan^+)	Expression plasmid for the protein ELP(V)$_{90}$
pET24a-ELP(V)$_{90}$-Lst (Kan^+)	Expression plasmid for the protein ELP(V)$_{90}$-Lst
pET24a-Lst-ELP(V)$_{90}$ (Kan^+)	Expression plasmid for the protein Lst-ELP(V)$_{90}$
