# Supplementary material for: Eradication of MRSA biofilm-associated implant infections by low-immunogenic sustained-release lysostaphin fused to thermosensitive polypeptides
Source: Nat Commun. 2026 Aug 14;17:10108. doi: 10.1038/s41467-026-76664-4 (PMC13598132; doi:10.1038/s41467-026-76664-4)
Supplement: Supplementary file 2 [file 41467_2026_76664_MOESM2_ESM.pdf]

nature portfolio

Corresponding author(s): Weiping Gao

Last updated by author(s): Jul 10, 2026

Reporting Summary

Nature Portfolio wishes to improve the reproducibility of the work that we publish. This form provides structure for consistency and transparency in reporting. For further information on Nature Portfolio policies, see our Editorial Policies and the Editorial Policy Checklist.

Statistics

For all statistical analyses, confirm that the following items are present in the figure legend, table legend, main text, or Methods section.

n/a	Confirmed	
☐	☒	The exact sample size (*n*) for each experimental group/condition, given as a discrete number and unit of measurement
☐	☒	A statement on whether measurements were taken from distinct samples or whether the same sample was measured repeatedly
☐	☒	The statistical test(s) used AND whether they are one- or two-sided<br>*Only common tests should be described solely by name; describe more complex techniques in the Methods section.*
☒	☐	A description of all covariates tested
☐	☒	A description of any assumptions or corrections, such as tests of normality and adjustment for multiple comparisons
☐	☒	A full description of the statistical parameters including central tendency (e.g. means) or other basic estimates (e.g. regression coefficient) AND variation (e.g. standard deviation) or associated estimates of uncertainty (e.g. confidence intervals)
☒	☐	For null hypothesis testing, the test statistic (e.g. *F*, *t*, *r*) with confidence intervals, effect sizes, degrees of freedom and *P* value noted<br>*Give P values as exact values whenever suitable.*
☒	☐	For Bayesian analysis, information on the choice of priors and Markov chain Monte Carlo settings
☒	☐	For hierarchical and complex designs, identification of the appropriate level for tests and full reporting of outcomes
☒	☐	Estimates of effect sizes (e.g. Cohen's *d*, Pearson's *r*), indicating how they were calculated

Our web collection on statistics for biologists contains articles on many of the points above.

Software and code

Policy information about availability of computer code

Data collection	Excel 2007
Data analysis	GraphPad Prism 5

For manuscripts utilizing custom algorithms or software that are central to the research but not yet described in published literature, software must be made available to editors and reviewers. We strongly encourage code deposition in a community repository (e.g. GitHub). See the Nature Portfolio guidelines for submitting code & software for further information.

Data

Policy information about availability of data

All manuscripts must include a data availability statement. This statement should provide the following information, where applicable:

- Accession codes, unique identifiers, or web links for publicly available datasets
- A description of any restrictions on data availability
- For clinical datasets or third party data, please ensure that the statement adheres to our policy

All data supporting the findings of this study are available within the Article and its supplementary files. The nucleotide sequences of the synthetic constructs generated in this study have been deposited in the NCBI GenBank database under accession numbers PZ629001 (LST gene construct) and PZ629002 (LST_ELP_V90 construct). The RNA sequencing data generated in this study have been deposited in the NCBI Sequence Read Archive (SRA) database under BioProject accession number PRJNA1492776. The dataset includes transcriptomic profiles from PBS, lysostaphin (Lst), and LstELP treatment groups. The protein mass spectrometry molecular weight data generated in this study have been deposited in the PRIDE repository under accession number PXD080789. Any additional requests for

information can be directed to and will be fulfilled by the corresponding authors. Source data are provided with this paper.

Research involving human participants, their data, or biological material

Policy information about studies with human participants or human data. See also policy information about sex, gender (identity/presentation), and sexual orientation and race, ethnicity and racism.

Reporting on sex and gender	Not applicable
Reporting on race, ethnicity, or other socially relevant groupings	Not applicable
Population characteristics	Not applicable
Recruitment	Not applicable
Ethics oversight	Not applicable

Note that full information on the approval of the study protocol must also be provided in the manuscript.

Field-specific reporting

Please select the one below that is the best fit for your research. If you are not sure, read the appropriate sections before making your selection.

[x] Life sciences [ ] Behavioural & social sciences [ ] Ecological, evolutionary & environmental sciences

For a reference copy of the document with all sections, see nature.com/documents/nr-reporting-summary-flat.pdf

Life sciences study design

All studies must disclose on these points even when the disclosure is negative.

Sample size	Sample sizes were chosen based on previous studies and common practice in the field. No statistical method was used to predetermine sample size. Animal group sizes were sufficient to detect biologically relevant differences between experimental groups.
Data exclusions	No data were excluded from the analyses.
Replication	All experiments were independently repeated at least three times with similar results.
Randomization	Animals were randomly assigned to experimental groups.
Blinding	Blinding was not performed during data collection and analysis because the experiments involved objective quantitative measurements.

Reporting for specific materials, systems and methods

We require information from authors about some types of materials, experimental systems and methods used in many studies. Here, indicate whether each material, system or method listed is relevant to your study. If you are not sure if a list item applies to your research, read the appropriate section before selecting a response.

Materials & experimental systems

n/a	Involved in the study	
[ ]	[x]	Antibodies
[ ]	[x]	Eukaryotic cell lines
[x]	[ ]	Palaeontology and archaeology
[ ]	[x]	Animals and other organisms
[x]	[ ]	Clinical data
[x]	[ ]	Dual use research of concern
[x]	[ ]	Plants

Methods

n/a	Involved in the study	
[x]	[ ]	ChIP-seq
[ ]	[x]	Flow cytometry
[x]	[ ]	MRI-based neuroimaging

Antibodies

Antibodies used	FITC anti-mouse CD86 Antibody/APC anti-mouse CD206 Antibody/anti-mouse IgG/IgM Antibody
Validation	Specificity validated by manufacturer's flow cytometry data on official website (Abclonal Biotechnology, Cat.No:A21198/A6785) and the published literatures (DOI:10.1038./s41467-025-56648-6;DOI:10.1002/advs.202506337)

Eukaryotic cell lines

Policy information about cell lines and Sex and Gender in Research

Cell line source(s)	mouse
Authentication	None of the cell lines used were authenticated.
Mycoplasma contamination	Confirm that all cell lines tested negative for mycoplasma contamination.
Commonly misidentified lines (See ICLAC register)	No commonly misidentified cell lines were used.

Animals and other research organisms

Policy information about studies involving animals; ARRIVE guidelines recommended for reporting animal research, and Sex and Gender in Research

Laboratory animals	Kunming male mice (6 weeks old).
Wild animals	The study did not involve wild animals.
Reporting on sex	Only male mice were used in this study to minimize variability associated with hormonal cycles and to ensure stable Staphylococcus aureus infection phenotypes.
Field-collected samples	This study did not involve field-collected samples.
Ethics oversight	All experimental protocols involving animals were approved by the Animal Ethics Committee of Peking University First Hospital.

Note that full information on the approval of the study protocol must also be provided in the manuscript.

Plants

Seed stocks	Not applicable
Novel plant genotypes	Not applicable
Authentication	Not applicable

Flow Cytometry

Plots

Confirm that:

- [x] The axis labels state the marker and fluorochrome used (e.g. CD4-FITC).
- [x] The axis scales are clearly visible. Include numbers along axes only for bottom left plot of group (a 'group' is an analysis of identical markers).
- [ ] All plots are contour plots with outliers or pseudocolor plots.
- [ ] A numerical value for number of cells or percentage (with statistics) is provided.

Methodology

Sample preparation	For flow cytometry analysis, RAW264.7 cells were seeded into 12-well plates at the same density (5×10^4 cells/mL) and allowed to adhere for approximately 8 h in the 37 °C, 5% CO_2 incubator. Cells were treated separately with 1 µM Cy5-labeled Lst or Lst-ELP(V)$_{90}$ for 3 h under standard culture conditions. Subsequently, cells were gently rinsed twice with PBS, detached using a cell scraper, and centrifuged at 600 × g (4 °C, 10 min) to collect cell pellets. The pellets were fixed with 2% paraformaldehyde (PFA) in PBS at room temperature for 15 min to preserve cell integrity, rinsed twice more with PBS, and finally gently resuspended in flow cytometry staining buffer.
Instrument	Beckman Coulter CytoFlex S flow cytometer
Software	FlowJo v10.8 software

Cell population abundance	The percentage of CY5-positive cells and mean fluorescence intensity (MFI) were quantified from the total live-cell population.
Gating strategy	Cells were first gated based on forward scatter (FSC) and side scatter (SSC) characteristics to exclude debris. Doublets were excluded using FSC-A versus FSC-H gating. Cy5-positive cells were identified based on fluorescence intensity compared with untreated control cells.

[x] Tick this box to confirm that a figure exemplifying the gating strategy is provided in the Supplementary Information.
