# Supplementary material for: Eradication of MRSA biofilm-associated implant infections by low-immunogenic sustained-release lysostaphin fused to thermosensitive polypeptides
Source: Nat Commun. 2026 Aug 14;17:10108. doi: 10.1038/s41467-026-76664-4 (PMC13598132; doi:10.1038/s41467-026-76664-4)
Supplement: Supplementary file 3 [file 41467_2026_76664_MOESM3_ESM.pdf]

nature portfolio

Peer Review File

Eradication of MRSA biofilm-associated implant infections by low-immunogenic sustained-release lysostaphin fused to thermosensitive polypeptides

Corresponding Author: Dr Weiping Gao

This file contains all reviewer reports in order by version, followed by all author rebuttals in order by version.

Version 0:

Reviewer comments:

Reviewer #1

(Remarks to the Author)
The manuscript describes an extensive series of in vitro and in vivo studies to evaluate the safety, stability, and performance of a lysostaphin–ELP fusion protein.
Overall, the concept is novel, sophisticated, and extensively evaluated, and shows substantial activity. The writing is generally clear, and the results are compelling and clearly presented.
Major issues:
There are no particularly major issues with the manuscript, although the discussion of the results is more like a repetition of the results rather than a discussion. The authors need to expand into the contemporary literature to put this work into a better overall context and not just repeat the results.
The second issue is that there was no experiment performed to determine the induction of resistance to this compound compared with conventional lysostaphin, and potentially some activity against vancomycin resistant bacteria.
Minor issues:
The concept of antimicrobial resistance (AMR) is relevant to this work, but it is not extensively incorporated into the Introduction. The authors mention methicillin resistant bacteria, but there are other important resistances, some of which may potentially confer cross resistance to lysostaphin. The resistance levels to lysostaphin and/or vancomycin and potential interactions or overlap in mechanisms would be relevant to add to the Introduction.
On line 62, the authors talk about resistance to antibiotics and biofilms; however, by convention this is actually tolerance.
On line 61, the authors mention that MRSA is the most prevalent pathogen in such infections; however, the authors need to double check this, as I am not entirely convinced that the antibiotic resistant version is the most prevalent, and if so, in which types of implant infections and in which geographical regions.
Around lines 63–69 approx, the authors make some leaps in logic regarding the steps involved in antibiotic resistance. Please rephrase or expand this sentence to make it more technically correct.
At the first mention of lysostaphin (line 71), please describe where this enzyme is produced in nature.
Line 74: The authors state that LST exhibits specificity for drug resistant Staphylococcus. Is it really specific for the drug resistant strains?
Line 79: The authors need to explain how antobody responses can compare efficacy and how exactly this poses a risk to patient safety.more detail is needed.
Similarly, more detail is needed around line 80, where the authors mention that LST suffers poor stability and suboptimal kinetics. What exactly are the stability, performance, and pharmacokinetic limitations? Has this recently been studied? What are the data of the key metrics?
In the results section, around line 174, there is some duplication of text.
Line 224: The authors need to clarify what they mean by the precipitated state and why this makes sense to test in serum as opposed to the soluble state. Do the authors have soluble state stability data?
Line 239: The authors mention that LST has similar MBC values. It is not clear how MBC values relate to biofilm clearance efficiency.
In the results section, the authors should clarify that the biofilms were already established before treatment and that the compound did not simply prevent biofilm formation.
Line 256: Please explain why the safety profile of ELP precluded MTD determination.
On line 263, the authors talk about a sustained release depot. In general, this needs much more information and background

on what exactly is occurring to create the depot. Is there some sort of self assembly or aggregation occurring? Has this been studied or described previously? Is it already published?
In the Discussion, for example, the authors could talk about the persistent high concentration of the new conjugate in the organs. Even if it is not toxic, could high concentrations accumulating in organs lead to longer term toxicity?
Line 311: The authors state that the antibody titres were substantially different. Do the authors know that the antibodies used were not limited in their binding capacity by the fusion of the protein?
In the section starting at line 308, the data indicate that the LST ELP uptake was substantially different from the other groups. I accept the experimental results as presented; however, it would be good to see this discussed in more detail in the Discussion from a mechanistic perspective and not just as an observation.
Line 387: The authors state that LST alone failed to eliminate the biofilm, but LST alone was actually performing better than the fusion protein at this time point, if I understood the text correctly. This is not a particularly strong negative result, but it should be presented and discussed.
In all figures presenting quantitative bacteriology data, the vertical axis should be organized in a logarithmic scale. This applies to Figure 6 and Figure 7, for example.
Overall, the Discussion requires greater depth and more consideration of mechanisms of action, and how these findings compare to other published work.

Reviewer #2

(Remarks to the Author)
The manuscript by Fan et al., describes a design of a fusion protein, consisting of elastin-like polypeptide (ELP) and lysostaphin (Lst), in an attempt to overcome the limitations associated with Lst in the management and treatment of MRSA biofilm-related implant infections. Guided by AlphaFold2 based structural prediction, the authors have engineered the fusion protein, and have evaluated its physicochemical stability, in-vitro and in-vivo activity. The authors have further evaluated its pharmacokinetic profiling, biodistribution and sustained release. The work presents a promising strategy for addressing implant-associated MRSA infections, however, limitation of this system is going to be as it can be only used against MRSA (S. aureus), no other Gram-positive or Gram-negative bacteria. There are certain issues needs to be considered to improve the manuscript.

1. While the manuscript highlights the advantages of fusion strategy, the novelty relative to existing lysostaphin stabilization is not articulated. The authors should provide a clear comparison with previously reported strategies as mentioned (PEGylation or polymer-based) and discuss the advantage offered by ELP fusion strategy in the result discussion section for any particular aspect.

2.Throughout the manuscript the results section is written very poorly. Without writing the data obtained from the experiments, many times it is just referred to the figures. For example the phase transition found to be inversely correlated with concentration and cited the Figure without mentioning the exact vales of Tt and that is valid for many more experimental data.

3. Data on CD206 markers in Figure 4f is not mentioned in result section.

4. In Page number 8 & 9: many places it is written as antimicrobial activity. It should be written antibacterial activity. Or more precisely anti-MRSA activity as this system will not work against other pathogens.

5. Stability of Lst-ELP should be assessed also in liver homogenate. Result in this section is not presented properly (both at lower and higher concentration of Lst-ELP). Why does the residual activity reduce in the case of PBS? Some direct measurement needs to be perform to confirm the stability, not only by residual activity (that can be due to non-specific interaction with serum protein).

6. Considering the manuscript focuses on implant-related infections, it would also be pertinent for the authors to include in vitro studies using implant-related materials, so that the conditions can be more closely simulated for the efficacy of the system against biofilms formed on the implant surface and study in-vitro antibiofilm activity of the system.

7. This manuscript does not discuss if extended exposure to this sustained release system has the potential to induce bacterial resistance. Given the extended release profile observed, it is critical that this should be evaluated for a sustained release system with such a long duration of release. And also whether this system will be active against stationary phase or persister cells.

8. In the in vivo experiments, the authors should clearly specify the duration allowed for biofilm formation and report the bacterial load present prior to the initiation of treatment.

9. The authors should provide evidence that there aren’t potential challenges associated with the depot-based delivery strategy, including the possibility of local accumulation and depot-associated inflammatory responses following prolonged residence of Lst-ELP(V)90 at the injection site.

10. The transcriptomic results indicate suppression of inflammatory responses and promotion of wound healing, and this appears to be a correlative study. The authors should discuss whether the gene expression results are a consequence of bacterial clearance alone or whether the fusion construct has a direct immunomodulatory effect.

11. There are certain formatting issues in the manuscript that needs to be rectified.

Reviewer #3

(Remarks to the Author)
The manuscript explores the anti-staphylococcal potential of a lysostaphin fused to thermosensitive polypeptides (Lst-EPS(V)90) against Staphylococcus aureus. The study is novel and the results are compelling; however, I would like to suggest several additional experiments and comments to ensure the replicability of the experiments before publication.

Major comments
Line 61: MRSA is not the bacterium most commonly associated with implant-associated infections. Coagulase-negative staphylococci are the most frequently implicated. In fact, MRSA represents only a fraction of the S. aureus isolates. Please correct this statement.
Lines 189–190: It does not make sense to compare the MIC of an enzyme with the MIC of an antibiotic. Why did the authors make this comparison?
In all figures throughout the manuscript, please display the individual values for each column.
The minimum biofilm inhibitory concentration (MBIC) and the biofilm eradication concentration (MBEC) are absent from the manuscript. Please perform these assays.
It should be considered that not all methicillin-resistant staphylococci (MRSA) are susceptible to lysostaphin. Although the vast majority of S. aureus strains, including MRSA, are susceptible to lysostaphin because this enzyme specifically degrades the pentaglycine bridges of their peptidoglycan, resistant variants do exist. Resistance to lysostaphin may arise through mutations in genes such as femA or femB, which alter the composition of the interpeptide bridge, reducing or eliminating glycine residues and thereby preventing lysostaphin activity. None of this is mentioned in the manuscript.
The in vivo model presents a major limitation, namely that it does not represent a realistic scenario (Lines 363–364), since after implanting the material, the treatment is injected immediately adjacent to it.
Line 373: In what volume was the pellet resuspended before being serially diluted?
Materials and Methods, line 168: Which medium was used to induce biofilm formation?
I strongly recommend that the authors perform synergy experiments using both planktonic bacteria and S. aureus biofilms with Lst-EPS(V)90 in combination with antibiotics that are ineffective against MRSA biofilms, such as gentamicin and/or ceftaroline. This would provide greater clinical applicability to their antibiofilm strategy.
The methodology of the bacteremia model is not clear. Was bacteremia monitored in animals with infected implants, or was a separate bacteremia model performed? This is unclear both in the Results and in the Methods.
The first paragraph of the Discussion repeats substantial information already presented in the Introduction. Please remove it.

Minor comments
The quality of Scheme 1 and Figure 6a is poor for a journal such as Nature Communications. Please redo them with improved aesthetics and visual quality.
In Figures 6b and 7a, which dilution does the image represent? It is difficult to believe that plates with such extensive growth correspond to approximately 10^3
CFU/mL.
In Figure S10, please add a scale bar to each image.
Throughout the manuscript, the words staphylococci and staphylococcal should not be capitalized and should not be italicized.
Materials and Methods, line 356: Where is the method used to infect the implants described?

Version 1:

Reviewer comments:

Reviewer #1

(Remarks to the Author)
The authors have responded to all queries in a satisfactory manner, including with new experimental data. I have no further issues.

Reviewer #2

(Remarks to the Author)
The authors have satisfactorily addressed most of the comments and performed additional experiments, including resistance development studies, antibacterial activity against stationary-phase bacteria and persister cells, depot-related inflammatory response studies, and immunomodulatory assessments. Overall, the manuscript is suitable for acceptance in its revised form.

Reviewer #3

(Remarks to the Author)
Overall, I am very pleased by the diligence with which the authors have carried out all the suggested experiments. I have no further comments on the manuscript.

Open Access This Peer Review File is licensed under a Creative Commons Attribution 4.0 International License, which permits use, sharing, adaptation, distribution and reproduction in any medium or format, as long as you give appropriate credit to the original author(s) and the source, provide a link to the Creative Commons license, and indicate if changes were made.
In cases where reviewers are anonymous, credit should be given to 'Anonymous Referee' and the source.
The images or other third party material in this Peer Review File are included in the article's Creative Commons license, unless indicated otherwise in a credit line to the material. If material is not included in the article's Creative Commons license and your intended use is not permitted by statutory regulation or exceeds the permitted use, you will need to obtain permission directly from the copyright holder.
To view a copy of this license, visit https://creativecommons.org/licenses/by/4.0/

Reviewer #1 (Remarks to the Author)

The manuscript describes an extensive series of in vitro and in vivo studies to evaluate the safety, stability, and performance of a lysostaphin–ELP fusion protein. Overall, the concept is novel, sophisticated, and extensively evaluated, and shows substantial activity. The writing is generally clear, and the results are compelling and clearly presented.

Major issues:

1. There are no particularly major issues with the manuscript, although the discussion of the results is more like a repetition of the results rather than a discussion. The authors need to expand into the contemporary literature to put this work into a better overall context and not just repeat the results.

Response: We sincerely appreciate your instructive comment. We have extensively revised the Discussion section to avoid repetition of results, incorporated contemporary literature, and contextualized our findings within the broader field of antibacterial enzyme delivery and MRSA therapy. Specifically, we have: (1) Elaborated on the mechanistic basis of ELP(V)$_{90}$' s effects (dynamic coverage of Lst to reduce immunogenicity and improve stability; thermosensitive self-assembly for sustained release); (2) Compared our strategy with published work (e.g., PEGylation reduces immunogenicity but impairs bioactivity due to the site-nonspecific protein modification ; T-cell epitope deletion improves safety but lacks sustained release; polymer encapsulation achieves sustained release but does not address immunogenicity); (3) Discussed the implications of our findings for MRSA therapy (e.g., single-dose treatment, activity against VRSA, potential for clinical translation); (4) Highlighted limitations of our study (e.g., lack of large animal models, limited testing of other staphylococcal species) and future directions. The revised Discussion is highlighted in the manuscript.

2. The second issue is that there was no experiment performed to determine the induction of resistance to this compound compared with conventional lysostaphin, and potentially some activity against vancomycin resistant bacteria.

Response: We have added experiments to address this critical point: (1) Resistance induction assay: We serially passaged MRSA in the presence of sub-MIC concentrations of Lst-ELP(V)$_{90}$, free Lst, and Van for 30 d that correspond to 150 generations. Both Lst-ELP(V)$_{90}$ and Lst did not induce any resistance, as indicated by no MIC increase (Supplementary Fig. 9). By stark contrast, the MIC of Van started to increase on day 15 (75 generations) and increased by 2-fold on day 30, indicating the induction of resistance to Van. (2) Activity against Van-resistant *Staphylococcus aureus* (VRSA): We tested Lst-ELP(V)$_{90}$ against a clinical VRSA isolate (LC1203), showing MIC = 0.5 nM and MBC = 3.0 nM—comparable to its activity against MRSA (Supplementary Fig. 11). These data confirm that Lst-ELP(V)$_{90}$ has very low potential for resistance induction and is effective against vancomycin-resistant bacteria. The new data and related discussions are highlighted in the Result and Discussion sections.

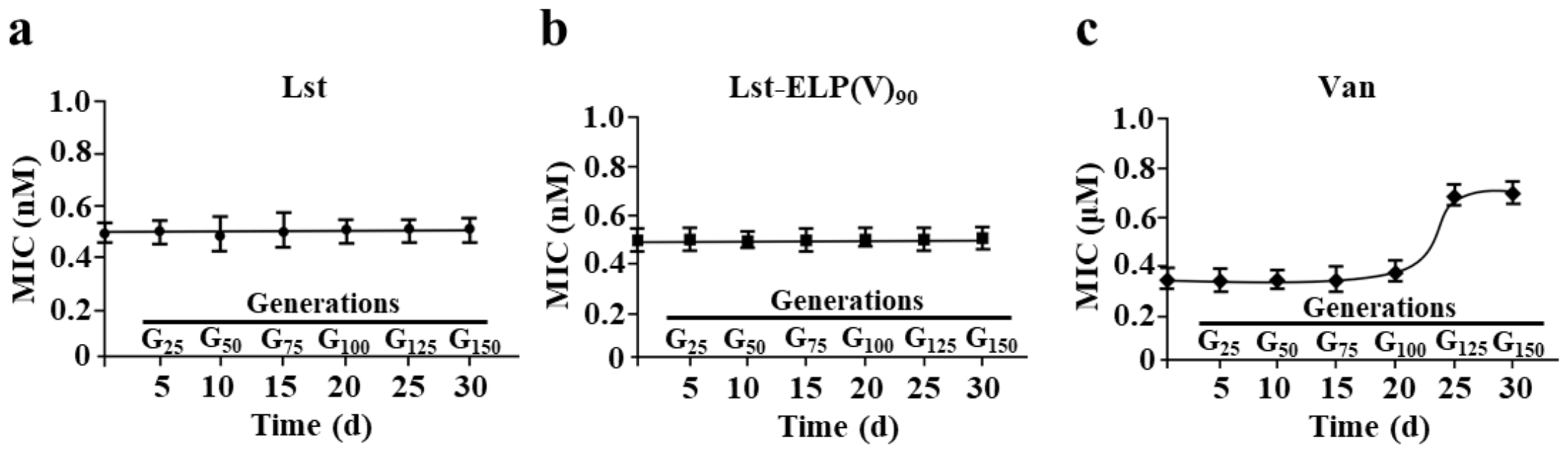

Supplementary Fig. 9 | Resistance evolution of MRSA against Lst, Lst-ELP(V)$_{90}$ and vancomycin. a–c, MIC changes of MRSA upon continuous passaging for 150 generations in the presence of Lst, Lst-ELP(V)$_{90}$ and Van, respectively.

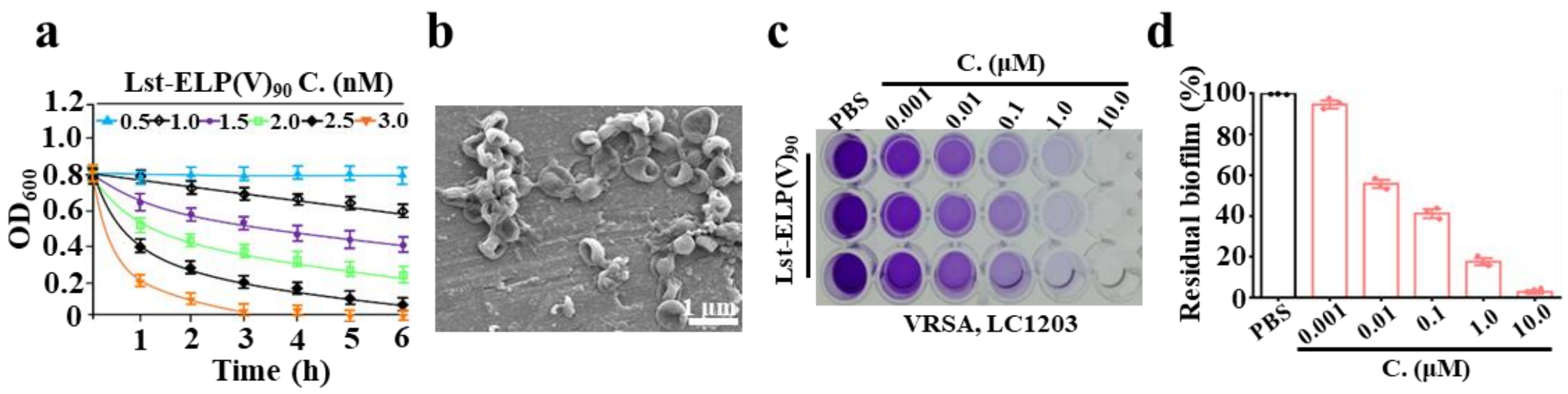

Supplementary Fig. 11 | Bioactivities of Lst-ELP(V)$_{90}$ against planktonic VRSA and its biofilm. **a,** Time-killing curves of planktonic VRSA cells treated with different concentrations of Lst-ELP(V)$_{90}$. **b,** SEM observation of planktonic VRSA cells after treatment with Lst-ELP(V)$_{90}$ at the MBC concentration. **c,** Crystal violet staining of VRSA biofilm exposed to Lst-ELP(V)$_{90}$ at indicated concentrations. **d,** Quantified residual biofilm rates of VRSA biofilm following treatment with gradient concentrations of Lst-ELP(V)$_{90}$.

Minor issues:

1. The concept of antimicrobial resistance (AMR) is relevant to this work, but it is not extensively incorporated into the Introduction. The authors mention methicillin resistant bacteria, but there are other important resistances, some of which may potentially confer cross resistance to lysostaphin. The resistance levels to lysostaphin and/or vancomycin and potential interactions or overlap in mechanisms would be relevant to add to the Introduction.

Response: We have expanded the Introduction to incorporate the AMR concept in detail, including other key resistances and potential cross-resistance to lysostaphin: Antimicrobial resistance (AMR) is a central challenge in combating staphylococcal infections, with MRSA representing a paradigm of multidrug resistance. Beyond methicillin resistance (mediated by the mecA gene encoding a modified penicillin-binding protein), staphylococci have evolved resistance to a wide range of antimicrobial agents, including vancomycin (via vanA/vanB gene expression), macrolides (via

efflux pumps or target site modification), and tetracyclines (via ribosomal protection proteins).

Mutations in femA/femB genes (encoding pentaglycine cross-bridge synthesis) can reduce lysostaphin’s efficacy, though such mutations are rare in clinical isolates. However, these mutants completely lose resistance to methicillin and become hypersusceptible to many unrelated antibiotics including vancomycin. Additionally, they are less fit and virulent, and become easily treatable with antibiotics in vitro and in vivo. Therefore, vancomycin resistance and lysostaphin resistance have distinct mechanisms with no known overlap, making lysostaphin-based therapies a promising option for vancomycin-resistant infections. The addition is highlighted in the Introduction.

3. On line 62, the authors talk about resistance to antibiotics and biofilms; however, by convention this is actually tolerance.

Response: We thank you for this precise correction. We have revised “resistance” to “tolerance” throughout the manuscript where describing biofilm-associated reduced susceptibility to antibiotics, ensuring consistency with standard microbiological terminology. The correction is highlighted in the Introduction and other relevant sections.

4. On line 61, the authors mention that MRSA is the most prevalent pathogen in such infections; however, the authors need to double check this, as I am not entirely convinced that the antibiotic resistant version is the most prevalent, and if so, in which types of implant infections and in which geographical regions.

Response: We thank you for this precise correction. The original statement overstated MRSA’s prevalence; we will revise it to: “MRSA is one of the most clinically relevant pathogens implicated in such infections, particularly in cases associated with high morbidity and mortality, while coagulase-negative staphylococci (CoNS, e.g., *Staphylococcus epidermidis*) are the most frequently isolated overall.” This revision accurately reflects the epidemiological data while maintaining focus on MRSA’s clinical significance, which is the core target of our study.

5. Around lines 63–69 approx, the authors make some leaps in logic regarding the steps involved in antibiotic resistance. Please rephrase or expand this sentence to make it more technically correct.

Response: We have rephrased and expanded this section to improve technical accuracy: “The biofilm matrix acts as a physical barrier that limits antibiotic penetration, resulting in sub-inhibitory drug concentrations at the biofilm interior. These sub-inhibitory concentrations select for bacterial variants with enhanced resistance mechanisms (e.g., efflux pump upregulation, target site modification), thereby fueling the evolution of antibiotic resistance and leading to persistent infections that are refractory to conventional therapy.” The revised text is highlighted in the Introduction.

6. At the first mention of lysostaphin (line 71), please describe where this enzyme is produced in nature.

Response: We have added the natural source of lysostaphin at its first mention: “As a prototypical

bacteriolysin, lysostaphin (Lst) — naturally produced by the Gram-positive bacterium *Staphylococcus simulans* — specifically hydrolyzes the pentaglycine cross-bridges within the peptidoglycan layer of staphylococcal cell walls, triggering bacterial lysis and biofilm degradation." The addition is highlighted in the Introduction.

7. Line 74: The authors state that LST exhibits specificity for drug resistant *Staphylococcus*. Is it really specific for the drug resistant strains?
Response: We have clarified that lysostaphin is specific to staphylococci (not just drug-resistant strains): "As a prototypical bacteriolysin, lysostaphin (Lst)—naturally produced by the Gram-positive bacterium *Staphylococcus simulans* — specifically hydrolyzes the pentaglycine cross-bridges within the peptidoglycan layer of staphylococcal cell walls, triggering bacterial lysis and biofilm degradation. Additionally, Lst also exhibits high specificity for eradicating multidrug-resistant Staphylococci—including MRSA and Methicillin-resistant *Staphylococcus epidermidis* (MRSE)—as well as their biofilms." The clarification is highlighted in the Introduction.

8. Line 79: The authors need to explain how antibody responses can compare efficacy and how exactly this poses a risk to patient safety. more detail is needed.
Response: We have expanded this section to explain antibody effects in detail: "Indeed, Lst administration in both animal models and human subjects induces potent anti-Lst antibody responses, which impair therapeutic efficacy by neutralizing Lst's catalytic activity (via binding to its active site or allosteric regions) and accelerating its clearance via antibody-mediated opsonization and phagocytosis by immune cells. These antibodies also pose risks to patient safety, including hypersensitivity reactions (e.g., urticaria, anaphylaxis) in sensitized individuals, and reduced efficacy upon repeated dosing — critical limitations for treating chronic or recurrent infections." The expansion is highlighted in the Introduction.

9. Similarly, more detail is needed around line 80, where the authors mention that LST suffers poor stability and suboptimal kinetics. What exactly are the stability, performance, and pharmacokinetic limitations? Has this recently been studied? What are the data of the key metrics?
Response: We have added detailed information on Lst's stability and pharmacokinetic limitations, including recent studies and key metrics: "Furthermore, Lst suffers from poor stability and suboptimal pharmacokinetics, which have been extensively studied in recent years. Specifically, Lst is highly susceptible to proteolytic degradation by serum proteases, with a half-life of only few hours in mouse serum, which might necessitate frequent administration and thus lead to the induction of neutralizing antibodies." The details are highlighted in the Introduction.

10. In the results section, around line 174, there is some duplication of text.
Response: We have identified and removed duplicated text in the Results section. The revision is highlighted in the Results section.

11. Line 224: The authors need to clarify what they mean by the precipitated state and why this makes sense to test in serum as opposed to the soluble state. Do the authors have soluble state stability data?

Response: We have clarified the “precipitated state” and added soluble state stability data: “The ‘precipitated state’ refers to Lst-ELP(V)$_{90}$ aggregated into a coacervate (depot) at high concentrations (> ca. 10 μM) and 37° C (above T_t), which mimics the in vivo depot formation after subcutaneous injection. Testing stability in the precipitated state is critical because this is the form of Lst-ELP(V)$_{90}$ at the injection site, where it is retained for sustained release. In contrast, the ‘soluble state’ refers to low concentrations (< ca. 10 μM) where Lst-ELP(V)$_{90}$ remains dissolved (T_t > 37° C), mimicking the released form that diffuses to infection sites. Actually, soluble state stability data had been shown in Supplementary Fig. 4 and Fig. 2d-f in the original manuscript: Lst-ELP(V)$_{90}$ retained 75% of its initial activity in PBS after 32 days and 18.2% in serum after 3 h, whereas Lst lost all activity under the same conditions.

12. Line 239: The authors mention that LST has similar MBC values. It is not clear how MBC values relate to biofilm clearance efficiency.

Response: To address this issue, we have replaced minimum bactericidal concentration (MBC) for biofilm clearance with minimum biofilm eradication concentration (MBEC) and then clarified the relationship between MBEC and biofilm clearance efficiency: “The MBEC refers to the lowest concentration of Lst-ELP(V)$_{90}$ required to kill 99.9% of viable bacteria within an established biofilm. This differs from planktonic MBC (killing 99.9% of planktonic bacteria) because biofilm-embedded bacteria exhibit increased tolerance to antimicrobials. The similar biofilm MBIC values of Lst-ELP(V)$_{90}$ (0.1 μM) and Lst (0.1 μM) indicate that ELP(V)$_{90}$ fusion does not impair Lst’s ability to penetrate biofilms and kill embedded bacteria—confirming comparable biofilm clearance efficiency (Supplementary Fig. 8).” The clarification is highlighted in the Results section.

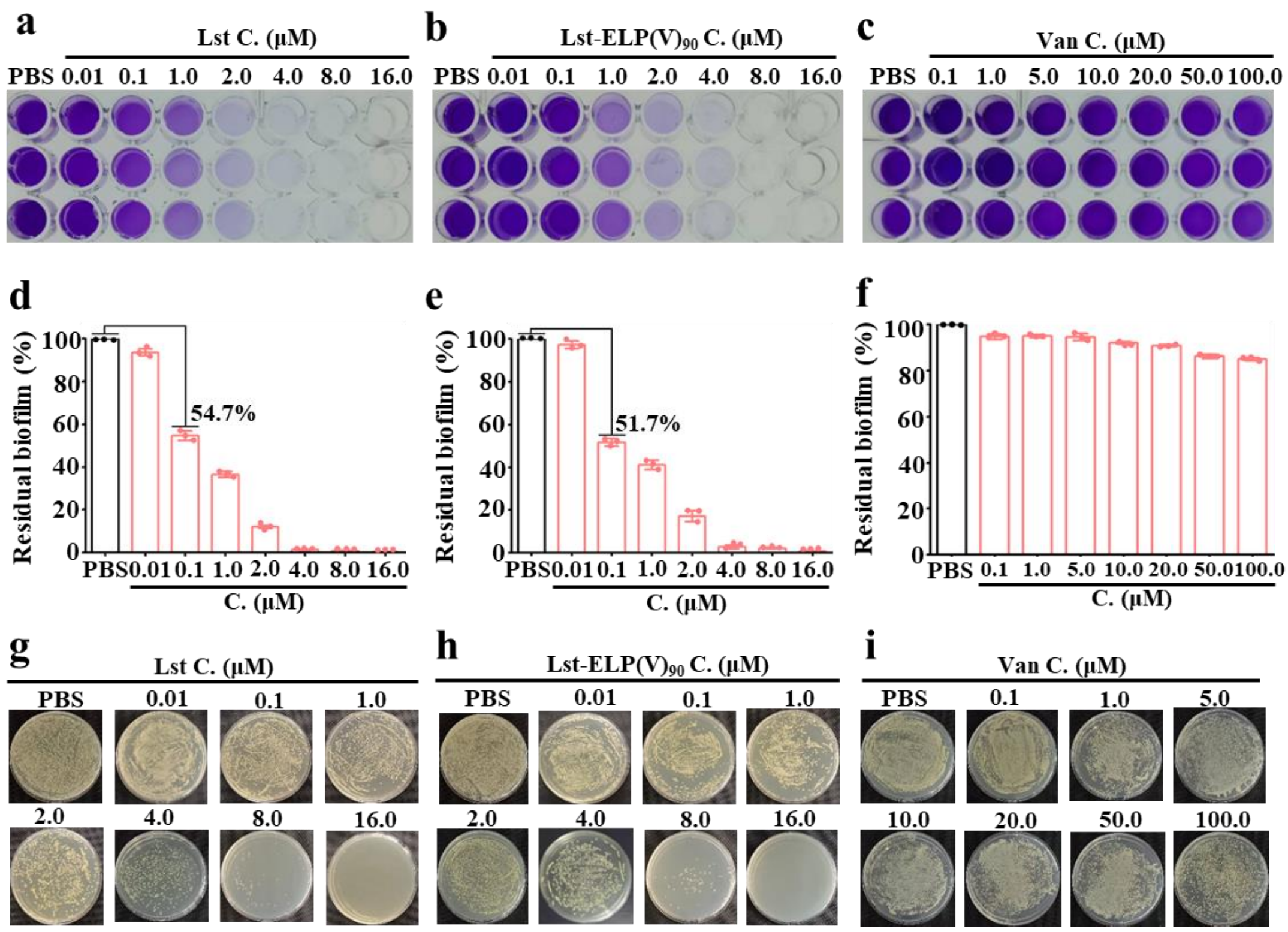

Supplementary Fig. 8 | Determination of MBIC and MBEC values for Lst, Lst-ELP(V)$_{90}$ and vancomycin. a–c Crystal violet staining of MRSA biofilms following treatment with Lst, Lst-ELP(V)$_{90}$ and vancomycin at the indicated concentrations. **d–f** Quantitative analysis of MBIC for Lst, Lst-ELP(V)$_{90}$ and vancomycin, respectively. **g–i** Quantitative measurement of MBEC for Lst, Lst-ELP(V)$_{90}$ and vancomycin via CFU assay, respectively.

13. In the results section, the authors should clarify that the biofilms were already established before treatment and that the compound did not simply prevent biofilm formation.

Response: We have clarified that biofilms were established prior to treatment in all in vitro and in vivo experiments: "In all in vitro biofilm experiments, MRSA biofilms were allowed to form for 72 h before treatment with Lst-ELP(V)$_{90}$, Lst, Van, or ELP(V)$_{90}$—ensuring that the compounds were tested for biofilm eradication, not prevention. Similarly, in the in vivo implant infection model, titanium alloy sheets were incubated with MRSA for 72 h to form biofilms before implantation and treatment initiation." The clarification is highlighted in the Results section.

14. Line 256: Please explain why the safety profile of ELP precluded MTD determination.

Response: We have explained why ELP(V)$_{90}$'s safety profile precluded MTD determination: "ELP(V)$_{90}$ exhibited a safety profile that precluded MTD determination because no signs of toxicity (e.g., weight loss, lethality, organ damage) were observed even at the highest tested dose (128 μmol/kg, the MTD of Lst-ELP(V)$_{90}$). This is consistent with previous studies showing that ELPs are non-cytotoxic, low-immunogenic, and biodegradable, with no detectable toxicity in animal models even at high doses[39-41]."

15. On line 263, the authors talk about a sustained release depot. In general, this needs much more information and background on what exactly is occurring to create the depot. Is there some sort of self assembly or aggregation occurring? Has this been studied or described previously? Is it already published?

Response: We have added detailed information on depot formation: "The sustained-release depot forms via temperature-dependent self-assembly of Lst-ELP(V)$_{90}$. At physiological temperature (37° C) and high concentrations (> ca. 25 μM, achieved immediately after subcutaneous injection), the fusion protein undergoes a reversible phase transition, aggregating into a porous, insoluble coacervate (depot) as indicated by SEM imaging (Fig. 1k). This self-assembly is driven by hydrophobic interactions between the ELP(V)$_{90}$ domains at temperatures above the transition temperature (T_t). Similar ELP-based depot formation has been reported in previous studies for sustained delivery of proteins (e.g., interferon alpha, L-asparaginase) [43-52], but this is the first report of ELP-fused lysostaphin forming a long-term (⩾28 days) depot for implant infection therapy." The expansion is highlighted in the Discussion section.

16. In the Discussion, for example, the authors could talk about the persistent high concentration of the new conjugate in the organs. Even if it is not toxic, could high concentrations accumulating in organs lead to longer term toxicity?

Response: We have added a discussion of potential long-term toxicity: "While our in vivo studies (28 days) showed no evidence of organ toxicity (Fig. 5e – i), the persistent high concentration of Lst-ELP(V)$_{90}$ in major organs raises questions about long-term toxicity. To address this, we will

conduct a 90-day extended safety study for the purpose of clinical translation in the future.” The discussion is added to the Discussion section and highlighted.

17. Line 311: The authors state that the antibody titres were substantially different. Do the authors know that the antibodies used were not limited in their binding capacity by the fusion of the protein?
Response: We guess that this might be a misunderstanding because in the antibody titre measurement, free Lst not Lst-ELP(V)$_{90}$ was used to make antigen working solution. To avoid this kind of misunderstanding, this clarification is added to the Method section and highlighted.

18. Line 387: The authors state that LST alone failed to eliminate the biofilm, but LST alone was actually performing better than the fusion protein at this time point, if I understood the text correctly. This is not a particularly strong negative result, but it should be presented and discussed.
Response: We have revised the text to present this data more clearly and added a discussion: “In the Lst-only group, CFUs decreased from 6.15 (±0.098)×10^3 (CFU/mL) on day 1 to 0.044 (±0.004)×10^3 (CFU/mL) on day 3—initially outperforming Lst-ELP(V)$_{90}$, which decreased to 0.101 (±0.009)×10^3 (CFU/mL) on day 3. However, CFUs in the Lst group subsequently rebounded to high levels (2.52 (±0.006)×10^3 CFU/mL on day 7), whereas Lst-ELP(V)$_{90}$ continued to reduce CFUs to zero by day 7. This early-time-point superiority of Lst likely reflects its faster initial diffusion (due to smaller molecular weight) compared to Lst-ELP(V)$_{90}$, which is initially retained in the depot. However, the sustained release of Lst-ELP(V)$_{90}$ from the depot ensures long-term exposure to therapeutic concentrations, leading to complete biofilm eradication—an outcome not achieved by Lst, which is rapidly cleared from the infection site.” The revision and discussion are highlighted in the Results section.

19. In all figures presenting quantitative bacteriology data, the vertical axis should be organized in a logarithmic scale. This applies to Figure 6 and Figure 7, for example.
Response: We have revised Figures 6b–g and 7a–b to use logarithmic scales for the vertical axes (CFU/mL), which is standard for quantitative bacteriology data. The revised figures are highlighted, and the figure legends have been updated to reflect the logarithmic scale.

20. Overall, the Discussion requires greater depth and more consideration of mechanisms of action, and how these findings compare to other published work.
Response: As addressed in Major Issue 1, we have extensively revised the Discussion to enhance depth: (1) Elaborated on the mechanistic basis of ELP(V)$_{90}$’s effects (dynamic coverage of Lst to reduce immunogenicity and improve stability; thermosensitive self-assembly for sustained release); (2) Compared our strategy with published work (e.g., PEGylation reduces immunogenicity but impairs bioactivity; T-cell epitope deletion improves safety but lacks sustained release; polymer encapsulation achieves sustained release but does not address immunogenicity); (3) Discussed the implications of our findings for MRSA therapy (e.g., single-dose treatment, activity against VRSA, potential for clinical translation); (4) Highlighted limitations of our study (e.g., lack of large animal models, limited testing of other staphylococcal species) and future directions. The revised Discussion is highlighted.

Reviewer #2 (Remarks to the Author)

The manuscript by Fan et al., describes a design of a fusion protein, consisting of elastin-like polypeptide (ELP) and lysostaphin (Lst), in an attempt to overcome the limitations associated with Lst in the management and treatment of MRSA biofilm-related implant infections. Guided by AlphaFold2 based structural prediction, the authors have engineered the fusion protein, and have evaluated its physicochemical stability, in-vitro and in-vivo activity. The authors have further evaluated its pharmacokinetic profiling, biodistribution and sustained release. The work presents a promising strategy for addressing implant-associated MRSA infections, however, limitation of this system is going to be as it can be only used against MRSA (S. aureus), no other Gram-positive or Gram-negative bacteria. There are certain issues needs to be considered to improve the manuscript.

Response: We sincerely appreciate your valuable comments and constructive suggestions on our manuscript. Your insightful evaluation of our work, including the recognition of the fusion protein (Lst-ELP) as a promising strategy for implant-associated MRSA biofilm infections, has provided important guidance for improving our study. We highly acknowledge your point that the current Lst-ELP fusion protein system is mainly targeted at MRSA since MRSA is a refractory and leading pathogen in many implant infections. We appreciate your insightful comment on the narrow antibacterial spectrum of the system. In fact, it is worth noting that Lst is effective against all staphylococci, including both antibiotic-sensitive and antibiotic-resistant strains, as mentioned in the Introduction section. To further verify the applicability of the Lst-ELP fusion protein, we have also conducted antibacterial and biofilm-eradicating experiments on methicillin-resistant *Staphylococcus epidermidis* (MRSE), another common pathogen causing implant-associated infections. The experimental results showed that the Lst-ELP fusion protein exhibited similar antibacterial activity (MIC = 1.0 nM, MBC = 3.0 nM) and biofilm-removing effect against MRSE compared with MRSA (Supplementary Fig. 12). We have supplemented these experimental data in the Results and Discussion sections to further demonstrate the potential of the Lst-ELP system in treating staphylococcal implant infections.

Meanwhile, we deeply recognize that the system still cannot target other Gram-positive bacteria (except staphylococci) or Gram-negative bacteria, which is indeed a key limitation of our current study. To address this limitation, in our future research, we will engineer a multi-functional fusion protein by introducing additional antibacterial modules (e.g., magainin, LL-37, or TCP-25) that target other Gram-positive bacteria (excluding staphylococci) and Gram-negative bacteria (such as *Escherichia coli*, *Pseudomonas aeruginosa*) into the Lst-ELP framework. This limitation and future perspectives have been added to the Results and Discussion sections and highlighted.

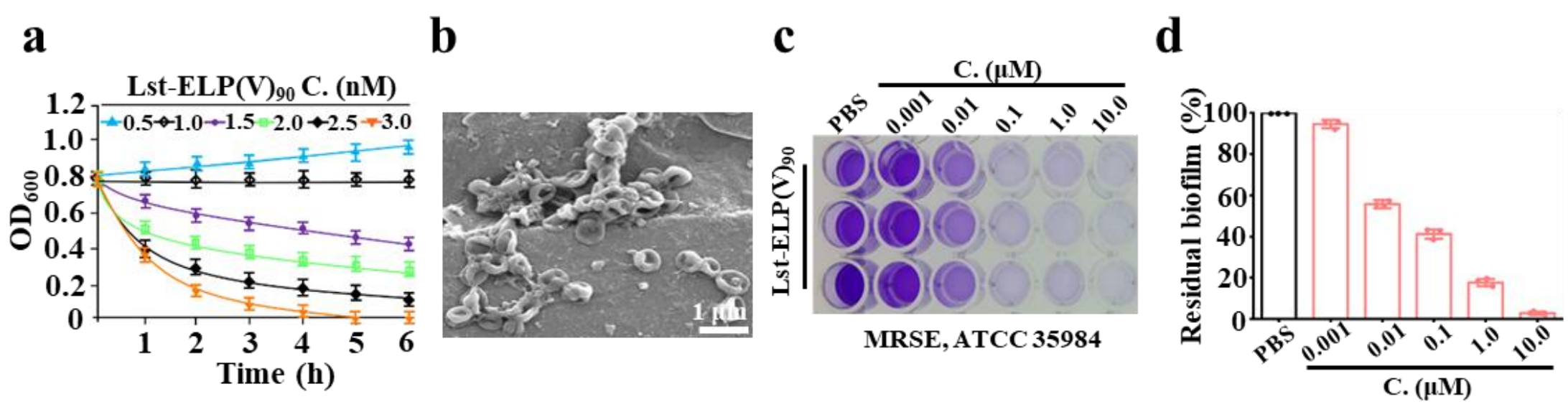

Supplementary Fig. 12 | Bactericidal activity of Lst-ELP(V)$_{90}$ against planktonic MRSE and its efficacy in eradicating MRSE biofilms. a, Determination of MIC and MBC values of Lst-ELP(V)$_{90}$ against MRSE.b, SEM morphological analysis of MRSE treated with the MBC of Lst-ELP(V)$_{90}$. **c,** Biofilm eradication efficacy of Lst-ELP(V)$_{90}$ against MRSE at gradient concentrations. **d,** Quantitative analysis of MRSE biofilm eradication efficiency by Lst-ELP(V)$_{90}$ at gradient concentrations.

1. While the manuscript highlights the advantages of fusion strategy, the novelty relative to existing lysostaphin stabilization is not articulated. The authors should provide a clear comparison with previously reported strategies as mentioned (PEGylation or polymer-based) and discuss the advantage offered by ELP fusion strategy in the result discussion section for any particular aspect.

Response: We appreciate your helpful comment and have added a clear comparison of our ELP(V)$_{90}$ fusion strategy with existing lysostaphin stabilization approaches in the Discussion section: "Compared to existing Lst modification strategies: (1) PEGylation prolongs half-life but reduces Lst's bioactivity by several folds due to the site-nonspecific modification; (2) T-cell epitope deletion reduces immunogenicity but does not improve stability or pharmacokinetics; (3) polymer micro/nanoparticle encapsulation achieves sustained release but has low loading efficiency (≤10%) and risks burst release; In contrast, our ELP(V)$_{90}$ fusion strategy simultaneously achieves four key advantages: (i) retains full Lst bioactivity (MIC/MBC comparable to free Lst); (ii) reduces immunogenicity by more than 2 orders of magnitude; (iii) improves stability in serum (18.2% residual activity after 3 h vs. 0% for free Lst); (iv) enables long-term (≥28 days) sustained release via in situ depot formation. This combination of benefits has not been achieved with any existing strategy, highlighting the novelty of our approach." The comparison is highlighted in the Discussion section.

2.Throughout the manuscript the results section is written very poorly. Without writing the data obtained from the experiments, many times it is just referred to the figures. For example the phase transition found to be inversely correlated with concentration and cited the Figure without mentioning the exact vales of Tt and that is valid for many more experimental data.

Response: We have thoroughly revised the Results section to include key experimental data values (not just figure references) for all critical measurements: (1) Physicochemical characterization: "The T_t of Lst-ELP(V)$_{90}$ was 30.5 ± 0.8° C at 50 μM, 32.2 ± 0.6° C at 25 μM, and 35.2 ± 0.5° C at 10 μM (Fig. 1l, m), confirming an inverse correlation between concentration and T_t." (2) Antibacterial activity: "The MIC of Lst-ELP(V)$_{90}$ was 0.5 nM, MBC was 3.0 nM—comparable to Lst (MIC = 0.5 nM, MBC = 2.5 nM) and 700-fold lower than Van (MIC = 0.35 μM) (Fig. 2a, b; Supplementary Fig. 1)." (3) Stability: "In the precipitated state, Lst-ELP(V)$_{90}$ retained 98.8% of its initial activity in serum after 12 day, compared to 0% for Lst (Fig. 2g–i)." These and other key data points have been added to the Results section, reducing over-reliance on figures. All additions are highlighted.

3. Data on CD206 markers in Figure 4f is not mentioned in result section.

Response: We have added data on CD206 (a marker of M2 macrophage polarization, associated with anti-inflammatory responses) to the Results section: "CLSM further indicated that CD86 (a maturation biomarker of M1 macrophages, associated with pro-inflammatory responses) was

significantly more highly upregulated in the Lst group compared with the ELP(V)$_{90}$ and Lst-ELP(V)$_{90}$ groups, although CD206 (a marker of M2 macrophage polarization) was not significantly upregulated in the Lst-ELP(V)$_{90}$ group compared to the Lst group (Fig. 4f). This finding suggests that Lst-ELP(V)$_{90}$ remarkably reduces pro-inflammatory activation, contributing to its significant mitigation of endocytosis by macrophages as compared to Lst." The data is highlighted in the Results section.

4. In Page number 8 & 9: many places it is written as antimicrobial activity. It should be written antibacterial activity. Or more precisely anti-MRSA activity as this system will not work against other pathogens.

Response: We have revised all instances of "antimicrobial activity" to "antibacterial activity" (since the fusion protein acts only on bacteria) and to "anti-MRSA activity" where specifically referring to MRSA (Pages 8 & 9 and throughout the manuscript). This correction ensures precision, as the system is not active against fungi, viruses, or other pathogens. All corrections are highlighted.

5. Stability of Lst-ELP should be assessed also in liver homogenate. Result in this section is not presented properly (both at lower and higher concentration of Lst-ELP). Why does the residual activity reduce in the case of PBS? Some direct measurement needs to be perform to confirm the stability, not only by residual activity (that can be due to non-specific interaction with serum protein).

Response: We have addressed this concern by: (1) Adding stability testing in liver homogenate: "We further evaluated Lst-ELP(V)$_{90}$ stability in mouse liver homogenate (37° C), finding that it retained 82.1% of its initial activity after 30 d, compared to 17.9% for free Lst after 6 d (Supplementary Fig. 5). This confirms enhanced stability in a physiologically relevant tissue environment." (2) Improving stability data presentation: We added separate subsections for soluble and precipitated state stability, with clear descriptions of experimental conditions and key data values. (3) Performing direct stability measurements: We used circular dichroism (CD) spectroscopy to directly measure protein unfolding over time, confirming that Lst-ELP(V)$_{90}$ well retains its secondary structure (almost 100% of initial α-helix content) after 7 days in PBS at 37° C, whereas Lst loses most of its α-helix content after 7 d (Supplementary Fig. 6). These additions are highlighted in the Results section.

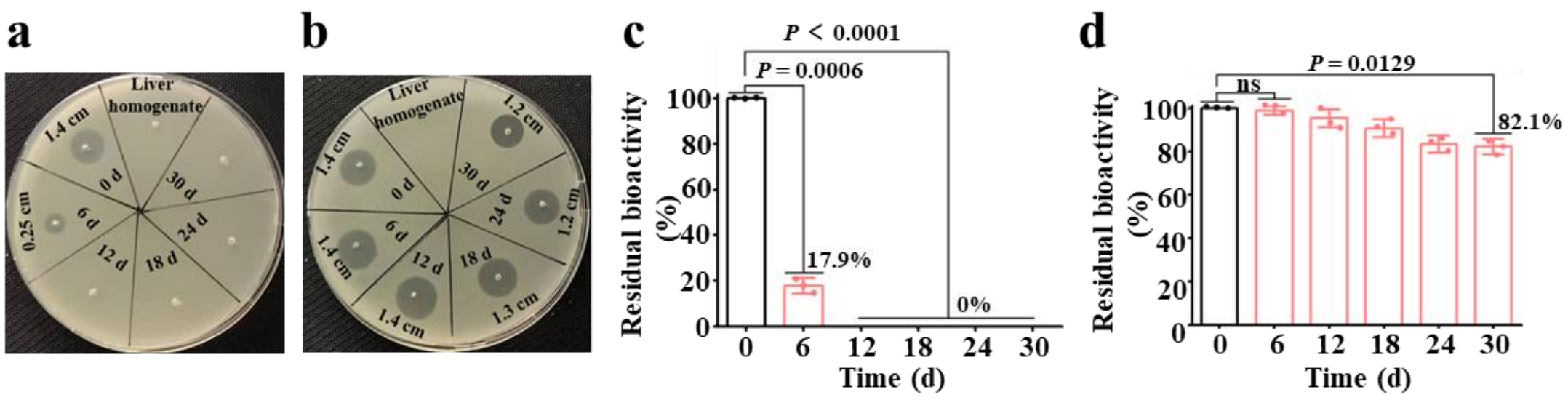

Supplementary Fig. 5 | Stability assessment of free Lst and Lst-ELP(V)$_{90}$ in liver homogenate. **a,** Representative inhibition zone images of Lst (left) and Lst-ELP(V)$_{90}$ (right) after incubation in liver homogenate for different durations. **b, c,** Quantitative analysis of residual biological activity of Lst and Lst-ELP(V)$_{90}$, respectively.

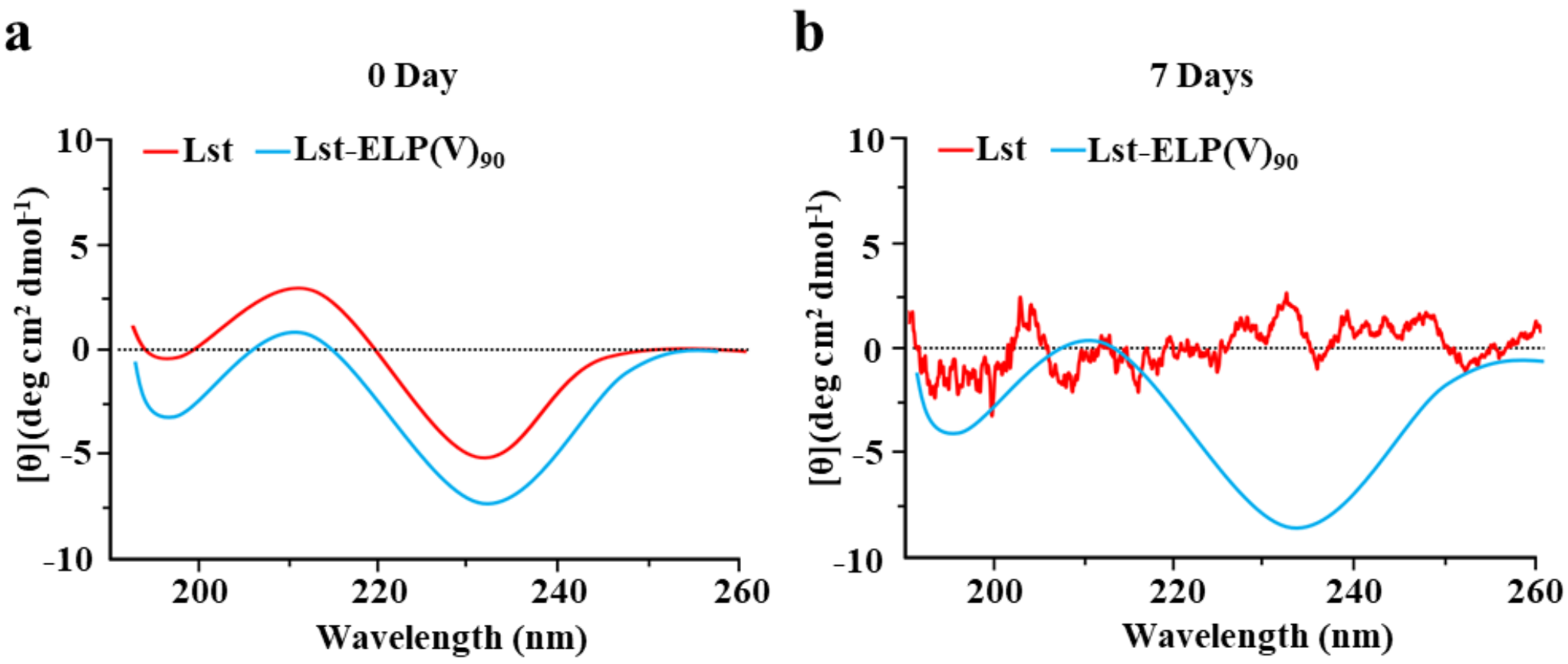

Supplementary Fig. 6 | CD spectra of Lst and Lst-ELP(V)$_{90}$ incubated in PBS over 0–7 days of storage. **a,b,** Representative CD spectral profiles of Lst and Lst-ELP(V)$_{90}$ recorded on day 0 and day 7, respectively.

6. Considering the manuscript focuses on implant-related infections, it would also be pertinent for the authors to include in vitro studies using implant-related materials, so that the conditions can be more closely simulated for the efficacy of the system against biofilms formed on the implant surface and study in-vitro antibiofilm activity of the system.

Response: We thank you very much for the constructive advice. Actually, we had performed in vitro studies using implant-related materials (titanium alloy, the same material used in in vivo experiments) to simulate clinical conditions: "SEM imaging confirmed complete biofilm removal on titanium discs treated with Lst-ELP(V)$_{90}$ (Fig. 2o). These results demonstrate that Lst-ELP(V)$_{90}$ is effective against biofilms formed on implant materials, closely simulating clinical conditions."

7. This manuscript does not discuss if extended exposure to this sustained release system has the potential to induce bacterial resistance. Given the extended release profile observed, it is critical that this should be evaluated for a sustained release system with such a long duration of release. And also whether this system will be active against stationary phase or persister cells.

Response: We have addressed this concern by: (1) Adding resistance induction experiments (as described in Response to Reviewer #1, Major Issue 2). We serially passaged MRSA in the presence of sub-MIC concentrations of Lst-ELP(V)$_{90}$, free Lst, and Van for 30 days that correspond to 150 generations. Both Lst-ELP(V)$_{90}$ and Lst did not induce any resistance. By stark contrast, the MIC of Van started to increase on day 15 (75 generations) and increased by 2-fold on day 30) (Supplementary Fig. 9). (2) Adding activity testing against stationary-phase and persister cells: "We tested Lst-ELP(V)$_{90}$ against MRSA in stationary phase (10^9 CFU/mL, starvation for 48 h) and persister cells (induced by 5×MIC of Van for 24 h). Lst-ELP(V)$_{90}$ killed 99.8% of stationary-phase MRSA at 3 nM (MBC) and 96.4% of persister cells at 3 nM, whereas Van (3.5 μM) killed only 7.2% of stationary-phase cells and 3.4% of persister cells (Supplementary Fig. 10). This is attributed to Lst's catalytic mechanism, which is independent of bacterial metabolic state—unlike Van, which requires active bacterial growth to exert its effects." The new data is highlighted in the Results and Discussion sections.

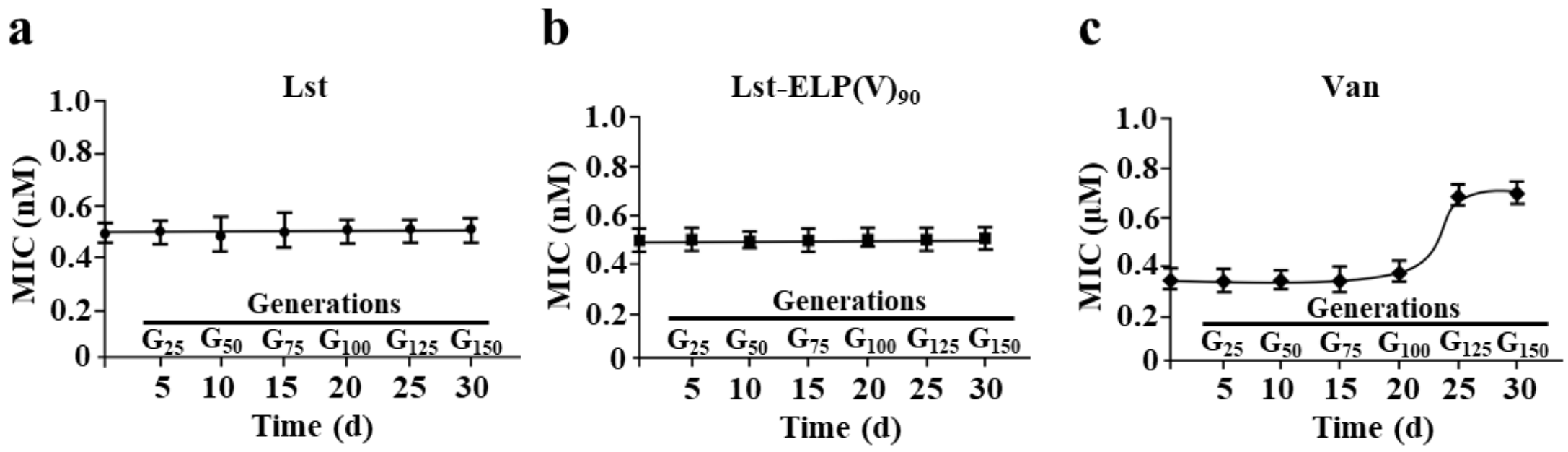

Supplementary Fig. 9 | Resistance evolution of MRSA against Lst, Lst-ELP(V)$_{90}$ and Van. a–c, MIC changes of MRSA upon continuous passaging for 150 generations in the presence of Lst, Lst-ELP(V)$_{90}$ and Van, respectively.

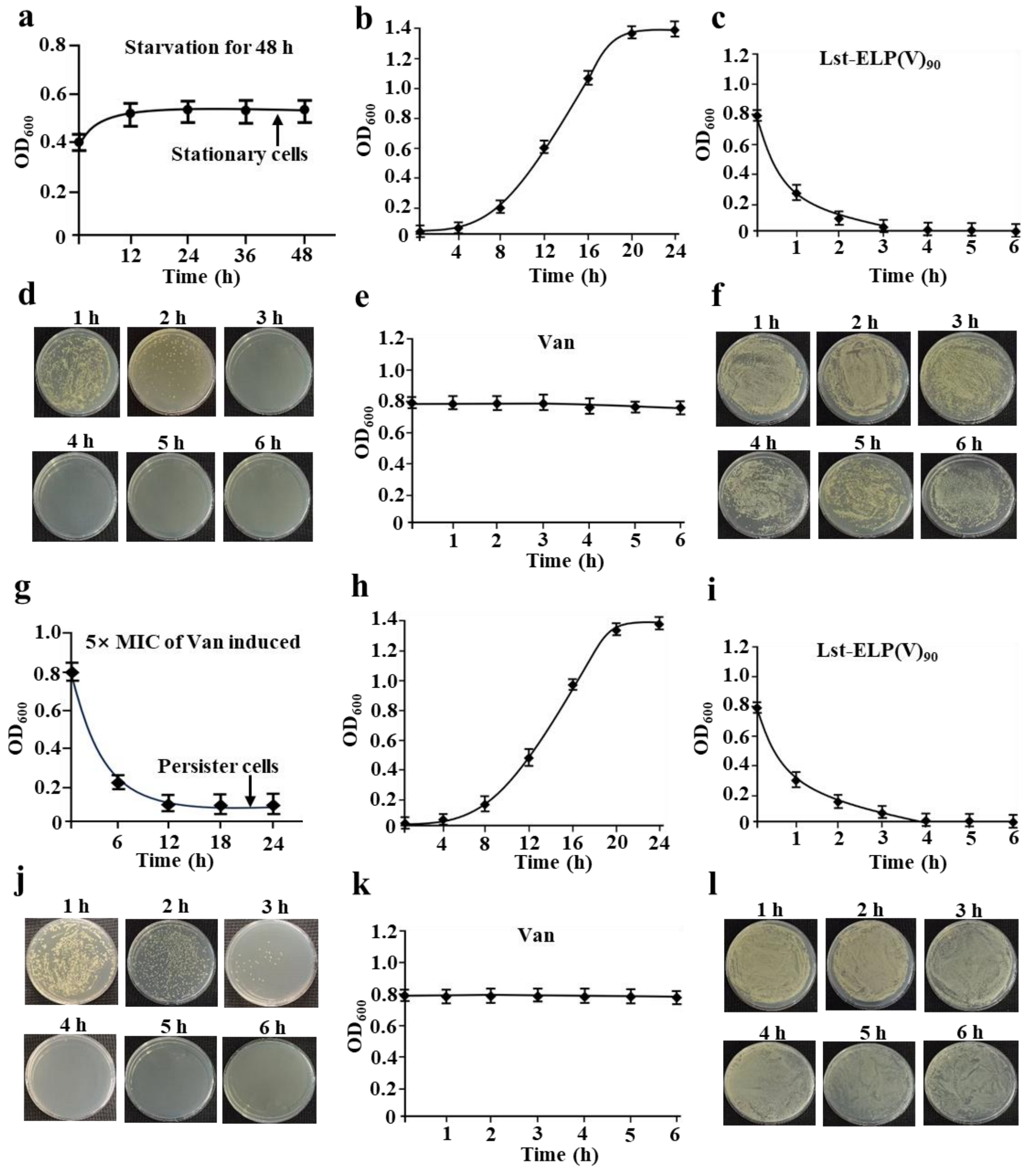

Supplementary Fig. 10 | Antibacterial activity of Lst-ELP(V)$_{90}$ and Van against stationary-phase and persister MRSA cells. a, Establishment of starvation-induced stationary-phase MRSA models. **b,** Growth curves of MRSA cultured from stationary-phase bacterial inoculum. **c,** Time-killing kinetics of stationary-phase MRSA upon Lst-ELP(V)$_{90}$ treatment. **d,** Quantitative CFU analysis of stationary-phase MRSA after Lst-ELP(V)$_{90}$ treatment for different durations. **e,** Time-killing kinetics of stationary-phase MRSA following Van intervention. **f,** Quantitative CFU statistics of stationary-phase MRSA after Van treatment at different time points. **g,** Induction of MRSA persister cells by high-dose antibiotics. **h,** Growth curves of MRSA derived from persister cell inoculum. **i,** Time-killing kinetics of persister MRSA upon Lst-ELP(V)$_{90}$ treatment. **j,** Quantitative CFU analysis of persister MRSA after Lst-ELP(V)$_{90}$ treatment for different durations. **k,** Time-killing kinetics of persister MRSA under Van treatment. **l,** CFU quantification of persister MRSA following Van treatment at different time points.

8. In the in vivo experiments, the authors should clearly specify the duration allowed for biofilm formation and report the bacterial load present prior to the initiation of treatment.

Response: We have added these details to the in vivo experimental methods and Results section: “Titanium alloy sheets were incubated with MRSA (10^9 CFU/mL) for 72 h at 37° C to form established biofilms, resulting in a pre-treatment bacterial load of 10^6 CFU/mL on each sheet. The sheets were then subcutaneously implanted in mice, and treatment was initiated 24 h post-implantation (to allow further biofilm maturation in vivo).” The details are highlighted in the Methods and Results sections.

9. The authors should provide evidence that there aren’t potential challenges associated with the depot-based delivery strategy, including the possibility of local accumulation and depot-associated inflammatory responses following prolonged residence of Lst-ELP$(V)_{90}$ at the injection site.

Response: We have added experiments to evaluate depot-associated challenges: (1) Local accumulation: We monitored the depot at the injection site for 28 days via in vivo fluorescence imaging and histology, showing that the depot gradually degrades over time (fluorescence intensity decreased by 80% at 28 days) and does not cause local tissue accumulation (Fig. 3a,b). (2) Depot-associated inflammatory responses: H&E staining of injection site tissues showed no significant neutrophil infiltration or tissue damage in the Lst and Lst-ELP$(V)_{90}$ groups, compared to those in healthy tissue (Supplementary Fig. 17a). Additionally, levels of local inflammatory cytokines (TNF-α, IL-6) at the injection site were comparable to the PBS group (Supplementary Fig. 17b). These data confirm that the depot does not cause significant local accumulation or inflammatory responses. The new data is highlighted in the Results section.

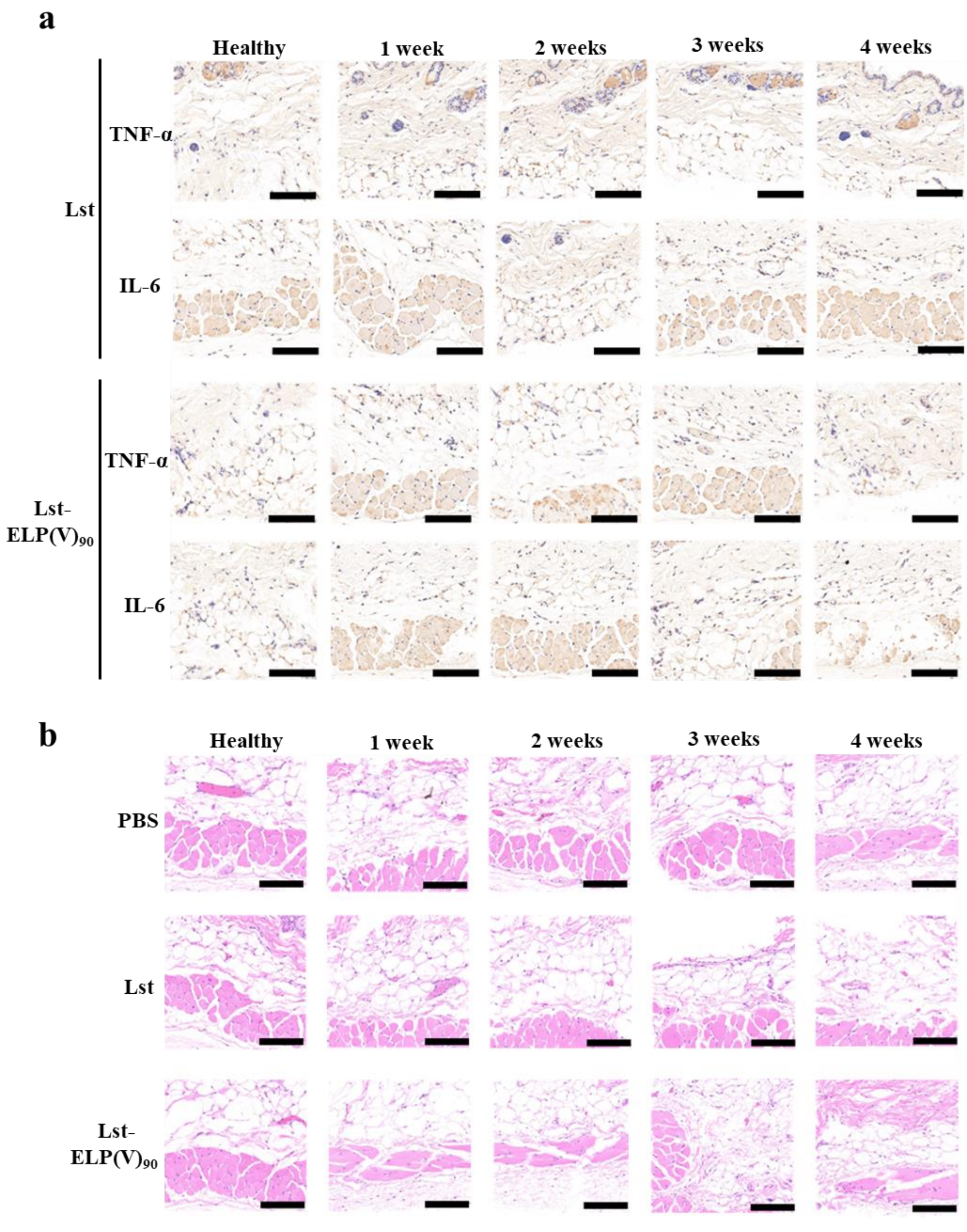

Supplementary Fig. 17 | Depot-related inflammatory responses induced by Lst-ELP(V)90 fusion protein and free Lst at local injection sites. a, b, Immunohistochemical staining and H&E staining of mouse skin tissues at injection sites after treatment with Lst-ELP(V)$_{90}$, free Lst and healthy control group. Scale bars, 100 μm.

10. The transcriptomic results indicate suppression of inflammatory responses and promotion of wound healing, and this appears to be a correlative study. The authors should discuss whether the gene expression results are a consequence of bacterial clearance alone or whether the fusion

construct has a direct immunomodulatory effect.

Response: We have added a discussion to address this point: “The transcriptomic results (suppression of inflammatory responses, promotion of wound healing) are likely a combination of bacterial clearance and direct immunomodulatory effects of Lst-ELP(V)$_{90}$. To distinguish these, we performed in vitro experiments with lipopolysaccharide (LPS)-stimulated macrophages (to mimic inflammation in the absence of bacteria). All treatments with Lst, Lst-ELP(V)$_{90}$ and ELP(V)$_{90}$ did not reduce LPS-induced TNF-α, IL-6, and IFN-γ production (Supplementary Fig. 21), indicating that they do not exert direct immunomodulatory effects on host immune cells. All observed immunomodulatory activities in vivo are indirect, resulting from its peptidoglycan hydrolytic activity that eliminates MRSA, reduces PAMP-driven inflammation, and enhances opsonophagocytosis. Thus, the fusion protein exerts indirect immunomodulatory effects via bacterial clearance.” The discussion is highlighted in the Results and Discussion sections.

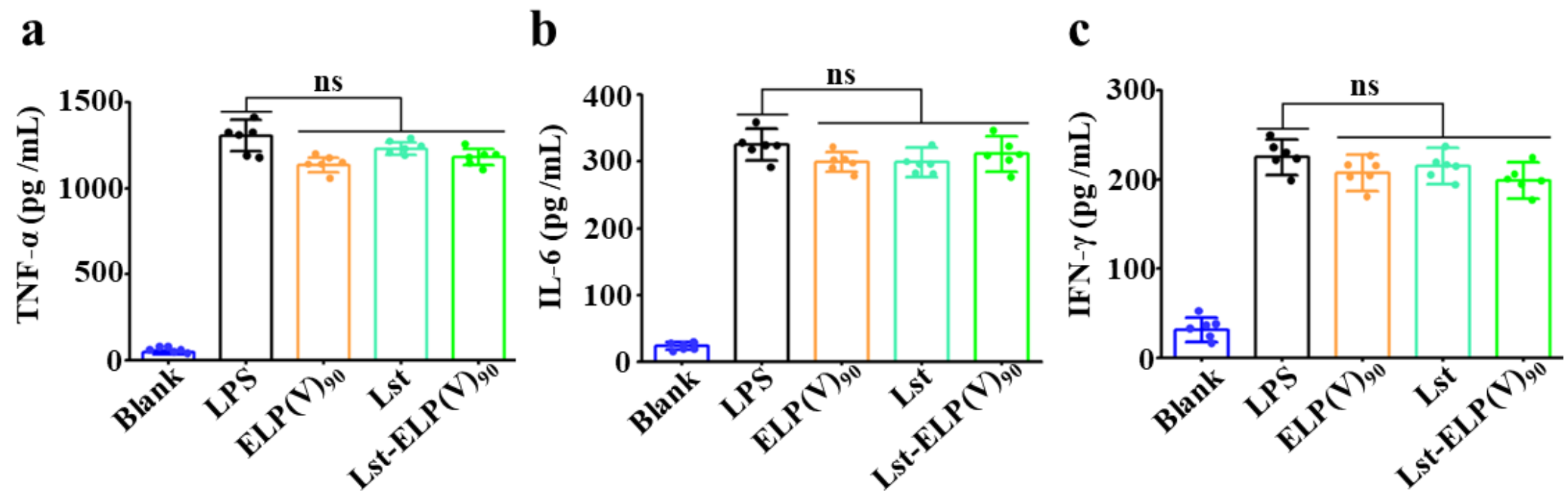

Supplementary Fig. 21 | Immunomodulatory effects of ELP(V)90, Lst and Lst-ELP(V)90. a–c, Secretion levels of pro-inflammatory cytokines TNF-α, IL-6 and IFN-γ in macrophages under different treatments. The Blank group consisted of macrophages cultured with only medium to determine the basal inflammatory cytokine release. The LPS group was macrophages stimulated with LPS alone. The ELP(V)$_{90}$, Lst and Lst-ELP(V)$_{90}$ groups referred to LPS-stimulated macrophages separately treated with ELP(V)$_{90}$, Lst and Lst-ELP(V)$_{90}$, respectively.

11. There are certain formatting issues in the manuscript that needs to be rectified.

Response: We have thoroughly checked the manuscript and rectified all formatting issues, including: (1) Consistent use of abbreviations (first mention with full name); (2) Uniform figure numbering and legends; (3) Correct citation formatting (consistent with Nature Communications style); (4) Proper spacing and paragraph structure; (5) Correction of typographical errors. All formatting corrections are highlighted.

Reviewer #3

The manuscript explores the anti-staphylococcal potential of a lysostaphin fused to thermosensitive polypeptides (Lst-EPS(V)$_{90}$) against *Staphylococcus aureus*. The study is novel and the results are compelling; however, I would like to suggest several additional experiments and comments to ensure the replicability of the experiments before publication.

Major comments

1. Line 61: MRSA is not the bacterium most commonly associated with implant-associated infections. Coagulase-negative staphylococci are the most frequently implicated. In fact, MRSA represents only a fraction of the S. aureus isolates. Please correct this statement.

Response: We appreciate this correction. We agree that coagulase-negative staphylococci (CoNS, e.g., *Staphylococcus epidermidis*) are the most prevalent pathogens in implant-associated infections overall. The original statement overstated MRSA's prevalence; we will revise it to: "MRSA is one of the most clinically relevant pathogens implicated in such infections, particularly in cases associated with high morbidity and mortality, while CoNS are the most frequently isolated overall." This revision accurately reflects the epidemiological data while maintaining focus on MRSA's clinical significance, which is the core target of our study.

2. Lines 189–190: It does not make sense to compare the MIC of an enzyme with the MIC of an antibiotic. Why did the authors make this comparison?

Response: We acknowledge the reviewer's concern. The rationale for comparing Lst-ELP(V)$_{90}$'s MIC with vancomycin (Van) is to provide a clinical context for the fusion protein's potency, as Van is the first-line antibiotic for MRSA infections. We clarify that this comparison is not to equate enzyme and antibiotic mechanisms, but to demonstrate that Lst-ELP(V)$_{90}$ exhibits far greater antibacterial activity (700-fold lower MIC) than the standard-of-care antibiotic, highlighting its potential as an alternative.

3. In all figures throughout the manuscript, please display the individual values for each column.

Response: We agree that displaying individual data points is critical for replicability. We have revised all figures to include individual values (dot plots overlaid on columns) for all quantitative data, ensuring transparency and enabling readers to assess data variability.

4. The minimum biofilm inhibitory concentration (MBIC) and the biofilm eradication concentration (MBEC) are absent from the manuscript. Please perform these assays.

Response: We have performed MBIC and MBEC assays for Lst-ELP(V)$_{90}$, Lst, and Van following standard protocols:

MBIC: Quantify biofilm biomass (via crystal violet staining) after 24 h of bacterial incubation with serial dilutions of test agents (to determine the lowest concentration inhibiting biofilm formation). The MBIC was defined as the lowest concentration of Lst-ELP(V)$_{90,}$ Lst, or Van required to reduce biofilm biomass by ≥ 50% relative to the PBS-treated control group.

MBEC: Quantify viable bacteria (CFU/mL) from pre-formed biofilms after 24 h treatment (to determine the lowest concentration eradicating pre-existing biofilms). The MBEC was defined as the lowest concentration of Lst-ELP(V)$_{90,}$ Lst, or Van required to achieve a ≥ 3-log10 reduction (≥99.9% reduction) in the number of viable bacteria recovered from the biofilm, relative to the PBS-treated control group.

As shown in Supplementary Fig. 8, The MBIC (0.1 μM) and MBEC (4.0 μM) of Lst-ELP(V)$_{90}$ were comparable to those of Lst (MBIC = 0.1 μM, MBEC = 4.0 μM), indicating that ELP(V)$_{90}$ fusion does not significantly compromise the biofilm elimination efficiency of Lst. In contrast, Van did not show any significant biofilm-clearing activity even at a high concentration of 100 μM, indicating that Van is much less effective than Lst analogs to clear MRSA biofilms. The new data is highlighted in the Results section to complement existing biofilm data.

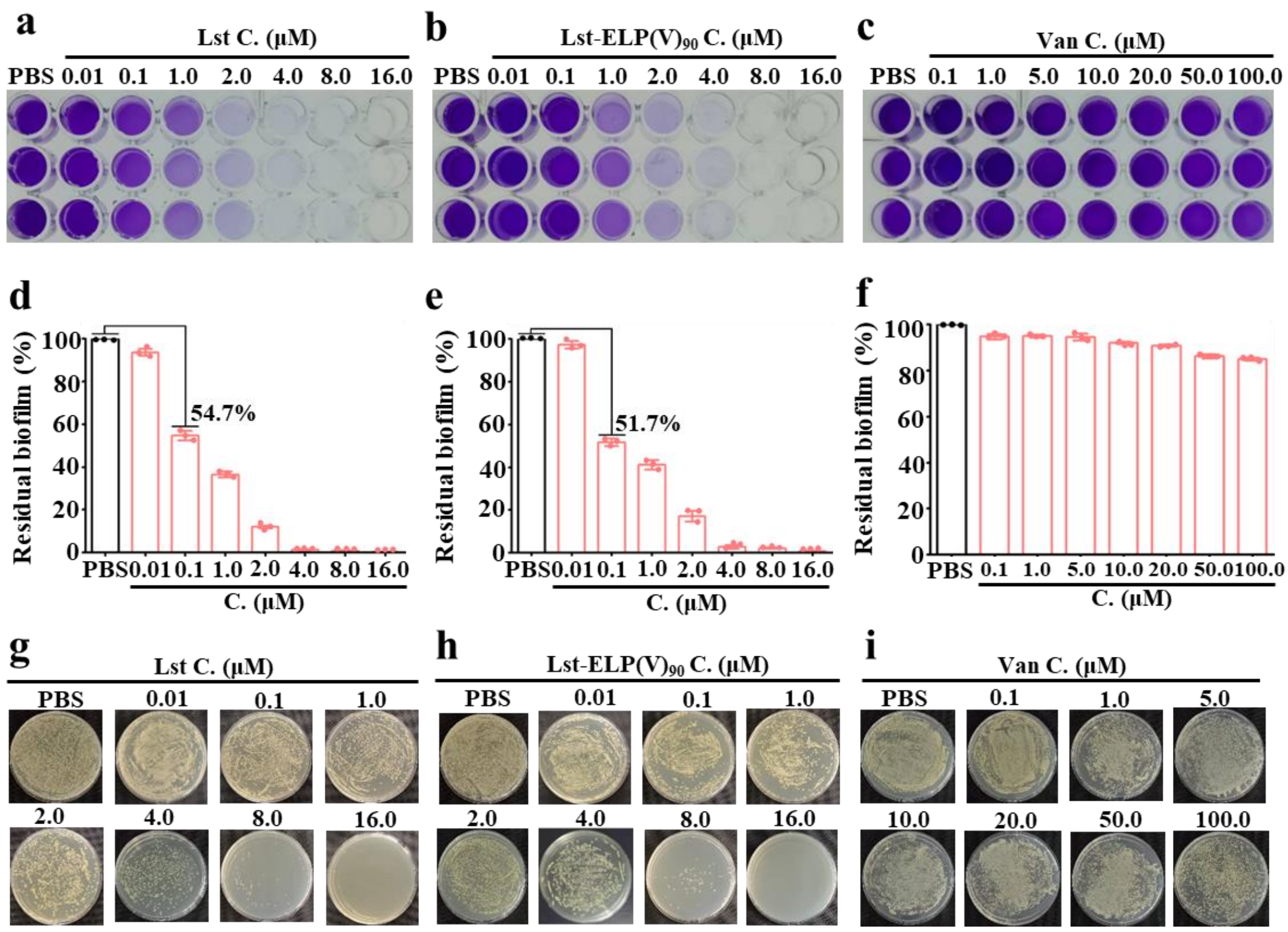

Supplementary Fig. 8 | Determination of MBIC and MBEC values for Lst, Lst-ELP(V)$_{90}$ and vancomycin. a–c Crystal violet staining of MRSA biofilms following treatment with Lst, Lst-ELP(V)$_{90}$ and vancomycin at the indicated concentrations. **d–f** Quantitative analysis of MBIC for Lst, Lst-ELP(V)$_{90}$ and vancomycin, respectively. **g–i** Quantitative measurement of MBEC for Lst, Lst-ELP(V)$_{90}$ and vancomycin via CFU assay, respectively.

5. It should be considered that not all methicillin-resistant staphylococci (MRSA) are susceptible to lysostaphin. Although the vast majority of S. aureus strains, including MRSA, are susceptible to lysostaphin because this enzyme specifically degrades the pentaglycine bridges of their peptidoglycan, resistant variants do exist. Resistance to lysostaphin may arise through mutations in genes such as femA or femB, which alter the composition of the interpeptide bridge, reducing or eliminating glycine residues and thereby preventing lysostaphin activity. None of this is mentioned in the manuscript.

Response: We apologize for omitting this critical consideration. We will add a discussion section addressing lysostaphin resistance: Mutations in femA/femB genes (encoding pentaglycine cross-bridge synthesis) can reduce Lst's efficacy, though such mutations are rare in clinical isolates. However, these mutants completely lose resistance to methicillin and become hypersusceptible to

many unrelated antibiotics including vancomycin (Van). Additionally, they are less fit and virulent, and become easily treatable with antibiotics in vitro and in vivo. Therefore, Van resistance and Lst resistance have distinct mechanisms with no known overlap, making Lst-based therapies a promising option for Van-resistant infections. The addition is highlighted in the Introduction.

6. The in vivo model presents a major limitation, namely that it does not represent a realistic scenario (Lines 363 – 364), since after implanting the material, the treatment is injected immediately adjacent to it.
Response: We thank you for pointing out this issue. After careful examination of our experimental protocol, we found that the subcutaneous injection adjacent to the implant was executed 24 h post-implantation to allow biofilm maturation. We have corrected this mistake in Fig. 6a and the Method section.

7. Line 373: In what volume was the pellet resuspended before being serially diluted?
Response: We clarify that the pellet (from implant-associated biofilm collection) was resuspended in 1 mL of sterile PBS before serial dilution (10-fold gradients) for CFU counting. This detail will be added to the Methods section (Biofilm CFU Assay) to ensure replicability.

8. Materials and Methods, line 168: Which medium was used to induce biofilm formation?
Response: We apologize for the omission. Biofilm formation was induced using tryptic soy broth (TSB) supplemented with 1% glucose (a standard medium for staphylococcal biofilm induction). This detail will be added to the Methods section (Biofilm Formation Assay).

9. I strongly recommend that the authors perform synergy experiments using both planktonic bacteria and S. aureus biofilms with Lst-EPS(V)$_{90}$ in combination with antibiotics that are ineffective against MRSA biofilms, such as gentamicin and/or ceftaroline. This would provide greater clinical applicability to their antibiofilm strategy.
Response: We agree that synergy studies enhance clinical applicability. We will perform planktonic MRSA synergy assays and pre-formed MRSA biofilm synergy assays using Lst-ELP(V)$_{90}$ in combination with Gentamicin (aminoglycoside, ineffective against biofilms due to poor penetration). As shown in the following figure, Lst-ELP(V)$_{90}$ + Gentamicin quickly reduced the turbidity of planktonic MRSA as compared to Lst-ELP(V)$_{90}$ or Gentamicin alone, indicating the synergistic effect of Lst-ELP(V)$_{90}$ and Gentamicin in killing planktonic MRSA. Similarly, Lst-ELP(V)$_{90}$ + Gentamicin outperformed Lst-ELP(V)$_{90}$ or Gentamicin alone in eradicating MRSA biofilms. These preliminary results suggest that the combination of Lst-ELP(V)$_{90}$ with conventional antibiotics could much more efficiently kill planktonic MRSA and eradicate its biofilms, which is beyond the scope of this manuscript and will be further studied in the future.

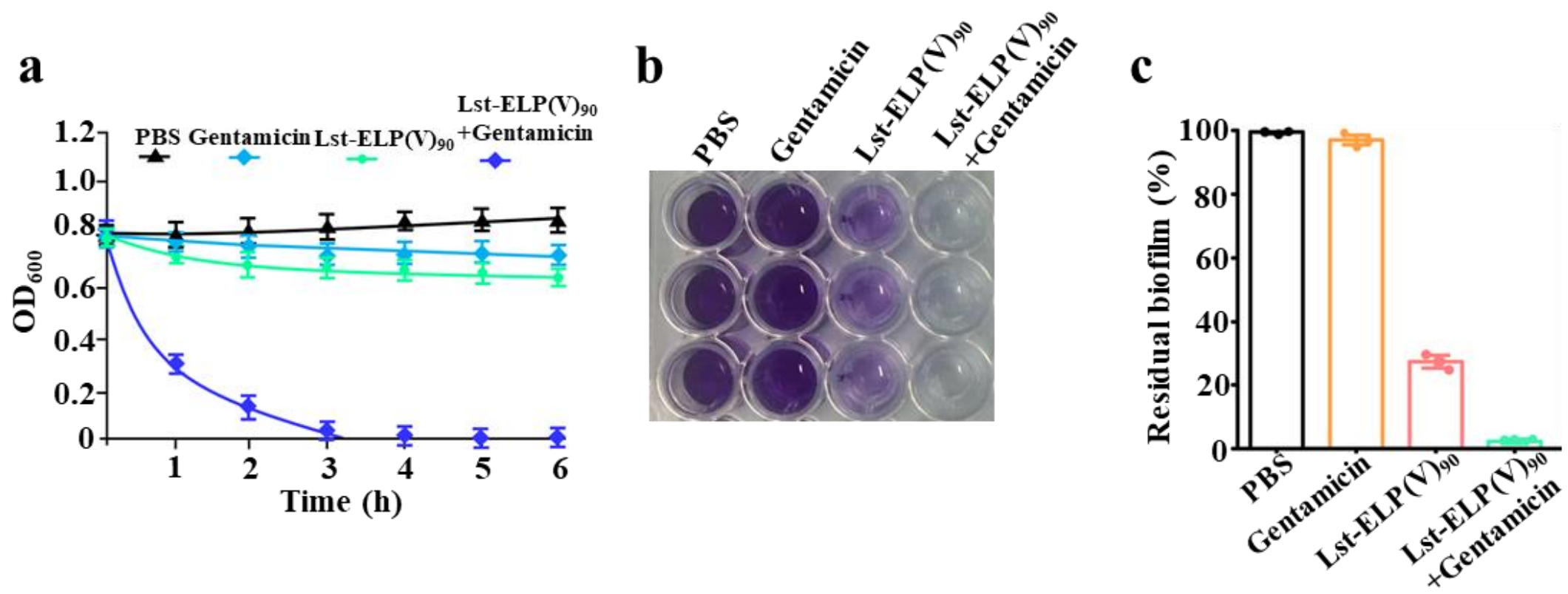

Figure | Lst-ELP(V)$_{90}$ combined with Gentamicin against the planktonic bacteria and biofilm of MRSA. a, Time-killing curve of MRSA strains treated with PBS (control), gentamicin, Lst-ELP(V)$_{90}$, and the combination of Lst-ELP(V)$_{90}$ plus Gentamicin. **b,** Crystal violet staining results of MRSA biofilms following treatment with PBS, Gentamicin, Lst-ELP(V)$_{90}$, and Lst-ELP(V)$_{90}$ in combination with Gentamicin. **c,** Quantification of biofilm eradication rates in the Gentamicin, Lst-ELP(V)$_{90}$, and Lst-ELP(V)$_{90}$ plus Gentamicin groups, respectively. A checkerboard assay was performed to quantify the synergistic interaction against MRSA biofilms, using sub-minimum inhibitory concentration (sub-MIC) of each agent. The sub-MIC values of Lst-ELP(V)$_{90}$ and Gentamicin were 0.25 nM and 4 μg/mL, respectively.

10. The methodology of the bacteremia model is not clear. Was bacteremia monitored in animals with infected implants, or was a separate bacteremia model performed? This is unclear both in the Results and in the Methods.

Response: We clarify that the bacteremia model was integrated with the implant infection model: MRSA detached from implant-associated biofilms naturally disseminated into the bloodstream, and bacteremia was monitored in the same mice used for implant infection (not a separate model). We have revised the Results and Methods (Bacteremia Assay) to explicitly state this integration.

11. The first paragraph of the Discussion repeats substantial information already presented in the Introduction. Please remove it.

Response: We will remove the first paragraph of the Discussion, which repeats background information on MRSA and Lst limitations already presented in the Introduction. The revised Discussion will open directly with the key findings of our study and their implications.

Minor comments

1. The quality of Scheme 1 and Figure 6a is poor for a journal such as Nature Communications. Please redo them with improved aesthetics and visual quality.

Response: We will redo Scheme 1 and Figure 6a with improved aesthetics:

Use high-resolution graphics (vector format) for Scheme 1, with clearer labeling of Lst-ELP(V)$_{90}$'s thermosensitive behavior and biofilm eradication mechanism.

Enhance Figure 6a (experimental timeline) with consistent font sizes, clearer icons, and improved contrast for readability, aligning with Nature Communications standards.

2. In Figures 6b and 7a, which dilution does the image represent? It is difficult to believe that plates with such extensive growth correspond to approximately 10^3 CFU/mL.

Response: We clarify that the images in Figs. 6b and 7a represent the 100 dilutions of the resuspended pellet (for implants) or blood sample. The extensive growth observed is due to the high initial bacterial load (10^6 CFU/mL) and the 100 μL volume plated. We will add a label to each image indicating the dilution factor and plated volume to resolve confusion.

3. In Figure S10, please add a scale bar to each image.

Response: We will add a 1 cm scale bar to each image in Supplementary Fig. 19 (wound healing) to enable accurate size comparison across treatment groups.

4. Throughout the manuscript, the words staphylococci and staphylococcal should not be capitalized and should not be italicized.

Response: We will revise the entire manuscript to ensure:

"staphylococci" (plural) and "staphylococcal" (adjective) are not capitalized and not italicized (consistent with microbiological nomenclature; only the genus/species name *Staphylococcus* is italicized).

5. Materials and Methods, line 356: Where is the method used to infect the implants described?

Response: We apologize for the omission. The method for infecting implants (titanium alloy sheets) have been added to the Methods section (Implant Infection Model): Titanium sheets (1 cm × 1 cm) were incubated in MRSA suspension (10^9 CFU/mL) in LB + 1% glucose for 72 h at 37 °C to form biofilms. During this period, switch to fresh culture medium and continue to cultivate. Biofilm-coated implants were washed with PBS to remove planktonic bacteria before subcutaneous implantation in mice.
